# The sex effect: the prevalence of sex life reasons for contraceptive discontinuation. A systematic review and meta-analysis

**DOI:** 10.1080/26410397.2025.2552589

**Published:** 2025-09-25

**Authors:** Mirela Zaneva, Nandita Thatte, Anne Philpott, Clara Maliwa, Rhiana Mills, Lianne Gonsalves

**Affiliations:** a Junior Research Fellow, Christ Church College, University of Oxford, Oxford, UK; b Technical Officer, UNDP-UNFPA-UNICEF-WHO-World Bank Special Programme of Research, Development and Research Training in Human Reproduction (HRP), Department of Sexual and Reproductive Health and Research, World Health Organization, Geneva, Switzerland; c Founder and Co-Director, The Pleasure Project, Shaftesbury, UK.; d Program Coordinator, The Young and Alive Initiative, Dar Es Salaam, Tanzania; Gender Coordination Specialist, UNDP Tanzania, Dar Es Salaam, Tanzania; e Pleasure Fellow, The Pleasure Project; Researcher, SH:24, London, UK; f Scientist, UNDP-UNFPA-UNICEF-WHO-World Bank Special Programme of Research, Development and Research Training in Human Reproduction (HRP), Department of Sexual and Reproductive Health and Research, World Health Organization, Geneva, Switzerland

**Keywords:** contraception, reproductive health, systematic review, sexual life, method acceptability

## Abstract

The ability to have sex without having an unintended pregnancy is a key motivator for contraceptive use, yet factors related to patients’ sexual lives are critically understudied in contraceptive decision-making. We report the first systematic review and meta-analysis examining the extent to which a contraceptive method’s effect on a satisfying sexual life results in contraception discontinuation or switching. We searched for relevant qualitative and quantitative literature published during 2004–2023 in 9 databases and included 64 studies, comprising a total of 125,586 participants and corresponding to at least 52,705 discontinuers in need, aged 12–54 and surveyed in clinical or public health settings in diverse global contexts. Our main outcome measures were discontinuation or switching of modern contraceptive methods (excluding condoms) while in need. A meta-analysis of 25 studies indicated that sex life concerns as a reason for contraceptive discontinuation have an overall prevalence of 5% (95% CI: 0.03, 0.08; *I*^2^ = 94%, *τ*^2^ = 1.31; *Q* = 381, *p* < 0.001). Sub-group meta-analyses and follow-up meta-regressions do not support any differences based on the hormonal content of the contraceptive method, the specific contraceptive method type, or the specific type of sex life reasons. Sex life reasons are important, common reasons for contraceptive discontinuation. Their prevalence is comparable to other reasons for discontinuation, such as cost or access. Future research should investigate impacts of contraceptive methods on sex life more directly and clinicians should include counselling on a method’s sexual acceptability as part of the wider conversation on common contraceptive side effects.

## Plain language summary

This study explored how the effect of a contraceptive method on people's sex lives might make them decide to stop or change the method. We analysed data from 64 studies conducted globally between 2004 and 2023, involving over 125,000 participants. We found that about 5% of people who discontinued contraception while still needing it did so for reasons related to their sex life. These reasons included decreased libido, discomfort during sex, or concerns about impacts on their partner's sexual experience. The findings highlight that sexual concerns are common reasons for contraceptive discontinuation. These reasons are as common as cost or lack of access, yet they are much less studied. Our review revealed significant gaps in how sexual concerns are measured and reported. Many studies fail to ask users directly about the impact of contraception on their sex lives. Only a few studies explicitly addressed issues like sexual satisfaction or desire. We recommend that future research should include whether contraceptive methods are acceptable sexually. Clinicians should discuss potential sexual side effects with patients and ways to address these. This would help to empower patients and enable them to make more informed choices. Ultimately, this work underscores that sexual well-being is not a secondary concern. It is central to successful contraceptive use. Including sexual concerns as part of contraceptive care can significantly improve user satisfaction. It can reduce discontinuation rates and enhance overall reproductive health outcomes.

## Résumé

La possibilité d’avoir des rapports sexuels sans risque de grossesse non désirée est une motivation essentielle pour l’utilisation de contraceptifs. Pourtant, les facteurs liés à la vie sexuelle des patients sont largement sous-étudiés dans la prise de décision en matière de contraception. Nous présentons la première étude systématique et méta-analyse examinant dans quelle mesure l’effet d’une méthode contraceptive sur une vie sexuelle satisfaisante entraîne l’arrêt ou le changement de méthode. Nous avons recherché des documents qualitatifs et quantitatifs pertinents publiés entre 2004 et 2023 dans neuf bases de données et inclus 64 études, portant sur un total de 125 586 participants et correspondant au moins à 52 705 personnes ayant arrêté la contraception alors qu’elles en avaient besoin, âgées de 15 à 54 ans et interrogées en milieu clinique ou de santé publique dans différents contextes mondiaux. Nos principales mesures étaient l’arrêt ou le changement de méthode contraceptive moderne (à l’exclusion des préservatifs) alors même que le besoin existait. Une méta-analyse de 25 études a indiqué que les préoccupations relatives à la vie sexuelle comme motif d’arrêt de la contraception avaient une prévalence globale de 5% (IC 95%: 0,03,0,08; I^2^ = 94%, τ^2^ = 1,31; Q = 381, *p* < .0001). Les méta-analyses de sous-groupes et les méta-régressions de suivi ne confirment aucune différence fondée sur le contenu hormonal de la méthode contraceptive, le type spécifique de méthode contraceptive ou le type spécifique de raisons liées à la vie sexuelle. Les raisons relatives à la vie sexuelle sont importantes et motivent fréquemment l’arrêt de la contraception. Leur prévalence est comparable à d’autres raisons d’arrêt, comme le coût ou l’accès. Les recherches futures devraient examiner plus directement l’impact des méthodes contraceptives sur la vie sexuelle et les cliniciens devraient inclure des conseils sur l’acceptabilité sexuelle d’une méthode dans le cadre d’une discussion plus large sur les effets secondaires courants de la contraception.

## Resumen

La capacidad de tener sexo sin tener un embarazo no intencional es un motivador clave para usar un método anticonceptivo; sin embargo, se han estudiado muy poco los factores relacionados con la vida sexual de las pacientes en su toma de decisiones sobre la anticoncepción. Informamos sobre la primera revisión sistemática y metaanálisis que examinan en qué medida el efecto de un método anticonceptivo en una vida sexual satisfactoria causa el abandono o cambio del método. Realizamos una búsqueda de literatura cualitativa y cuantitativa pertinente publicada entre 2004 y 2023 en 9 bases de datos e incluimos 64 estudios, que comprenden un total de 125,586 participantes y corresponden a por lo menos 52,705 personas entre 15 y 54 años que abandonaron su método mientras necesitaban uno, quienes fueron encuestadas en entornos clínicos o de salud pública en diversos contextos mundiales. Nuestras principales medidas de resultado fueron abandono o cambio de método anticonceptivo moderno (salvo condones) mientras necesitaban un método. Un metaanálisis de 25 estudios indicó que las preocupaciones de la vida sexual como un motivo para abandonar el método anticonceptivo tienen una prevalencia general del 5% (IC del 95%: 0.03, 0.08; I^2^ = 94%, τ^2^ = 1.31; Q = 381, *p* < .0001). Los metaanálisis de subgrupos y meta regresiones de seguimiento no respaldan las diferencias basadas en el contenido hormonal del método anticonceptivo, el tipo específico de método anticonceptivo, o el tipo específico de motivo relacionado con la vida sexual. Los motivos relacionados con la vida sexual son razones comunes importantes para abandonar el método anticonceptivo. Su prevalencia es comparable con otros motivos de abandono, tales como costo o acceso. Futuras investigaciones deberían investigar más directamente los impactos de los métodos anticonceptivos en la vida sexual y los profesionales clínicos deberían incluir la consejería sobre la aceptabilidad sexual del método anticonceptivo como parte de la conversación general sobre los efectos secundarios del método.

## Introduction

Thirty years ago, the United Nations (UN) International Conference on Population and Development (ICPD) asserted that all couples and individuals have the fundamental right to decide freely and responsibly the number and spacing of their children, as well as their right to have the information, education, and means for family planning.^1^ A key tool that allows for this is the use of contraception. Access to contraception is fundamental to users’ health and rights and allows for women and girls to participate more fully in society, including through education and employment.^2^

The health and development benefits of contraception are well documented.^3^ The availability, choice, and use of contraceptives positively affect various health outcomes, such as reducing maternal mortality rates, reducing the risk of developing certain reproductive cancers, and managing of certain menstrual-related symptoms and disorders.^4,5^ If the need for contraception were met globally, research estimates maternal mortality would drop by 25–35% and the number of unintended pregnancies would drop by two thirds, from 80 million to 26 million.^6–8^ Globally, according to the International Federation of Gynecology and Obstetrics (FIGO) contraceptive use has been associated with increased educational and socioeconomic opportunities for women and girls.^9^

Despite these known benefits, contraceptive discontinuation continues to be a persistent issue in family planning programming. Contraceptive discontinuation refers to those who start using a contraceptive method and then stop using it for any reason while still at risk for unintended pregnancy.^10^ Previous analyses of contraceptive use in more than 30 countries via the Demographic and Health Survey (DHS) indicated that 38% of women in need had discontinued a modern method of contraception; this figure was as high as 50% or more in some countries, particularly in parts of Latin America and Africa.^11,12^ Contraception discontinuation can be associated with negative outcomes for maternal, neonatal, and infant health including greater risk for unintended and high-risk pregnancies, as well as maternal morbidity, and negative psychological effects on women and their children.^13,14^ The reasons for stopping the use of a contraceptive method can be multilevel and intersecting, such as personal preferences, adverse side effects, health concerns, inconvenience or inaccessibility of method, or interpersonal and relationship factors including partner or familial disapproval.^15^ Previous research exploring side effects has focused on medical reasons for discontinuation, such as menorrhagia, intermenstrual bleeding, pelvic pain, infections, headaches, mastalgia, arm pain, depression, and hypertension.^16,17^ Other research exploring systems-level drivers of discontinuation has identified issues around access, supplies, and cost.^18^ Still other research has focused on destigmatising contraception itself as a public health intervention for women and adolescents and young girls, addressing social and religious reservations that exist in many communities.^19^ It should also be noted that discontinuation is not inherently or always negative. The ability to discontinue or switch a contraceptive method when the user finds it unsuitable or undesirable is an essential form of bodily autonomy and personal choice. A related but notably different event is that of contraceptive switching, which denotes stopping the use of one method and starting another. Switching can be seen as a sign of method dissatisfaction while simultaneously also indicating programmatic success, in that it can signify expanded method availability and choice for the user.

Despite the importance of fully understanding contraceptive discontinuation or switching, one area remains conspicuously understudied: stopping contraception due to a method’s impacts on a user’s sex life.^20,21^ This lack of research on contraceptive methods’ sexual acceptability is situated within a larger sex and sexual health gap within sexual and reproductive health and rights (SRHR). Acknowledged by the Guttmacher-Lancet Commission, aspects of sexual health, such as sexual pleasure and well-being, were considered as “largely absent from organised SRHR programmes and their links to reproductive health … understudied”.^22^ Similarly, a previous systematic review has supported the importance of pleasure-inclusive sexual health and rights programming but found a lack of studies from the contraception/family planning field.^23^

Obtaining a richer understanding of the reasons for contraceptive discontinuation is timely given that fewer than five years remain to achieve the 2030 targets for the United Nations’ Sustainable Development Goals, in particular targets 3.7 and 5.6 covering sexual and reproductive health services, including contraception/family planning services. To this end, we have carried out a systematic review and meta-analysis examining the overall prevalence and types of reasons for contraceptive discontinuation or switching pertaining to sexual life concerns. To our knowledge, this is the first systematic review focusing on this topic. We provide evidence that sexual life concerns are an important and common reason for discontinuation.

## Methods

We developed the protocol for this systematic review in accordance with PRISMA guidelines^24^ and pre-registered it in advance on PROSPERO (ID: CRD42023413360).

### Search strategy and databases

We created a search strategy in consultation with experts in sexual health and a specialist subject librarian. We searched for relevant literature in nine databases: PubMed, CINAHL, Sociological Abstracts, PsycINFO, EMBASE, Global Health, Child Development and Adolescent Studies, Web of Science, Scopus. We searched for peer-reviewed papers in English published from January 2004 to May 2023, thus capturing the period since the Reproductive Health Strategy was passed by Member States at the World Health Assembly, representing countries’ commitments to reaching internationally-agreed upon goals and targets related to sexual and reproductive health. We extended the search on 5th December 2023, when we carried out complementary searches in Google Scholar and Elicit following best practices for searches in non-systematic databases.^25^ We also carried out backward and forward citation searching. The details of our search strategy are available in Supplemental Material 1; at a high level our strategy combines MeSH terms and keywords with the following logic: (Contraception) AND ((Discontinuation) OR (Switching)).

### Eligibility criteria

We included quantitative and qualitative peer-reviewed studies in English published during 2004–2023. We accepted various study designs including cross-sectional and cohort studies, as well as interventional and non-interventional work. We note that we had originally pre-registered to survey only non-interventional studies, but we updated our pre-registration following a short scoping review to include interventional trials in some cases. Specifically, for interventional studies, we included efficacy and acceptability studies only. If an interventional trial provided novel or experimental contraception not otherwise available to the general public, we excluded these studies unless they had a control arm providing a widely available, non-experimental contraceptive option. We excluded interventional trials that tested health systems, provider or contraceptive-user targeted informational or behavioural change interventions as we considered that such studies may be more divorced from lived experience and have more limited generalisability.

Our population of interest is contraception users in need of pregnancy prevention and of reproductive age (15–49 years) who have taken and subsequently discontinued or switched from a given contraceptive method. We consider participants to be “in need” if they currently do not wish or plan to be pregnant.^10^ If a given study’s age range was partially outside the standard reproductive age range, we attempted to extract a relevant sub-sample or otherwise retained the sample as long as the mean age still fell within 15–49 years. We accepted all widely available contraceptive methods, regardless of whether they were hormonal or not, or their mode of administration or efficacy. The sole exception to this is male condoms, which we excluded as we consider them to be a separate literature examined elsewhere,^26^ where discontinuation or non-use because of sex life concerns (e.g. the perception that condoms adversely affect the feel of sex) is well explored. If a study included condoms in addition to other methods, we retained such studies (i) for the meta-analysis, if we could clearly disaggregate discontinuation/switching based on different contraceptive methods, and (ii) for the narrative synthesis, if we could either disaggregate as above or if it was clear that condom users were the minority (49% or less) of the entire sample. We included all studies of contraception discontinuation (complete cessation of a method) or switching (cessation of one method and start of another) without imposing any standard for minimum time spent on a previous contraceptive method. For the purposes of the meta-analysis only, we included only studies where reasons were self-reported by contraception users.

Finally, as our review seeks to elucidate the prevalence of reasons for discontinuation tied to real or perceived concerns about the method’s effect on one’s sex life, we required as an inclusion criterion that studies directly assess such reasons. We did not exclude studies based on their reported measure for the effect of interest (e.g. percentage, proportion, count data), but rather accepted all that contained enough information to calculate prevalence. We define factors relevant to a user’s satisfying sexual life in accordance with an existing conceptual model of sexual acceptability of contraception by Higgins & Smith.^20^ Specifically, we accepted any relationship factors (sexual communication; motivations for sexual activity; relationship type, stage, and dynamics; concerns for partner’s pleasure and functioning) or any individual factors (sexual functioning, especially libido; sexual identity; sexual preferences/sexual aesthetics; sexual repercussions of seemingly non-sexual side effects; pleasure and pleasure-seeking). We note that we excluded factors that might be considered as “sexual repercussions of seemingly non-sexual side effects” such as acne, weight gain, bleeding, unless participants had explicitly identified these as sexual concerns. For the purposes of this review, we excluded macro-level factors from the Higgins & Smith model (e.g. culture, social inequality).

### Study selection and data extraction

The first author implemented the search strategy across all databases and then de-duplicated results in Rayyan, where we hosted abstract screening. Each paper was independently assessed for inclusion by two raters. MZ was the first rater for all (100%) of papers at both abstract and full text stages. For abstract screening, CM and RM each read random samples comprising 15% of all abstracts, AP and NT each read random samples comprising 20%, and LG read the remaining 30% of abstracts. For full text screening, CM, RM, AP each read a random sample of 10% of all retained full texts, NT read a random sample of 30%, and LG read the remaining 40% of texts. At both stages all rater pairs retained good inter-rater reliability (minimum of Cohen’s *κ* = 0.75). MZ independently extracted data based on a pre-defined form, collecting information for the surveyed populations and contraceptive methods, such as demographics, method type and (non-)hormonal content. LG also extracted data for a random sample of 20% of the studies included. Data from each extracted study were double-checked independently by NT or CM. Disagreements at any stage were arbitrated by a third rater or through whole team discussion. Where data were missing or unclear, we queried authors.

### Risk of bias and publication bias

Each study was independently assessed for quality/risk of bias by two raters (MZ and either AP or RM). We used different tools according to study design, including the Joanna Briggs Institute Critical Appraisal Tool for Qualitative Studies,^27^ the National Heart Lung and Blood Institute’s Quality Assessment Tool for Observational Cohort and Cross-Sectional Studies and their Quality Assessment of Controlled Intervention Studies in the case of non-randomised interventional studies. For randomised controlled studies, we used the Cochrane risk-of-bias tool for randomised trials (RoB 2). We harmonised the overall scores on a scale of Poor–Fair–Good. We used the overall scores in a univariate meta-regression and sub-group meta-analysis to clarify whether effects varied according to a study’s quality. We examined potential publication bias via funnel plot visualisation and Egger’s test.

### Data analysis

Quantitatively, we considered all included studies with available numerical data for our meta-analysis, that were reported by contraception users themselves, and that did not include male condoms, and focused on identifying comparable studies based on whether they examined discontinuation or switching. To calculate the proportion of sex life considerations linked to discontinuation, we divided the number of discontinuations due to relevant reasons by the total number of discontinuation events, whilst excluding the number of events linked to wanting or planning a pregnancy (i.e. excluding those not in need). We expected considerable heterogeneity amongst studies and so planned to carry out an inverse variance random effects meta-analysis and present results via forest plots. As pre-registered, we were interested in examining potential sub-group effects, related to the type of contraception (hormonal vs non-hormonal, specific method), the specific reasons for discontinuation or their level (individual vs relationship), or any participant characteristics. If there was insufficient data for such a sub-group analysis, we aimed to consider any otherwise relevant characteristics qualitatively. We used the software R, relying chiefly on the package meta.^28^

Qualitatively, we reviewed all included studies and narratively synthesised them, focusing on providing a thematic description that addresses our primary research questions, as well as elucidating how reasons for discontinuation were measured. For the purposes of the narrative synthesis, we organised the descriptions of comparable studies or sub-populations (e.g. users of same method) into logical categories and in discussion and agreement across the reviewer team, analysed and synthesised the findings within each category, while also highlighting key methodological challenges.

### Author reflexivity

Our authorship group comprises six cisgender women with different professional and personal backgrounds who are currently based in the UK, Switzerland, and Tanzania. As a group, we represent different career stages, ethnicities, nationalities, sexual identities, and occupations within health-oriented intergovernmental organisations, academia, and civil society. As an authorship group, we recognise our positionality and privilege working in our respective global and local spaces and are committed to amplifying locally identified research gaps like sexual pleasure on a global scale. Given the importance of meaningful youth engagement across global public health, two young researchers were purposefully involved throughout each stage of the review and joined this publication as co-authors. Lived experience has also helped inform this research. The author group has used various contraceptive methods to date, including male and female condoms, the vaginal ring, oral contraceptive pills, emergency contraceptive pills, the intrauterine system, the diaphragm, and has a combined lived experience of contraception use of over 45 years.

This research responds to previous assessments and engagement activities conducted by the authors, civil society organisations, and others working in SRHR research and programmes on pleasure-based sexual health, which have consistently pointed to the need for more inclusion of sexual well-being as part of current efforts in sexual and reproductive health and rights. The dissemination of results will help to inform and shape future contraception and family planning efforts and to elevate the need for sexual health concerns as a core component of future SRHR research and programmes.

## Results

**Figure 1.**
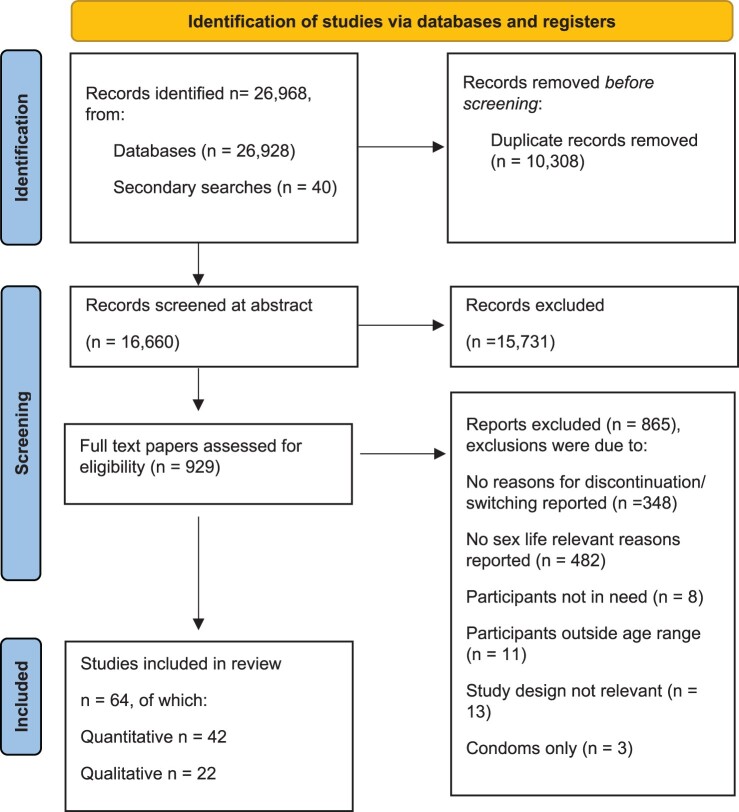
PRISMA flowchart depicting study selection procedure

We screened 16,660 abstracts for relevant context (contraception), appropriate design, and population and retained 929 articles for full text screening. We excluded 348 papers as they did not report any reasons for discontinuation or switching. Of the remaining 581 papers that we considered, which reported any reasons for discontinuation or switching, we excluded a further 482 as they did not report any sex life relevant reasons for discontinuation or switching. In other words, we excluded 83% (482/581) of papers measuring discontinuation because they had not examined sex life relevant reasons. After further exclusions based on age range and design (*k* = 35 studies), we ultimately retained 64 papers corresponding to over 125,584 participants surveyed, with an age range of 12–54 (see Figure 1 for flowchart). While some studies (particularly qualitative designs) did not transparently report discontinuation while in need, we estimate our full sample of included studies to represent a minimum of 59,926 people who discontinued or switched contraception, and at least 52,705 people who did so while in need. Study characteristics of the included papers are presented in Table 1 for quantitative studies and Table 2 for qualitative studies.

### Quantitative results

**Figure 2.**
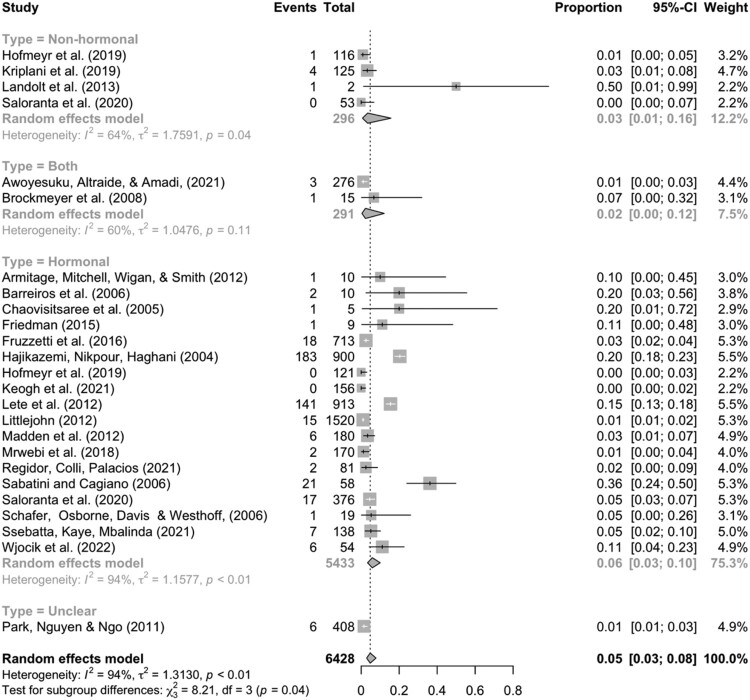
Forest plot presenting results for sub-group analyses based on the surveyed contraceptive methods’ hormonal versus non-hormonal content Notes: Studies are identified with a short citation. “Events” denotes the number of cases of discontinuation due to sex life concerns reported in a given study. “Total” refers to the total number of discontinuation cases reported by users in need. “Proportion” thus refers to the proportion of relevant discontinuation cases due to sex life concerns from the total number of discontinuations.

We were able to meta-analyse 25 studies on discontinuation representing 6428 participants who were in need and discontinued contraception, of whom 440 discontinued due to sex life relevant reasons. Our random effects model indicated that the overall prevalence of discontinuation while in need due to sex life relevant reasons is 5% (95% CI: 0.03, 0.08; *I*^2^ = 94%, *τ*^2^ = 1.31; *Q* = 381, *p* < 0.001; see Figure 2).

Regarding publication bias, funnel plot examination (see Supplemental Figure 1) and an Egger’s test (*p* < 0.001) indicate the presence of some asymmetry. Following Duval and Tweedie’s trim-and-fill procedure, we observe an imputation of 11 studies. Pooling the overall prevalence after this procedure yields an overall prevalence that is now larger at 14% (95% CI 0.09–0.22; see Supplemental Figures 1B and 2 for updated funnel and forest plots). We recommend this result is taken tentatively, as most reasons for discontinuation are presented as descriptive data, rather than the focal result in our included studies (e.g. acceptability of method) around which we might anticipate publication bias to occur.

#### Sub-group meta-analyses and meta-regressions

We were also able to carry out some of our key pre-registered sub-group analyses, namely those examining potential differences between contraceptive methods based on hormonal content and specific method, as well as potential differences in the types of discontinuation reasons according to the Higgins & Smith^20^ framework and their level (i.e. reasons primarily affecting the individual vs relationship). As an overall note, we caution that in some cases some of the sub-group analyses contain few observations and/or are based on few datapoints, and that different sub-groups may be underpinned by both different quality and different quantity (number of studies) of evidence and should be treated cautiously.^29^ To further clarify each sub-group result, we carried out a series of univariate meta-regressions. We also carried out a multivariate regression accounting for both hormonal content and specific contraceptive method (see Supplemental Material 2 for all results).

In Figure 2, we present a forest plot with sub-group categorisation based on whether a study examined solely non-hormonal methods, solely hormonal methods, both types of methods (without the possibility of disaggregation), or methods that were unclear. This captures the same data as our main meta-analysis (i.e. 25 studies, 6428 discontinuers, of whom 440 due to sex life relevant reasons). All four of the non-hormonal data points are based solely on copper IUD use. By contrast, the hormonal studies cover a wider range of methods such as the vaginal ring, implant, pill, injection, the LNG-IUS. The overall prevalence estimate of non-hormonal method discontinuation due to sex life relevant reasons is 3% [95% CI 1%–16%], and that for hormonal method discontinuation is 6% [95% CI 3%–10%]. Although an omnibus chi-squared test indicates a presence of statistically meaningful sub-group differences (*p* = 0.04), the pairwise contrast between hormonal and non-hormonal methods does not reach significance at a standard alpha level (0.05) in a follow-up meta-regression (see Supplemental Material 2).

**Figure 3.**
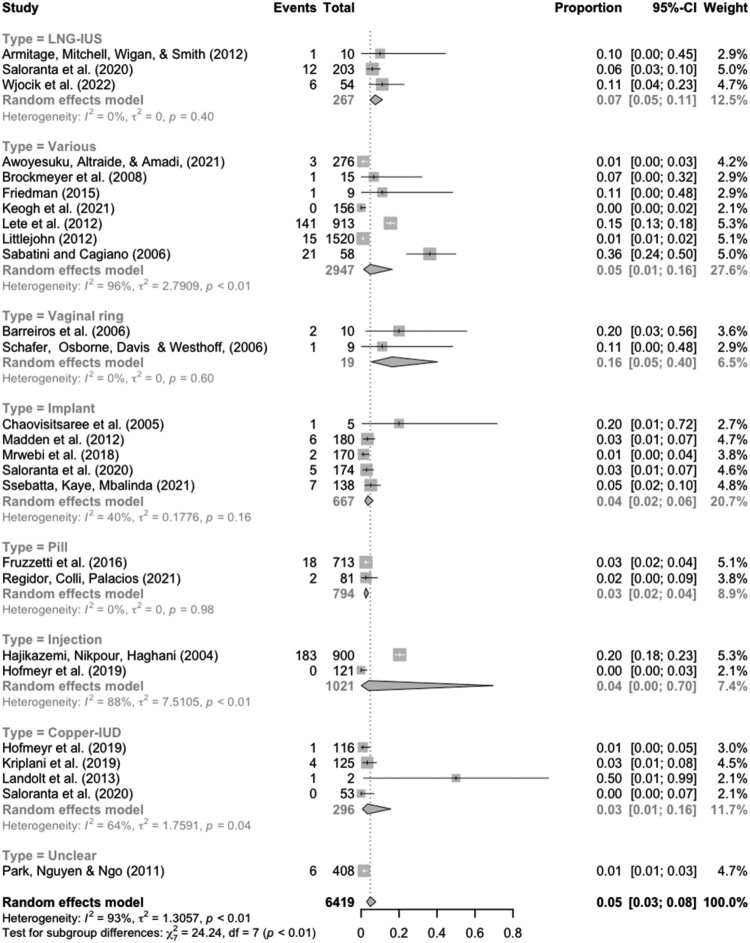
Forest plot presenting results for sub-group analyses based on the specific surveyed contraceptive methods. Studies are identified with a short citation Notes: Studies are identified with a short citation. “Events” denotes the number of cases of discontinuation due to sex life concerns reported in a given study. “Total” refers to the total number of discontinuation cases reported by users in need. “Proportion” thus refers to the proportion of relevant discontinuation cases due to sex life concerns from the total number of discontinuations.

Figure 3 represents a forest plot with sub-group for the specific type of contraceptive method used representing a sample total of 6419 discontinuers in need, of whom 440 were due to sex life relevant reasons. An omnibus chi-square test suggests there are differences between groups (*p* = 0.001). The prevalence estimates are: 16% for the vaginal ring [95% CI 0.05–0.40], 7% for users of levonorgestrel intrauterine systems (LNG-IUS) [95% CI 0.05–0.11], 3% for the non-hormonal copper-IUD discontinuation [95% CI 0.01–0.16], 4% for implants [95% CI 0.02–0.06], 4% for the injection [95% CI 0.00–0.70], 3% for the pill [95% CI 0.02–0.04). Follow-up meta-regression does not provide evidence for statistically significant pairwise differences against non-hormonal IUD.

We present forest plots for sub-group analyses by the category (see Supplemental Figure 3) and level of reason (see Supplemental Figure 4) according to the Higgins & Smith^20^ framework. Neither chi-square tests nor follow-up meta-regressions support the presence of statistically significant sub-groups differences by category or by level. Regarding the category of reasons, we disaggregate the specific reason for discontinuation into concern for sexual functioning, especially libido (prevalence estimate: 4% [95% CI 0.02–0.09), concern for partner’s pleasure and functioning (6% [95% CI 0.02–0.14]), and sexual preferences/sexual aesthetics (6% [95% CI 0.01–0.30]). Regarding the level of reasons, we disaggregate these into concerns primarily affecting the relationship (prevalence estimate: 6% [95% CI 0.02–0.14]) or concerns primarily affecting the self (4% [95% CI 0.02–0.08]).

#### Risk of bias

Regarding quality assessment, we found that the methodological quality of studies varied (see Supplemental Material 3). In terms of quantitative studies, randomised trials generally had at least some concerns, which most often included non-balanced groups and challenges with the random allocation. Most cohort studies were of fair overall quality; typical concerns included low response rates and attrition, insufficient detail for recruitment strategies, and conflict of interest concerns, such as provision of funding by contraceptive companies. We found most qualitative studies to be of good quality. The most common issues were a lack of reflexivity and for some papers, insufficient detail on the analytical approach. To further assess the potential impact of risk of bias, we have carried out a sub-group meta-analysis, grouping studies by their overall score (see Supplemental Figure 5). The omnibus chi-squared test for this was not significant (*p* = 0.85). This is further corroborated by the non-significant results of a univariate meta-regression for risk of bias (see Supplemental Material 2). Overall, we do not find evidence for effect size differences between studies based on their overall risk of bias score.

### Narrative synthesis

Our included population is almost exclusively female contraception users (age 12–54) surveyed in clinical or public health settings and reporting contraceptive discontinuation or switching. Studies were implemented in a wide range of globally diverse contexts and we acknowledge that discussion about sex life concerns can be sensitive if not taboo in some settings. In two papers, relevant sex life reasons for discontinuation were reported only by male partners.^30,31^ A further three papers provide women’s own reasons for discontinuation, in addition to reasons from health workers^32^ and male partners.^33,34^ Although we had pre-registered our interest in examining a number of further characteristics that we considered as potentially important to understand the relationship between sex life concerns and discontinuation, such as sexual orientation, ethnicity, socioeconomic status, marital/relationship status, these data were not reported for the majority of papers surveyed, and we are therefore unable to reflect on the experiences of users with these different characteristics. Qualitatively, we noted that physical or clinical factors, which we had not considered in our pre-registration, such as weight and BMI, tended to be reported more frequently.

The vast majority of included papers, *k* = 63 (or approximately 98% of all included papers), surveyed discontinuation. We found switching was comparatively understudied with one paper examining switching exclusively (∼1%), and a further three papers (∼5%) considering both switching and discontinuation. In four of the discontinuation papers, switching or desire to switch to a different method were considered reasons for discontinuation. We have data on non-hormonal methods from 21 studies, and on hormonal methods from 56 studies (some of which also include non-hormonal methods); for a further three studies the method used was an intrauterine device, but it was not specified whether this was copper or hormonal.^35^ Our review includes various specific contraceptive methods, such as the copper and hormonal IUDs, the diaphragm, the cervical cap, the contraceptive pill, injection, implant, vaginal ring. As a range, we see discontinuation for sex-relevant reasons of (any) hormonal method as low as 0%^36^ and as high as 36.2%;^37^ for (any) non-hormonal method this was as low as 0.1%^36^ and as high as 50% (^38^ note based in a sample of 2 discontinuers in need).

Discontinuation and switching tend to be primary outcomes in the majority of included studies. Importantly, however, reasons for discontinuation or switching tend to be reported descriptively and are rarely the central inferential focus of the papers (as opposed to, for instance, predicting method acceptability, examining important personal or social determinants associated with discontinuation). With this and the differing research designs in mind, reporting and measurement of reasons is varied (see Supplemental Tables 1 and 2). In some studies, participants could only report one reason for discontinuation/switching, and this was usually referred to as the “main” or “most important” reason^40–42^. In other cases (less often in quantitative designs and more frequently in qualitative designs), participants were able to report a variety of reasons that shaped their decision-making process, as well as to further contextualise or compare them in terms of relative importance for various aspects of their lives.^42,43^ In some studies, it was not clear how many reasons participants could report.^45,46^ It was difficult to gain a systematic understanding of the most common ways researchers prompted for reasons. In qualitative work, few studies employed semi-structured interview questions and in general most studies accepted free responses from participants (particularly in in-depth interviews and focus groups), which would be thematically organised and sometimes quoted partially or in full. By contrast, it was not possible to clearly identify in most quantitative studies whether participants were provided with pre-existing categories and could choose reasons amongst them, or whether they were able to provide a free response, which was then categorised. As a common thread, most studies asked for general discontinuation reasons, and only a minority of studies had the *primary* purpose to survey a specific discontinuation reason (e.g. bleeding^47^). Surprisingly, we found only two studies in which participants were directly and specifically asked how contraception impacted their sex lives and in turn, how sex life concerns were associated with discontinuation (libido^48^ and desire and sexual satisfaction^38^).

In terms of reporting, researchers often pooled reasons into broad categories which we could not disaggregate. For instance, some studies reported discontinuation due to “side effects” or “other reasons”, which could encompass as much as 30% of all reported reasons for discontinuation.^48^ Looking specifically at sex life concerns, the most common individual-level reasons related to sexual functioning, especially decrease or loss of libido. The most common relationship-level reasons related to concerns about the partner’s pleasure and functioning, which was most often associated with partner feeling the contraceptive device (IUD, ring) during intercourse.

Further, in most of our included papers, the reasons provided by discontinuers/switchers were aggregated irrespective of method used, thus not allowing for linkage of specific reasons to specific methods (see Supplemental Tables 1 and 2). For instance, although we have included 19 studies surveying the copper IUD (the most common non-hormonal method we captured), we are able to link specific reasons to copper IUD discontinuation in less than a third of cases, i.e. in only six studies (due to discomfort during sex, *k* = 2; due to partner feeling the IUD, *k* = 2; due to rumours it impedes sex, *k* = 1; due to problems with libido, *k* = 1). Similarly, although we have data on hormonal contraceptive methods from 56 studies, we can only link specific reasons to specific methods in 30 papers. For instance, implant discontinuation was examined in 31 studies but in only 11 studies attribution to specific reasons was possible, where the most common reason was decrease or loss of libido (in *k* = 7 studies). Discontinuation of the vaginal ring was surveyed in 12 papers, of which 8 studies allowed for specific reasons to be linked with discontinuation of the vaginal ring, which was most often discontinued due to the male partner feeling the ring during intercourse (*k* = 6).

## Discussion

### Main findings

Here, we discuss four key findings: (1) sex life reasons are important and common reasons for contraceptive discontinuation, (2) they are comparable in prevalence to discontinuation due to other reasons such as accessibility or cost, (3) sex life reasons are rarely surveyed, and (4) when they are surveyed, varied challenging measurement issues are present.

We provide evidence that sex life concerns are an important and noticeable group of reasons for contraceptive discontinuation. Based on our meta-analysis, we find sex life concerns as a reason for contraceptive discontinuation have an overall average prevalence of 5% (95% CI: 0.03, 0.08; *I*^2^ = 94%, *τ*^2^ = 1.31; *Q* = 381, *p* < 0.001). A one in 20 chance is described as “common” according to various medical bodies and institutions, such as by the European Commission medical guidelines^49^ or the United Kingdom’s National Health Services.^50^ Although it is likely more women have experienced sex life concerns than those who have discontinued due to these reasons, our findings demonstrate that considering a method’s effect on a user’s sex life is worthy of similar rigorous consideration as other comparably frequent reasons for discontinuation.

For instance, compared to pooled discontinuation rates from 36 low and middle-income countries in the Demographic and Health Surveys, a 5% discontinuation rate is similar to the rates of discontinuation due to cost (3.5%) and lack of access (4.7%).^19^ Significant financial, time, logistical and research investments have been made to address these issues, such as through vouchers for free or reduced cost contraceptives, which have shown to increase use of contraceptives use in some settings.^51,52^ Further, the provision of free services can also increase uptake and use, particularly for methods that are typically more costly like long-acting reversible contraceptives.^53^ With regard to access, investments in mobile outreach services,^54^ strengthening access to services through community health workers^55^ and task sharing have all indicated improved use.^56^ Given that our findings show that 5% of users discontinue due to sex-related reasons, similar efforts and investment to address the sexual acceptability of contraception should be made.

To address discontinuation due to sex life reasons while in need, the family planning and contraception community must also invest in better and more direct measurement of sexual concerns and sexual acceptability. Our review indicated that sex life impacts are rarely measured in the discontinuation literature: we excluded 83% (482/581) of papers that had measured discontinuation but had not examined *any* sex life relevant reasons. Further, in our included sample only two studies had directly, as a focal aim, asked about contraception’s impacts on sex lives, specifically on libido^47^ and sexual satisfaction.^37^ Including sex life considerations in the study of contraceptive decision-making is a fundamental and tractable next step for research, given that a key motivator for contraceptive use is the ability to have safe sex.^57^ Sex life impacts are currently critically understudied in the contraception field.

Importantly, increased inclusion of sex life impacts should be paired with higher overall reporting standards. Our review was challenged by papers which grouped different reasons in broad categories in ways that were not transparent and did not allow for disaggregation; this is in line with the results of previous research where the most cited reasons for discontinuation of modern methods were reported in broad categories like “side effects and health concerns” (40.2%; 19) with little clarity on what these reasons truly entail. Indeed, one paper we reviewed reported “other reasons” for discontinuation which were cited over 30%.^48^ In some cases, users themselves may prefer to report side effects as a reason for discontinuation rather than further specify. In other cases, however, users clearly reported more specific reasons but researchers aggregated these in broader categories (see Supplemental Tables 1 and 2 for examples of included papers using such categories). Our findings are in line with a previous critical review which examined the use of such broad categories and found that categories such as “other reasons” may pool together substantively very different reasons for discontinuation, including impacts on sexual life, change in preferences, or allergies.^21^ Similarly, this review found that although categories like “side effects” tend to capture medically undesired effects, most often bleeding, the degree and nature of individual reasons is not always clear. Indeed, varied impacts such as headaches, bloating, acne, bleeding, and dryness can also impact a user’s sex life but both the specific side effect as well as any potential secondary effects are not assessed or transparently measured in the primary literature we examined. Information on sexual function and acceptability while using contraception is sorely lacking and future research efforts should be more proactive in trying to understand these important user perspectives around contraceptive use. We recommend more targeted measurement of sex life impacts, clearer reporting of reasons (i.e. distinguish between cases where users prefer to report reasons in broad categories and cases where researchers prefer this), and allowing for more nuance. This could happen particularly by directly asking contraception users whether sex-related impacts contributed to their decision to switch or discontinue and allowing users to provide their reasons for discontinuation (rather than selecting from a pre-defined list), and letting users select more than one reason that contributed to discontinuation.

### Strengths, limitations, and future directions

In terms of strengths, to our knowledge this is the first systematic review and meta-analysis aiming to understand the types and prevalence of sex life concerns as reasons for contraceptive discontinuation or switching. Previous research has suggested that women of reproductive age are overlooked in sexual and reproductive health and rights interventions that incorporate sexual pleasure consideration.^26^ We hope that our current work shines a light on the extent to which multifaceted sex life factors can influence contraceptive decision-making.

A further strength of our review is the inclusion of varied, global data surveying a wide range of contraceptive methods, as well as the ability to consider it carefully, albeit tentatively, across sub-group analyses. We do not find evidence for different prevalence rates of sex life reasons between hormonal versus non-hormonal methods, or between varied specific methods, or between different categories or levels of reasons. While specific reasons may vary, it is possible that the prevalence of general sex life concerns leading to discontinuation, are consistent between methods. Some of our estimates (particularly for specific methods) are based on few studies, and so future research (or even disaggregated reporting of existing data) would better elucidate potential differences.

In terms of limitations, our data and inferences are first limited by some overall trends in the literature. It is possible we have missed relevant studies that had measured sex life reasons but did not report these transparently, such as for instance where reasons were aggregated in broad categories such as “side effects” or “other reasons” or “husband/partner opposition” (as described above). Previous work has reported categories like “other” can and do sometimes contain effects such as low libido.^21,58^ Similarly, for our sub-group analysis examining specific contraceptive methods, we are able to link specific discontinuation reasons to specific methods in few cases, as studies tend to provide pooled figures for any method discontinuation, thus limiting our inferential ability. To address these two issues and provide more reliable inference, we recommend direct measurement of sex life concerns in future work and greater consideration of sex life concerns in future research.

A further limitation is our use of unintended pregnancy and unmet need as indicators in our review. We recognise current discussions about the need for better measures that include more precision and better capture user values and preferences around fertility and contraceptive use.^59,60^ Unintended pregnancy was chosen as a broader term that encompasses both mistimed and unwanted pregnancy and is consistent with the literature that was reviewed. Our narrow definition of users “in need”, captured those who do not wish to become pregnant. An alternative approach would be to define users “in need” as those who are not currently using contraception but would like to be. Such a definition is important, as it focuses on users’ autonomy and decision-making. For the purposes of our systematic review and meta-analysis, we pre-registered taking a pragmatic approach and defined “in need” in line with existing common frameworks such as by the Demographic and Health Surveys. This allowed us to include papers with a widely shared and surveyed definition of being in need, as we worried that alternative definitions, although more inclusive, would be more difficult to systematically survey. In this sense, we see the estimates we provide here as robust albeit conservative. As the family planning measurement community continues to adapt and adjust terminology, future research should consider newer human rights-based terms and definitions, especially when considering interventions or programmatic implications around family planning and contraceptive use.

As another consideration, it is likely that we are underestimating the overall prevalence of sex life concerns in the context of discontinuation. First, this is because a significant part of the included literature asks for one single reason for discontinuation, whereas contraceptive users tend to consider multiple reasons in their decision-making.^20^ Further, people are likely to conform with sociocultural standards^31,61^ and so, when prompted to ask for only one reason for discontinuation, it is possible that they may under-report sex life reasons and select a more “socially palatable” reason due to the existing social difficulties and even taboos about talking about sex in certain contexts.^62^ Here we have taken a stringent definition regarding what sex-relevant reasons entail to elucidate a clear, plausible, minimum prevalence. However, it is possible we have not captured some relevant reasons in this review. For instance, we have excluded reasons like “husband opposition” where the basis of that opposition is not described, although it is plausible that in some cases these may have also captured relevant data such as a partner feeling IUD strings during sex. Some broader categories such as “method inconvenience”, “pain” or “discomfort” could also in some cases include sex life impacts, but we have excluded these unless users had directly stated them as sex life reasons. Similarly, we have excluded reports of bleeding-related (or dryness, pelvic pain, itching-related) discontinuation unless this was explicitly stated as impacting sex life, although the mechanisms through which bleeding-related changes may impact daily life (including one’s sexual life) could, in many instances, be overlapping.^63,64^

Most of the included studies survey discontinuation, so there is a comparative knowledge gap for switching. We were unable to find a sufficient number of papers that survey sex life considerations in the context of switching contraceptive methods to carry out a meta-analysis, and so consider this an understudied question. It remains important to better understand why people switch between methods and to provide relevant counselling, including choice of different methods. Understanding why users stop using methods is important in the context of having more user-centred counselling, better informed method refinement and development, and (at the individual level) helping to guide a user towards a method that won’t result in the same issue. To that end, when a user switches from one method to another, that does mean they have decided to stop one method, and it remains important to understand the reasons for this choice.

We also found more data on hormonal rather than non-hormonal methods, which warrant further consideration. The most widely surveyed non-hormonal method was the copper IUD, so there are gaps for other non-hormonal alternatives, such as the diaphragm or cervical caps. Further, studying switching and a wider range of hormonal methods will allow for a fuller understanding of the different factors of influence and outcomes related to contraceptive decision-making. Moreover, it is important to acknowledge that reasons for discontinuation may vary between people and their circumstances. For the purposes of this review, we have focused solely on reported reasons and have not examined other explanatory factors, such as economic and social determinants, which remain an important piece of understanding contraceptive use.

Importantly, most studies that considered a sex life-related reason, tended to examine only one reason (typically sexual desire/libido), whereas it is possible that different aspects of sexual life are differentially important in contraceptive decision-making. With this in mind, a wider consideration of more varied sex life factors could be beneficial for future work. Regarding the sub-group results for category and level of discontinuation reasons, we grouped studies according to an existing model of sexual acceptability, which was created and then validated via a narrative review approach.^20^ This is different from quantitative practices, where latent constructs are devised by data-driven approaches such as principal component analysis, which reduce dimensionality. Therefore, it is possible that some categories (e.g. pleasure and pleasure-seeking and sexual functioning, especially libido) and levels (individual and relationship) overlap, so we have not meaningfully distinguished between different categories of studies.

### Research and programmatic implications

The findings of this study have research and programmatic implications for strengthening sexual and reproductive health and rights. By highlighting the significance of a method’s interaction with a user’s sex life in contraceptive research and programming, this study contributes to a more comprehensive and rights-based approach to SRHR, an important gap and need identified by the 2018 Lancet commission on SRHR.^22^ We present a summary of key research and programmatic implications in Box 1. We believe there are concrete opportunities to improve research methods by focusing on the need to better measure reasons for discontinuation and switching on the whole, as well as sex life relevant reasons more concretely. Research should consistently assess and track reasons for discontinuation/switching. Users should be allowed to report more than one reason and should be asked to report reasons in comprehensive, nuanced and non-judgmental ways. This should be complemented by improvements in reporting, such as clear reporting of reasons, ideally without pooling in very broad and uninformative categories (e.g. “other reasons”). Targeted measurement of sex life reasons is warranted, given how critically understudied these reasons are. Users should be asked directly about any potential impacts on sex life and should be given opportunities to explain how any reasons intersect with their sex lives.

Box 1. **Summary of research and programmatic implications**

**Table:** 

Research Implications	1) There is a need for better overall measurement of changes in contraception use which centre user autonomy and perspectives, including their reasons for discontinuation/switching. These should involve: Consistently including an assessment of reasons for discontinuation Asking users for reasons comprehensively, acknowledging a wide, varied spectrum of possible reasons Allowing users to report more than one reason for discontinuation, recognising that reasons may have different comparative importance (e.g. primary vs secondary) Asking users for reasons with nuance, contextual sensitivity, and without judgement, recognising that some reasons may be more/less socially acceptable, that reasons may intersect and overlap Higher reporting standards, such as clear reporting of reasons and not pooling together substantively different reasons in broad categories (e.g. “other reasons” or “side effects”) There is a need for better measurement of sex life reasons specifically, which should involve: Consistently and as standard accounting for impacts on sex life and sexual acceptability of contraceptive methods, such as by asking users directly Consideration of how sex life concerns can operate either as a primary or secondary reasons, or can be embedded or be a secondary effect of other reasons (e.g. bleeding, dryness)
Programmatic Implications	1) Sexual acceptability should be incorporated into family planning and contraception counselling and programming as part of a human rights-based approach; as illustrative examples, this could involve: Improving awareness among providers and the SRHR sector as a whole that contraception use can facilitate a satisfying and safe sex life Systematically including what we know about the sexual acceptability of a method into family planning counselling (e.g. advising users for possible impacts on various aspects of sex life) to ensure informed decision-making Providing support and solutions to address known challenges to sexual acceptability (e.g. offering lubricant for methods which might potentially cause vaginal dryness) Ensuring that providers are trained to normalise discussion about a user’s sex life in the midst of contraception counselling and users are empowered to bring up questions or concerns

In terms of programmatic implications, we recommend strengthening contraceptive counselling to include a consideration of different contraceptive methods’ sexual acceptability. We acknowledge that some healthcare providers may already include counselling around sex life concerns such as about impacts on libido (for example the WHO Family Planning: A Global Handbook for Providers 2022 edition^65^ has limited guidance around how some methods may or may not affect a user’s “sex drive”), or that a partner might feel the ring. Based on programmatic experience and our results, however, this counselling is not consistently and proactively implemented and could stand to be improved. Provider training and contraceptive counselling aids should normalise discussions about sex in the context of contraception, by creating safe spaces in clinical and public health environments where users are empowered to ask questions about the full range of side effects and to select their desired method as a crucial part of promoting sexual health and rights. Providers should foster upfront discussions, while being sensitive to the individuals’ preferences, personal and contextual circumstances, about how different contraceptive methods might affect users’ sex lives. This is important, given that a previous review suggests that women who were counselled on what possible side effects they could expect were less likely to discontinue due to those side effects.^66^ Indeed, the provision of further support to address any challenges or side effects should be offered. For instance, previous work has shown that providing IUD users with NSAIDs can help reduce bleeding and pain.^67^ In this same regard, as one example, users could be offered lubricants for methods for which a known side effect is vaginal discomfort or dryness.

## Conclusion

There is a large body of research surveying reasons for contraceptive discontinuation, yet, despite their importance, impacts on sex life have been critically understudied in this domain. To our knowledge, this is the first systematic review examining the prevalence of sex life reasons as reasons for contraceptive discontinuation or switching. We find that impacts on sex life are common reasons for discontinuation, with an overall prevalence of 5%, which is comparable to rates of discontinuation due to other widely examined factors, such as cost, unavailability/lack of access, and method inconvenience of use. Based on our findings, we advocate for more substantial investments in better understanding, measuring, and supporting what contraception users say matters to them when choosing and using a contraceptive method. This includes any potential sex life issues. This means continued movement away from the historical donor and programmatic focus on fertility control, towards rights-based approaches that centre the motivations and agency of users and what they require as the basis for contraception development and delivery. In the case of better understanding sex life concerns, we suggest several concrete ways this can be done in research and programming, including clearer, targeted measuring of reasons for discontinuation, and improved provider training and user support.

## Supplementary Material

Spanish translation of article

Spanish translation of Supplemental Material

Supplemental Material

## Data Availability

Our analyses are based on already existing available data from other published studies, which can be accessed directly. Tables 1 and 2 in our manuscript present summaries of the extracted key study variables. For greater ease, key extracted data are also available as a csv file, alongside our code on OSF: https://osf.io/ku48p/
