# Supplementary material for: The sex effect: the prevalence of sex life reasons for contraceptive discontinuation. A systematic review and meta-analysis
Source: Sex Reprod Health Matters. 2025 Sep 25;33(1):2552589. doi: 10.1080/26410397.2025.2552589 (PMC12557827; doi:10.1080/26410397.2025.2552589)
Supplement: Spanish translation of article [file ZRHM_A_2552589_SM0097.pdf]

El efecto sexual: prevalencia de motivos relacionados con la vida sexual en la discontinuidad[1] de métodos anticonceptivos. Revisión sistemática y metaanálisis

Mirela Zaneva,[a] Nandita Thatte,[b] Anne Philpott,[c] Clara Maliwa,[d] Rhiana Mills,[e] and Lianne Gonsalves[f]

[a] Investigadora asociada, Christ Church College, Universidad de Oxford, Oxford, Reino Unido

[b] Funcionaria técnica, Programa Especial de Investigación, Desarrollo y Capacitación en Reproducción Humana (HRP) — PNUD-UNFPA-UNICEF-OMS-Banco Mundial, Departamento de Salud Sexual y Reproductiva e Investigación, Organización Mundial de la Salud, Ginebra, Suiza

[c] Fundadora y codirectora, The Pleasure Project, Shaftesbury, Reino Unido. Correspondencia: anne@thepleasureproject.org

[d] Coordinadora de programas, The Young and Alive Initiative, Dar es Salaam, Tanzania; especialista en coordinación de género, PNUD Tanzania, Dar es Salaam, Tanzania

[e] Becaria del programa en placer sexual de The Pleasure Project; investigadora, SH:24, Londres, Reino Unido

[f] Científica, Programa Especial HRP (PNUD-UNFPA-UNICEF-OMS-Banco Mundial), Organización Mundial de la Salud, Ginebra, Suiza

Resumen

La capacidad de tener relaciones sexuales sin riesgo de embarazo no intencionado es un factor clave que motiva el uso de métodos anticonceptivos. Sin embargo, los aspectos relacionados con la vida sexual de las personas han sido escasamente estudiados en la toma de decisiones anticonceptivas. Presentamos la primera revisión sistemática con metaanálisis que examina hasta qué punto el efecto de un método anticonceptivo sobre una vida sexual satisfactoria puede conducir a la discontinuidad o el cambio del método. Realizamos una búsqueda de literatura cualitativa y cuantitativa publicada entre 2004 y 2023 en nueve bases de datos e incluimos 64 estudios, con un total de 125 586 participantes, que representan al menos 52 705 personas de entre 12 y 54 años que abandonaron su método mientras aún necesitaban anticoncepción. Las personas participantes fueron encuestadas en entornos clínicos o de salud pública en diversos contextos del mundo.

Nuestras principales medidas de resultado fueron la discontinuidad o el cambio de métodos anticonceptivos modernos (excepto condones) mientras persistía la necesidad anticonceptiva. El metaanálisis de 25 estudios indicó que las preocupaciones relacionadas con la vida sexual como motivo para abandonar el método anticonceptivo presentan una prevalencia global del 5% (IC del 95%: 0.03–0.08; $I^2 = 94\%$; $\tau^2 = 1.31$; Q = 381; $p < 0.001$). Los análisis por subgrupos y las meta-regresiones posteriores no mostraron diferencias significativas según el contenido hormonal del método, el tipo específico de anticonceptivo o la naturaleza de las motivaciones sexuales. Los motivos vinculados con la vida sexual son causas comunes e importantes de discontinuidad anticonceptiva, con una prevalencia comparable a otros factores como el costo o la falta de acceso. Recomendamos que futuras investigaciones examinen más directamente los impactos de los métodos anticonceptivos en la vida sexual, y que las y los profesionales de la salud incluyan la consejería sobre la aceptabilidad sexual de cada método como parte de la conversación integral sobre los efectos secundarios más frecuentes.

Resumen en lenguaje sencillo

Este estudio analizó cómo el efecto de un método anticonceptivo en la vida sexual de las personas puede influir en su decisión de dejarlo o cambiarlo. Se recopilaron datos de 64 estudios realizados en todo el mundo entre 2004 y 2023, con la participación de más

[1] El término **discontinuidad** se utiliza de manera consistente a lo largo de este artículo para referirse al **cese o terminación del uso de un método anticonceptivo**. Reconocemos que en otros contextos regionales se emplean los términos discontinuación o interrupción del uso anticonceptivo para describir este mismo fenómeno. Esta elección terminológica responde a la adopción de la nomenclatura estandarizada utilizada en la Encuesta Demográfica y de Salud Familiar (ENDES) del Perú, la cual orienta los marcos nacionales de reporte estadístico y de investigación en salud reproductiva.

de 125,000 personas. Se encontró que alrededor del 5% de quienes interrumpieron el uso de métodos anticonceptivos mientras aún los necesitaban lo hicieron por motivos relacionados con su vida sexual, como la disminución del deseo, molestias durante la actividad sexual o preocupaciones sobre cómo el método afectaba la experiencia de su pareja. Los resultados muestran que las preocupaciones sobre la vida sexualson motivos comunes para dejar los métodos anticonceptivos. Estas razones son tan frecuentes como el costo o la falta de acceso, aunque mucho menos estudiadas. La revisión identificó grandes vacíos en la forma en que se miden y reportan estos temas. En muchos estudios no se pregunta directamente a las personas cómo la anticoncepción afecta su vida sexual, y solo unos pocos analizan aspectos como la satisfacción o el deseo sexual. Se recomienda que las investigaciones futuras incluyan de manera explícita la aceptabilidad sexual de los métodos anticonceptivos. Además, las y los profesionales clínicos deberían hablar con sus pacientes sobre los posibles efectos sexuales de los métodos y las formas de abordarlos. Esto contribuiría a empoderar a las personas y a que tomen decisiones más informadas. En última instancia, este trabajo subraya que el bienestar sexual no es un tema secundario: es fundamental para un uso exitoso de la anticoncepción. Incluir las preocupaciones sobre la vida sexualcomo parte de la atención anticonceptiva puede mejorar notablemente la satisfacción de las personas , reducir las tasas de abandono y fortalecer los resultados generales en salud reproductiva.

Palabras Clave:

anticoncepción; salud reproductiva; revisión sistemática; vida sexual; aceptabilidad del método

---

Este texto ha sido traducido del original.

Este documento fue traducido al español por Ahmad Newton. Agradecemos a quienes revisaron la traducción para asegurar que el lenguaje reflejara la terminología comúnmente utilizada en América Latina para estos temas.

Introducción

Hace treinta años, la Conferencia Internacional sobre la Población y el Desarrollo (CIPD) de las Naciones Unidas afirmó que todas las parejas y personas tienen el derecho fundamental de decidir, de manera libre y responsable, el número de hijos que desean tener y el intervalo entre ellos, así como el derecho a contar con la información, la educación y los medios necesarios para planificar su familia.[1] Una herramienta clave para ejercer este derecho es el uso de métodos anticonceptivos. El acceso a la anticoncepción es esencial para la salud y los derechos de las personas, y permite que mujeres y niñas participen más plenamente en la sociedad, por ejemplo, a través de la educación y el empleo.[2]

Los beneficios de la anticoncepción para la salud y el desarrollo están ampliamente documentados.[3] La disponibilidad, la libertad de elección y el uso de métodos anticonceptivos influyen positivamente en distintos indicadores de salud, como la reducción de la mortalidad materna, la disminución del riesgo de desarrollar ciertos tipos de cáncer reproductivo y el manejo de síntomas y trastornos menstruales específicos.[4,5] Si la necesidad de anticoncepción se cubriera a nivel mundial, las estimaciones indican que la mortalidad materna disminuiría entre un 25% y un 35%, y que el número de embarazos no intencionales se reduciría en dos tercios, de 80 millones a 26 millones.[6–8] A nivel global, la Federación Internacional de Ginecología y Obstetricia (FIGO) señala que el uso de métodos anticonceptivos se asocia con mayores oportunidades educativas y socioeconómicas para mujeres y niñas.[9]

A pesar de estos beneficios reconocidos, la discontinuidad del uso de métodos anticonceptivos sigue siendo un desafío persistente en los programas de planificación familiar. Se entiende por abandono o discontinuidad anticonceptiva el hecho de que una persona inicie un método anticonceptivo y luego deje de usarlo por cualquier motivo, mientras continúa en riesgo de un embarazo no intencional.[10] Análisis previos sobre el uso de métodos anticonceptivos en más de 30 países, realizados a partir de las Encuestas Demográficas y de Salud (DHS, por sus siglas en inglés), mostraron que el 38% de las mujeres con necesidad de anticoncepción habían interrumpido el uso de un método moderno; esta cifra alcanzaba el 50% o más en algunos países, especialmente en ciertas regiones de América Latina y África.[11,12] La discontinuidad anticonceptiva puede tener consecuencias negativas para la salud materna, neonatal e infantil, entre ellas un mayor riesgo de embarazos no intencionales y de alto riesgo, aumento de la morbilidad materna y efectos psicológicos adversos en las mujeres y sus hijos e hijas.[13,14] Las razones para dejar de usar un método anticonceptivo pueden darse en distintos niveles y estar interrelacionadas entre sí. Entre ellas se incluyen las preferencias personales, los efectos secundarios adversos, las preocupaciones sobre la salud, la incomodidad o inaccesibilidad del método, y factores interpersonales o de relación, como la desaprobación de la pareja o de la familia.[15] La investigación previa sobre efectos secundarios se ha centrado en los motivos médicos de la discontinuidad, tales como menorragia, sangrado intermenstrual, dolor pélvico, infecciones, cefaleas, mastalgia, dolor en el brazo, depresión e hipertensión.[16,17] Otros estudios que han examinado factores estructurales han identificado problemas vinculados con el acceso, la disponibilidad de insumos y el costo.[18] Asimismo, algunas investigaciones se han enfocado en desestigmatizar la anticoncepción como una

intervención de salud pública dirigida a mujeres, adolescentes y niñas, abordando las reservas sociales y religiosas que persisten en muchas comunidades.[19] Cabe destacar que la discontinuidad no es, en sí misma, un hecho negativo. La posibilidad de interrumpir o cambiar un método anticonceptivo cuando la persona usuaria lo considera inadecuado o indeseable representa una forma esencial de autonomía corporal y de libre elección. Un fenómeno relacionado, aunque claramente distinto, es el cambio de método anticonceptivo, que implica dejar de usar uno y comenzar otro. Este cambio puede interpretarse tanto como un indicador de insatisfacción con el método anterior como un indicador de éxito programático, en la medida en que refleja una mayor disponibilidad y diversidad de opciones para las personas .

A pesar de la importancia de comprender a fondo la discontinuidad y el cambio de métodos anticonceptivos, existe un aspecto que sigue siendo notablemente poco estudiado: dejar de usar métodos anticonceptivos debido al impacto del método en la vida sexual de la persona usuaria.[20,21] Esta falta de investigación sobre la aceptabilidad sexual de los métodos anticonceptivos forma parte de una brecha más amplia en torno a la actividad sexual y la salud sexual dentro del campo de la salud y los derechos sexuales y reproductivos (DSDR). Tal como reconoció la Comisión Guttmacher-Lancet, aspectos de la salud sexual como el placer y el bienestar sexual han estado "en gran medida ausentes de los programas organizados de DSDR y de sus vínculos con la salud reproductiva… y se han estudiado poco" (traducción del autor).[22] De forma similar, una revisión sistemática previa respaldó la importancia de incorporar el placer en los programas de salud y derechos sexuales y reproductivos, pero evidenció la falta de estudios provenientes del ámbito de la anticoncepción y la planificación familiar.[23]

Profundizar sobre las razones que llevan a la discontinuidad de los métodos anticonceptivos resulta oportuno, considerando que faltan menos de cinco años para alcanzar las metas de 2030 de los Objetivos de Desarrollo Sostenible (ODS) de las Naciones Unidas, en particular las metas 3.7 y 5.6, relacionadas con los servicios de salud sexual y reproductiva, incluida la anticoncepción y la planificación familiar. Con este propósito, realizamos una revisión sistemática y un metaanálisis para examinar la prevalencia general y los tipos de motivos de discontinuidad o cambio de métodos anticonceptivos vinculados con preocupaciones sobre la vida sexual. Hasta donde sabemos, esta es la primera revisión sistemática centrada en este tema. Nuestros hallazgos aportan evidencia de que las preocupaciones sobre la vida sexual representan un motivo relevante y frecuente de discontinuidad anticonceptiva.

Métodos

El protocolo de esta revisión sistemática se elaboró conforme a las directrices PRISMA[24] y se registró previamente en PROSPERO (ID: CRD42023413360).

Estrategia de búsqueda y bases de datos

Diseñamos la estrategia de búsqueda en consulta con personas expertas en salud sexual y con una bibliotecaria especializada en la materia. Buscamos literatura pertinente en nueve bases de datos: PubMed,

CINAHL, Sociological Abstracts, PsycINFO, EMBASE, Global Health, Child Development and Adolescent Studies, Web of Science y Scopus. Se incluyeron artículos revisados por pares, publicados en inglés entre enero de 2004 y mayo de 2023, abarcando el período posterior a la aprobación de la Estrategia de Salud Reproductiva por los Estados Miembros de la Asamblea Mundial de la Salud, que refleja el compromiso de los países con el cumplimiento de metas y objetivos internacionales en materia de salud sexual y reproductiva. La búsqueda se amplió el 5 de diciembre de 2023, con búsquedas complementarias en Google Scholar y Elicit, siguiendo las mejores prácticas para búsquedas en bases de datos no sistemáticas.[25] También se realizaron búsquedas retrospectivas y prospectivas en las referencias citadas. Los detalles completos de la estrategia se presentan en el Material suplementario 1. En términos generales, la estrategia combinó términos MeSH y palabras clave con la siguiente lógica: (Contraception) AND ((Discontinuation) OR (Switching)).

Criterios de inclusión

Se incluyeron estudios cuantitativos y cualitativos revisados por pares, publicados en inglés entre 2004 y 2023. Se aceptaron diversos diseños de investigación, incluidos los estudios transversales y de cohorte, así como estudios intervencionales y no intervencionales. Inicialmente, el protocolo había sido registrado para incluir únicamente estudios no intervencionales; sin embargo, tras una breve revisión exploratoria, se actualizó el registro para incorporar algunos ensayos de intervención de eficacia y de aceptabilidad. Si un ensayo intervencional evaluaba un método anticonceptivo nuevo o experimental no disponible para el público general, dicho estudio fue excluido, a menos que contara con un grupo de control que ofreciera una opción anticonceptiva ampliamente disponible y no experimental. También se excluyeron los ensayos de intervención que evaluaban sistemas de salud o intervenciones informativas o de cambio de comportamiento dirigidas a personal sanitario o a personas usuarias de métodos anticonceptivos, ya que se consideró que este tipo de estudios podían estar más alejados de la experiencia vivida y presentar una menor capacidad de generalización.

La población de interés de este estudio corresponde a personas que utilizan métodos anticonceptivos en edad reproductiva (15 a 49 años) que necesitaban prevenir un embarazo y que habían iniciado y posteriormente interrumpido o cambiado el uso de un método anticonceptivo. Se consideró que las personas participantes estaban "en necesidad" cuando no deseaban ni planificaban un embarazo al momento del estudio.[10] En casos en que el rango de edad de un estudio se extendía parcialmente fuera del rango reproductivo estándar, se intentó extraer una submuestra pertinente o se mantuvo la muestra completa, siempre que la edad media se ubicara dentro del intervalo de 15 a 49 años. Se aceptaron todos los métodos anticonceptivos de uso generalizado, independientemente de si eran hormonales o no, de su vía de administración o de su eficacia. La única excepción fueron los condones masculinos, que se excluyeron por considerarse parte de una literatura independiente, ya ampliamente documentada[26] en relación con la discontinuidad o el no uso por motivos vinculados con la vida sexual (por ejemplo, la percepción de que los condones afectan negativamente la sensación durante la actividad sexual). Cuando un estudio incluía condones junto con otros métodos, se mantuvo en la muestra si (i) era posible desagregar claramente los

datos de discontinuidad o cambio por tipo de método para el metaanálisis, o (ii) si para la síntesis narrativa, era posible dicha desagregación o bien las personas de condón representaban el 49% o menos de la muestra total. Se incluyeron todos los estudios sobre discontinuidad anticonceptiva (cese completo del uso de un método) o cambio de método (abandono de uno e inicio de otro), sin imponer un tiempo mínimo de uso previo. Para el metaanálisis, únicamente se consideraron los estudios en los que las razones de discontinuidad fueron autoinformadas por las personas .

Finalmente, dado que esta revisión tiene como objetivo determinar la prevalencia de los motivos de discontinuidad vinculados con preocupaciones reales o percibidas sobre el efecto del método en la vida sexual de las personas , se estableció como criterio de inclusión que los estudios evaluaran directamente dichos motivos. No se excluyeron estudios en función del tipo de medida utilizada para el efecto de interés (por ejemplo, porcentaje, proporción o recuento de casos), sino que se aceptaron todos aquellos que ofrecieran información suficiente para calcular su prevalencia. Los factores relevantes para una vida sexual satisfactoria se definieron conforme al modelo conceptual existente sobre la aceptabilidad sexual de la anticoncepción propuesto por Higgins y Smith.[20] En concreto, se consideraron tanto los factores relacionales —como la comunicación sexual, las motivaciones para la actividad sexual, el tipo, etapa y dinámica de la relación, y las preocupaciones por el placer o funcionamiento de la pareja— como los factores individuales, entre ellos el funcionamiento sexual (especialmente la libido), la identidad sexual, las preferencias o estética sexual, las repercusiones sexuales de efectos secundarios aparentemente no sexuales, y la búsqueda de placer. Se excluyeron los factores que pudieran clasificarse como “repercusiones sexuales de efectos secundarios aparentemente no sexuales” —por ejemplo, acné, aumento de peso o sangrado—, salvo que las personas participantes los hubieran señalado explícitamente como preocupaciones sobre la vida sexual. Asimismo, para los fines de esta revisión, se excluyeron los factores de nivel macro del modelo de Higgins y Smith (como la cultura o la desigualdad social).

Selección de estudios y extracción de datos

La primera autora implementó la estrategia de búsqueda en todas las bases de datos y eliminó los duplicados en Rayyan, donde se realizó la revisión de resúmenes (abstract screening). Cada artículo fue evaluado independientemente por dos personas revisoras. MZ actuó como revisora principal en el 100% de los artículos, tanto en la fase de resumen como en la de texto completo. Durante la revisión de resúmenes, CM y RM evaluaron muestras aleatorias que representaban el 15% del total de resúmenes; AP y NT revisaron cada una el 20%, y LG examinó el 30% restante. En la revisión de textos completos, CM, RM y AP revisaron cada una el 10% de los textos retenidos, NT revisó una muestra aleatoria del 30% y LG el 40% restante. En ambas etapas, todos los pares de revisoras mantuvieron una buena fiabilidad interevaluador (coeficiente κ de Cohen ≥ 0.75). MZ extrajo los datos de manera independiente utilizando un formulario predefinido, recopilando información sobre las poblaciones estudiadas y los métodos anticonceptivos (datos demográficos, tipo de método y contenido hormonal o no hormonal). LG también realizó la extracción de datos en una muestra aleatoria del 20% de los estudios incluidos. Posteriormente, los datos de cada estudio fueron verificados de forma independiente por NT o CM. Las discrepancias se resolvieron mediante la revisión de una tercera

persona o la discusión en equipo. Cuando los datos eran incompletos o ambiguos, se contactó a las personas autoras correspondientes.

Evaluación del riesgo de sesgo y sesgo de publicación

Cada estudio fue evaluado de manera independiente en cuanto a su calidad o riesgo de sesgo por dos personas revisoras (MZ y, de forma alternada, AP o RM). Se emplearon diferentes herramientas según el diseño del estudio, entre ellas: la Herramienta de Evaluación Crítica del Instituto Joanna Briggs para Estudios Cualitativos[27]; la herramienta de evaluación de calidad del Instituto Nacional del Corazón, los Pulmones y la Sangre (NHLBI por sus siglas en inglés) para estudios observacionales de cohorte y transversales; y su herramienta de evaluación de calidad para estudios de intervención controlados, aplicada en el caso de estudios intervencionales no aleatorizados. Para los ensayos controlados aleatorizados, se utilizó la herramienta Cochrane de riesgo de sesgo (RoB 2). Las puntuaciones globales se armonizaron en una escala de Deficiente, Aceptable o Buena. Estas puntuaciones se emplearon en un metaanálisis por subgrupos y en una meta-regresión univariada con el fin de examinar si los efectos variaban según la calidad del estudio. El posible sesgo de publicación se evaluó visualmente mediante gráficos en embudo (funnel plots) y la prueba de Egger.

Análisis de datos

En el análisis cuantitativo, se incluyeron todos los estudios con datos numéricos disponibles para el metaanálisis, siempre que los resultados fueran autoinformados por las personas usuarias de métodos anticonceptivos y no incluyeran condones masculinos. El análisis se centró en identificar estudios comparables según si examinaban la discontinuidad o el cambio de método. Para calcular la proporción de motivos relacionados con la vida sexual vinculados con la discontinuidad, se dividió el número de discontinuidades debidas a estos motivos por el número total de eventos de discontinuidad, excluyendo los casos asociados al deseo o planificación de un embarazo (es decir, a quienes no estaban "en necesidad"). Se anticipó una heterogeneidad considerable, por lo que se empleó un metaanálisis de efectos aleatorios con el método de varianza inversa, presentando los resultados mediante gráficos de bosque (forest plots). Tal como se estableció en el protocolo, se buscó examinar posibles efectos por subgrupos relacionados con el tipo de anticoncepción (hormonal vs. no hormonal; método específico), los motivos específicos de discontinuidad o su nivel (individual vs. relacional), así como cualquier característica de las personas participantes. En los casos en que los datos disponibles no fueran suficientes para analizarlos por subgrupos, se optó por considerar cualitativamente otras características relevantes. Los análisis se realizaron en el software R, utilizando principalmente el paquete *meta*.[28]

En el análisis cualitativo, se revisaron todos los estudios incluidos y se realizó una síntesis narrativa, con el objetivo de ofrecer una descripción temática que respondiera a las principales preguntas de investigación y de clarificar la forma en que se midieron los motivos de discontinuidad. Para esta síntesis, se agruparon las descripciones de estudios o subpoblaciones comparables —por ejemplo, personas usuarias del mismo método anticonceptivo— en categorías lógicas. A partir de la discusión y el consenso dentro del equipo

revisor, se analizaron y sintetizaron los hallazgos de cada categoría, destacando los principales desafíos metodológicos identificados.

Reflexividad de las autoras

El grupo de autoras está integrado por seis mujeres cisgénero con trayectorias profesionales y personales diversas, que actualmente residen en el Reino Unido, Suiza y Tanzania. En conjunto, representamos distintas etapas de desarrollo profesional, etnias, nacionalidades, identidades sexuales y ámbitos de trabajo dentro de organizaciones intergubernamentales de salud, la academia y la sociedad civil. Como equipo de autoras, reconocemos nuestra posicionamiento y privilegios al desempeñarnos en contextos locales y globales, y asumimos el compromiso de visibilizar y ampliar las brechas de investigación identificadas localmente, como el placer sexual, a una escala global. Reconociendo la importancia de la participación significativa de la juventud en la salud pública global, se incorporó intencionadamente a dos investigadoras jóvenes en todas las etapas de esta revisión, quienes participan como coautoras en esta publicación. La experiencia vivida también ha contribuido de manera valiosa a orientar esta investigación. De forma colectiva, el grupo de autoras ha utilizado diversos métodos anticonceptivos, incluyendo condones masculinos y femeninos, anillo vaginal, píldoras anticonceptivas orales y de emergencia, sistema intrauterino y diafragma, acumulando una experiencia combinada de más de 45 años en el uso de métodos anticonceptivos.

Esta investigación se apoya en evaluaciones y actividades de colaboración previas realizadas por las autoras, organizaciones de la sociedad civil y otras personas dedicadas a la investigación y los programas de salud y derechos sexuales y reproductivos (DSDR) centrados en la salud sexual basada en el placer. Dichas iniciativas han señalado reiteradamente la necesidad de incluir de manera más integral el bienestar sexual dentro de los esfuerzos actuales en DSDR. La difusión de estos resultados contribuirá a orientar y fortalecer futuros programas y políticas de anticoncepción y planificación familiar, subrayando que las preocupaciones sobre la salud sexual deben ocupar un lugar central en la investigación y en las intervenciones de DSDR en el futuro.

Resultados

Se examinaron 16,660 resúmenes para identificar el contexto pertinente (anticoncepción), el diseño adecuado y la población de interés, y se seleccionaron 929 artículos para su evaluación en texto completo. Se excluyeron 348 artículos por no informar motivos de discontinuidad o cambio de método. De los 581 artículos restantes que sí reportaban alguna razón de discontinuidad o cambio, se excluyeron 482 por no incluir motivos relacionados con la vida sexual. En otras palabras, se excluyó el 83% (482/581) de los estudios que analizaban la discontinuidad porque no abordaban razones vinculadas con la vida sexual. Tras exclusiones adicionales basadas en el rango de edad y el diseño del estudio (k = 35), se retuvieron 64 artículos, que en conjunto representaron a más de 125,584 personas participantes de entre 12 y 54 años (véase la Figura 1 para el diagrama de flujo). Aunque algunos estudios —especialmente los de diseño

cualitativo— no informaron de manera transparente la discontinuidad “en necesidad”, se estimó que la muestra total de estudios incluidos representa al menos 59,926 personas que discontinuaron o cambiaron la anticoncepción, y un mínimo de 52,705 que lo hicieron estando en necesidad anticonceptiva. Las características de los estudios incluidos se presentan en la Tabla 1 (estudios cuantitativos) y en la Tabla 2 (estudios cualitativos).

Figura 1. Diagrama de flujo PRISMA que muestra el proceso de selección de estudios

	**Identificación de los estudios mediante bases de datos y registros**	
Identificación	Registros identificados n = 26,968, provenientes de:<br>Bases de datos (n = 26,928)<br>Búsquedas secundarias (n = 40)	Registros eliminados antes de la selección:<br>Registros duplicados eliminados (n = 10,308)
Selección	Registros examinados a nivel de resumen (n = 16,660)	Registros excluidos (n = 15,731)
	Artículos en texto completo evaluados para determinar su inclusión (n = 929)	Informes excluidos (n = 865), debido a:<br>No se informaron motivos de discontinuidad/cambio (n = 348)<br>No se informaron motivos relevantes a la vida sexual (n = 482)<br>Participantes sin necesidad anticonceptiva (n = 8)<br>Participantes fuera del rango etario (n = 11)<br>Diseño del estudio no pertinente (n = 13)<br>Solo condones (n = 3)
Inclusión	Estudios incluidos en la revisión<br>n = 64, de los cuales:<br>Cuantitativos n = 42<br>Cualitativos n = 22	

Resultados cuantitativos

Se realizó un metaanálisis de 25 estudios sobre discontinuidad, que representaron a 6,428 personas en necesidad que habían dejado el uso de métodos anticonceptivos, de las cuales 440 lo hicieron por motivos relacionados con la vida sexual. El modelo de efectos aleatorios mostró una prevalencia global de la

discontinuidad "en necesidad" por razones vinculadas con la vida sexual del 5% (IC del 95%: 0.03–0.08; I^2 = 94%; τ^2 = 1.31; Q = 381; p < 0.001) (véase la Figura 2).

Figura 2. Diagrama de bosque (forest plot) con los resultados de los análisis por subgrupos de acuerdo al contenido hormonal o no hormonal de los métodos anticonceptivos evaluados.
Notas: Los estudios se identifican mediante una cita abreviada. "Eventos" indica el número de casos de discontinuidad del método debido a motivos relacionados con la vida sexual reportados en cada estudio. "Total" se refiere al número total de casos de discontinuidad informados por personas en situación de necesidad. Por lo tanto, "Proporción" corresponde al porcentaje de interrupciones vinculadas con la vida sexual respecto del número total de interrupciones del uso registradas.

**Estudio**	**Eventos**	**Total**	**Proporción**	**IC 95 %**	**Peso**
**Tipo = No hormonal**					
Hofmeyr et al. (2019)	1	116	0.01	[0.00; 0.05]	3.2 %
Kriplani et al. (2019)	4	125	0.03	[0.01; 0.08]	4.7 %
Landolt et al. (2013)	1	2	0.50	[0.01; 0.99]	2.2 %
Saloranta et al. (2020)	0	53	0.00	[0.00; 0.07]	2.2 %
**Modelo de efectos aleatorios**		296	0.03	[0.01; 0.16]	12.2 %
Heterogeneidad: I^2 = 64 %, τ^2 = 1.7591, p = 0.04					
**Tipo = Ambos**					
Awoyesuku, Altraide y Amadi (2021)	3	276	0.01	[0.00; 0.03]	4.4 %
Brockmeyer et al. (2008)	1	15	0.07	[0.00; 0.32]	3.1 %
**Modelo de efectos aleatorios**		291	0.02	[0.00; 0.12]	7.5 %
Heterogeneidad: I^2 = 60 %, τ^2 = 1.0476, p = 0.11					
**Tipo = Hormonal**					
Armitage, Mitchell, Wigan y Smith (2012)	1	10	0.10	[0.00; 0.45]	3.0 %
Barreiros et al. (2006)	2	10	0.20	[0.03; 0.56]	2.9 %
Chaovisitsaree et al. (2005)	1	5	0.20	[0.01; 0.72]	3.0 %
Friedman (2015)	1	9	0.11	[0.00; 0.48]	3.0 %
Fruzzetti et al. (2016)	18	713	0.03	[0.02; 0.04]	5.3 %
Hajikazemi, Nikpour y Haghani (2004)	183	900	0.20	[0.18; 0.23]	5.5 %
Hofmeyr et al. (2019)	0	121	0.00	[0.00; 0.03]	2.2 %
Keogh et al. (2021)	0	156	0.00	[0.00; 0.02]	2.2 %
Lete et al. (2012)	141	913	0.15	[0.13; 0.18]	5.5 %
Littlejohn (2012)	15	1520	0.01	[0.01; 0.02]	5.3 %
Madden et al. (2012)	6	180	0.03	[0.01; 0.07]	4.9 %
Mrwebi et al. (2018)	2	170	0.01	[0.00; 0.04]	4.0 %
Regidor, Colli y Palacios (2021)	2	81	0.02	[0.00; 0.09]	4.0 %
Sabatini y Cagiano (2006)	21	58	0.36	[0.24; 0.50]	5.3 %
Saloranta et al. (2020)	17	376	0.05	[0.03; 0.07]	5.3 %
Schafer, Osborne, Davis y Westhoff (2006)	8	160	0.05	[0.02; 0.10]	4.9 %
Ssebatta, Kaye y Mbalinda (2023)	7	138	0.05	[0.02; 0.10]	5.0 %
Wjocik et al. (2022)	6	54	0.11	[0.04; 0.23]	4.9 %
**Modelo de efectos aleatorios**		5433	0.06	[0.03; 0.10]	75.3 %
Heterogeneidad: I^2 = 94 %, τ^2 = 1.1577, p < 0.01					
**Tipo = No especificado**					
Park, Nguyen y Ngo (2011)	6	408	0.01	[0.01; 0.03]	4.9 %
**Modelo de efectos aleatorios global**		6428	0.05	[0.03; 0.08]	100 %
Heterogeneidad: I^2 = 94 %, τ^2 = 1.3130, p < 0.01					
Prueba de diferencias entre subgrupos: χ^2_3 = 8.21, gl = 3 (p = 0.04)					

En cuanto al sesgo de publicación, la inspección del gráfico de embudo (Figura suplementaria 1) y la prueba de Egger ($p < 0.001$) evidenciaron cierta asimetría. Tras aplicar el procedimiento de poda y relleno (trim-and-fill) de Duval y Tweedie, se imputaron 11 estudios adicionales. Al recalcular la prevalencia global, el valor ajustado aumentó al 14% (IC del 95%: 0.09–0.22) (véanse las Figuras suplementarias 1B y 2 para los gráficos en embudo y de bosque actualizados). Este resultado debe interpretarse con cautela, ya que en la mayoría de los estudios los motivos de discontinuidad se presentan de manera descriptiva y no como el resultado inferencial principal (por ejemplo, la aceptabilidad del método), que sería el tipo de análisis donde normalmente podría esperarse un sesgo de publicación.

Metaanálisis y meta-regresiones por subgrupos

También fue posible realizar varios de los análisis por subgrupos pre-registrados en el protocolo, en particular aquellos que examinaron las diferencias entre métodos anticonceptivos según su contenido hormonal y su tipo específico, así como posibles diferencias en los motivos de discontinuidad de acuerdo con el marco teórico de Higgins y Smith[20] y con su nivel (individual o relacional). Cabe señalar que algunos análisis de subgrupos incluyeron pocas observaciones o se basaron en datos limitados, y que los distintos subgrupos pueden sustentarse en evidencia de calidad o cantidad variable (número de estudios), por lo que sus resultados deben interpretarse con precaución.[29] Para clarificar cada análisis por subgrupo, se realizaron una serie de meta-regresiones univariadas y, adicionalmente, una meta-regresión multivariada que consideró tanto el contenido hormonal como el tipo de método anticonceptivo (véase el Material suplementario 2 para los resultados completos).

En la Figura 2 se presenta un gráfico de bosque (forest plot) con la categorización por subgrupos, según si cada estudio analizó únicamente métodos no hormonales, únicamente métodos hormonales, ambos tipos (sin desagregación posible) o métodos no especificados. Esta figura incluye los mismos datos utilizados en el metaanálisis principal (25 estudios; 6,428 personas que discontinuaron, de las cuales 440 lo hicieron por motivos relacionados con la vida sexual). Los cuatro puntos de datos correspondientes a los métodos no hormonales se basan exclusivamente en el uso del DIU de cobre. En cambio, los estudios sobre métodos hormonales abarcan una gama más amplia de opciones, como el anillo vaginal, el implante, la píldora, la inyección y el sistema intrauterino con levonorgestrel (LNG-IUS). La estimación global de prevalencia de la discontinuidad de métodos no hormonales por motivos vinculados con la vida sexual fue del 3% (IC del 95%: 1%–16%), mientras que para los métodos hormonales fue del 6% (IC del 95%: 3%–10%). Aunque la prueba ji-cuadrado global indicó diferencias estadísticamente significativas entre subgrupos (p = 0.04), el análisis por pares entre métodos hormonales y no hormonales no alcanzó significación estadística al nivel alfa estándar (0.05) en la meta-regresión de seguimiento (véase Material suplementario 2).

La Figura 3 muestra un gráfico de bosque (forest plot) con la categorización por subgrupos según el tipo específico de método anticonceptivo. Este análisis incluyó un total de 6,419 personas en necesidad anticonceptiva que discontinuaron su uso, de las cuales 440 lo hicieron por motivos relacionados con la vida sexual. La prueba ji-cuadrado global reveló diferencias significativas entre los grupos (p = 0.001). Las estimaciones de prevalencia fueron las siguientes: 16% para el anillo vaginal [IC del 95%: 0.05–0.40], 7%

para personas usuarias del sistema intrauterino con levonorgestrel (LNG-IUS) [IC del 95%: 0.05–0.11], 3% para el DIU de cobre no hormonal [IC del 95%: 0.01–0.16], 4% para los implantes [IC del 95%: 0.02–0.06], 4% para las inyecciones [IC del 95%: 0.00–0.70] y 3% para la píldora anticonceptiva [IC del 95%: 0.02–0.04]. La meta-regresión posterior no mostró evidencia de diferencias significativas por pares en comparación con el DIU de cobre no hormonal.

Figura 3. Diagrama de bosque (forest plot) que presenta los resultados de los análisis por subgrupos según el tipo específico de método anticonceptivo evaluado. Los estudios se identifican mediante una cita abreviada.
Notas: Los estudios se identifican mediante una cita abreviada. "Eventos" indica el número de casos de discontinuidad del método debido a motivos relacionados con la vida sexual reportados en cada estudio. "Total" se refiere al número total de casos de discontinuidad informados por personas en situación de necesidad. Por lo tanto, "Proporción" corresponde al porcentaje de interrupciones vinculadas con la vida sexual respecto del número total de interrupciones del uso registradas.

**Estudio**	**Eventos**	**Total**	**Proporción**	**IC 95 %**	**Peso**
**Tipo = Sistema intrauterino con levonorgestrel (LNG-IUS)**					
Armitage, Mitchell, Wigan y Smith (2012)	1	10	0.10	[0.00; 0.45]	2.9 %
Saloranta et al. (2020)	12	203	0.06	[0.03; 0.10]	5.0 %
Wjocik et al. (2022)	6	54	0.11	[0.04; 0.23]	4.7 %
**Modelo de efectos aleatorios**		**267**	**0.07**	**[0.05; 0.11]**	**12.5 %**
Heterogeneidad: $I^2 = 0$ %, $\tau^2 = 0$, $p = 0.40$					
**Tipo = Varios métodos**					
Awoyesuku, Altraide y Amadi (2021)	3	276	0.01	[0.00; 0.03]	4.2 %
Brockmeyer et al. (2008)	1	15	0.07	[0.00; 0.32]	2.9 %
Friedman (2015)	1	9	0.11	[0.00; 0.48]	2.9 %
Keogh et al. (2021)	0	156	0.00	[0.00; 0.02]	2.1 %
Lete et al. (2012)	141	913	0.15	[0.13; 0.18]	5.3 %
Littlejohn (2012)	15	1520	0.01	[0.01; 0.02]	5.1 %
Sabatini y Cagiano (2006)	21	58	0.36	[0.24; 0.50]	5.0 %
**Modelo de efectos aleatorios**		**2947**	**0.05**	**[0.01; 0.16]**	**27.6 %**
Heterogeneidad: $I^2 = 96$ %, $\tau^2 = 2.7909$, $p < 0.01$					
**Tipo = Anillo vaginal**					
Barreiros et al. (2006)	2	10	0.20	[0.03; 0.56]	3.6 %
Schafer, Osborne, Davis y Westhoff (2006)	1	9	0.11	[0.00; 0.48]	2.9 %
**Modelo de efectos aleatorios**		**19**	**0.16**	**[0.05; 0.41]**	**6.5 %**
Heterogeneidad: $I^2 = 0$ %, $\tau^2 = 0$, $p = 0.60$					
**Tipo = Implante**					
Chaovisitsaree et al. (2005)	1	5	0.20	[0.01; 0.72]	2.7 %
Madden et al. (2012)	6	180	0.03	[0.01; 0.07]	4.7 %
Mrwebi et al. (2018)	2	170	0.01	[0.00; 0.04]	3.8 %
Saloranta et al. (2020)	5	174	0.03	[0.01; 0.07]	4.6 %
Ssebatta, Kaye y Mbalinda (2021)	7	138	0.05	[0.02; 0.10]	4.8 %
**Modelo de efectos aleatorios**		**667**	**0.04**	**[0.02; 0.06]**	**20.7 %**
Heterogeneidad: $I^2 = 0$ %, $\tau^2 = 0.1776$, $p = 0.16$					
**Tipo = Píldora anticonceptiva**					
Fruzzetti et al. (2016)	18	713	0.03	[0.02; 0.04]	5.1 %
Regidor, Colli y Palacios (2021)	2	81	0.02	[0.00; 0.09]	3.8 %

**Modelo de efectos aleatorios**		**794**	**0.03**	**[0.02; 0.04]**	**8.9 %**
Heterogeneidad: I^2 = 0 %, τ^2 = 0, p = 0.98					
**Tipo = Inyección anticonceptiva**					
Hajikazemi, Nikpour y Haghani (2004)	183	900	0.20	[0.18; 0.23]	5.3 %
Hofmeyr et al. (2019)	0	121	0.00	[0.00; 0.03]	2.1 %
**Modelo de efectos aleatorios**		**1021**	**0.04**	**[0.00; 0.70]**	**7.4 %**
Heterogeneidad: I^2 = 88 %, τ^2 = 7.5105, p < 0.01					
**Tipo = DIU de cobre (Copper-IUD)**					
Hofmeyr et al. (2019)	1	116	0.01	[0.00; 0.05]	3.0 %
Kriplani et al. (2019)	4	125	0.03	[0.01; 0.08]	4.5 %
Landolt et al. (2013)	1	2	0.50	[0.01; 0.99]	2.1 %
Saloranta et al. (2020)	0	53	0.00	[0.00; 0.07]	2.1 %
**Modelo de efectos aleatorios**		**296**	**0.03**	**[0.01; 0.16]**	**11.7 %**
Heterogeneidad: I^2 = 64 %, τ^2 = 1.7591, p = 0.04					
**Tipo = No especificado**					
Park, Nguyen y Ngo (2011)	6	408	0.01	[0.01; 0.03]	4.7 %
**Modelo de efectos aleatorios global**		**6419**	**0.05**	**[0.03; 0.08]**	**100 %**

Heterogeneidad global: I^2 = 93 %, τ^2 = 1.3057, p < 0.01
Prueba de diferencias entre subgrupos: χ^2_7 = 24.24; gl = 7; p < 0.01

Se presentan gráficos de bosque (forest plots) para los análisis por subgrupos, tanto por categoría (véase la Figura suplementaria 3) como por nivel del motivo (véase la Figura suplementaria 4), de acuerdo con el marco teórico de Higgins y Smith.[20] Ni las pruebas ji-cuadrado ni las meta-regresiones posteriores mostraron evidencia de diferencias estadísticamente significativas entre subgrupos, ya sea por categoría o por nivel del motivo. En cuanto a la categoría de motivos, se desagregaron las razones específicas de discontinuidad en: preocupaciones sobre el funcionamiento sexual, especialmente la libido (prevalencia estimada: 4% [IC del 95%: 0.02–0.09]); preocupaciones sobre el placer o funcionamiento de la pareja (6% [IC del 95%: 0.02–0.14]); y preferencias o estética sexual (6% [IC del 95%: 0.01–0.30]). En cuanto al nivel de los motivos, estos se desagregaron en: preocupaciones que afectan principalmente a la relación (prevalencia estimada: 6% [IC del 95%: 0.02–0.14]); o preocupaciones que afectan principalmente a la persona misma (4% [IC del 95%: 0.02–0.08]).

Riesgo de sesgo

En cuanto a la evaluación de la calidad, se observó que la calidad metodológica de los estudios fue heterogénea (véase el Material suplementario 3). En los estudios cuantitativos, los ensayos aleatorizados presentaron en general algunas limitaciones, principalmente grupos no equilibrados y dificultades en la asignación aleatoria. La mayoría de los estudios de cohorte mostraron una calidad global aceptable; las limitaciones más comunes incluyeron bajas tasas de respuesta y abandono, poca información sobre las estrategias de reclutamiento y posibles conflictos de interés, como el financiamiento proveniente de

compañías farmacéuticas productoras de anticonceptivos. En contraste, la mayoría de los estudios cualitativos fueron de buena calidad. Los problemas más frecuentes se relacionaron con la falta de reflexividad y, en algunos casos, detalle insuficiente sobre el enfoque analítico utilizado. Para evaluar más a fondo el posible impacto del riesgo de sesgo, se realizó un metaanálisis por subgrupos, agrupando los estudios según su puntuación global (véase la Figura suplementaria 5). La prueba ji-cuadrado global no fue significativa (p = 0.85), lo que se corroboró con los resultados también no significativos de una meta-regresión univariada sobre el riesgo de sesgo (véase el Material suplementario 2). En conjunto, no se encontró evidencia de diferencias significativas en el tamaño del efecto entre los estudios según su puntuación global de riesgo de sesgo.

Síntesis narrativa

La población incluida está compuesta casi exclusivamente por mujeres que utilizan métodos anticonceptivos, de entre 12 y 54 años, encuestadas en entornos clínicos o de salud pública, que reportaron la discontinuidad o cambio de método. Los estudios se llevaron a cabo en una amplia variedad de contextos a nivel mundial, y se reconoce que mencionar preocupaciones relacionadas con la vida sexual puede resultar un tema sensible —e incluso tabú— en algunos entornos. En dos artículos, los motivos vinculados con la vida sexual para la discontinuidad fueron reportados únicamente por parejas masculinas.[30,31] En otros tres artículos se presentaron motivos referidos por las propias mujeres, además de los motivos señalados por personal de salud[32] y por parejas masculinas.[33,34] Aunque habíamos pre-registrado el interés en analizar otras características que considerábamos potencialmente relevantes para comprender la relación entre las preocupaciones sobre la vida sexual y la discontinuidad (por ejemplo, orientación sexual, etnicidad, nivel socioeconómico y estado civil/de relación), estos datos no se reportaron en la mayoría de los artículos, por lo que no fue posible reflejar las experiencias de las personas con estas características. Desde el punto de vista cualitativo, se observó que factores físicos o clínicos no contemplados en el pre-registro, como el peso y el índice de masa corporal (IMC), tendieron a reportarse con mayor frecuencia.

La gran mayoría de los artículos incluidos, k = 63 (aproximadamente 98% del total), analizaron la discontinuidad de métodos anticonceptivos. Se observó que el cambio de método ha sido un tema comparativamente menos estudiado: solo un artículo (~1%) examinó exclusivamente el cambio, y otros tres (~5%) consideraron tanto el cambio como la discontinuidad. En cuatro de los artículos centrados en la discontinuidad, el cambio o el deseo de cambiar de método se incluyeron como motivos asociados a la discontinuidad. Disponemos de datos sobre métodos no hormonales en 21 estudios y sobre métodos hormonales en 56 (algunos de los cuales también incluían métodos no hormonales); en tres estudios adicionales, el método utilizado fue un dispositivo intrauterino (DIU), aunque no se especificó si era de cobre u hormonal.[35] Esta revisión abarca diversos métodos anticonceptivos específicos, como los DIU de cobre y hormonales, el diafragma, el capuchón cervical, la píldora anticonceptiva, la inyección, el implante y el anillo vaginal. En conjunto, observamos que la discontinuidad por motivos vinculados con la vida sexual varía

desde tan bajo como 0%[36] hasta 36.2%[37] en el caso de métodos hormonales, y desde 0.1%[36] hasta 50%[38] para métodos no hormonales (nota: este último valor proviene de una muestra de solo dos personas que abandonaron el método estando en necesidad).

La discontinuidad y el cambio de método tienden a ser los resultados principales en la mayoría de los estudios incluidos. Sin embargo, es importante destacar que los motivos de discontinuidad o cambio suelen reportarse de forma predominantemente descriptiva y rara vez constituyen el enfoque inferencial central de los artículos (que suelen centrarse, por ejemplo, en predecir la aceptabilidad del método o analizar determinantes personales o sociales asociados con la discontinuidad). Teniendo en cuenta esto, y dada la diversidad de diseños de investigación, el reporte y la medición de los motivos resultan variables (véanse las Tablas suplementarias 1 y 2). En algunos estudios, las personas participantes solo podían mencionar un motivo para la discontinuidad o el cambio, generalmente descrito como el "principal" o el "más importante."[40–42] En otros casos (menos frecuentes en los diseños cuantitativos y más habituales en los cualitativos), las personas participantes podían señalar múltiples motivos que influyeron en su proceso de toma de decisiones, así como contextualizarlos o compararlos según su relevancia relativa para distintos aspectos de su vida.[42,43] En algunos estudios no se especificó con claridad cuántos motivos podían reportarse.[45,46] Esto dificultó obtener una comprensión sistemática de las formas más comunes de indagar los motivos. En los estudios cualitativos, pocos emplearon entrevistas semiestructuradas y, en general, la mayoría aceptó respuestas libres de las personas participantes (especialmente en entrevistas en profundidad y grupos focales), las cuales se organizaron temáticamente y, en algunos casos, se citaron parcial o totalmente. En contraste, en la mayoría de los estudios cuantitativos no fue posible determinar con claridad si las personas participantes recibieron categorías predefinidas para seleccionar sus motivos o si proporcionaron respuestas libres que posteriormente fueron clasificadas. Como patrón común, la mayoría de los estudios solicitó motivos generales de discontinuidad, y solo una minoría tuvo como objetivo principal examinar un motivo específico (por ejemplo, el sangrado[47]). Sorprendentemente, solo se identificaron dos estudios en los que las personas participantes fueron preguntadas de manera directa y específica sobre cómo la anticoncepción afectaba su vida sexual y, a su vez, cómo sus preocupaciones sobre la vida sexual se asociaban con la discontinuidad (libido[48] y deseo y satisfacción sexual[38]).

En cuanto a la forma de reporte, los equipos de investigación con frecuencia agruparon los motivos en categorías amplias que no se prestaban a la desagregación. Por ejemplo, algunos estudios informaron la discontinuidad bajo etiquetas generales como "efectos secundarios" u "otros motivos", que podían representar hasta el 30% del total de razones registradas.[48] Al analizar específicamente las preocupaciones relacionadas con la vida sexual, los motivos más comunes a nivel individual se vincularon con el funcionamiento sexual, especialmente con la disminución o pérdida de la libido. En el nivel relacional, los motivos más frecuentes se asociaron con preocupaciones sobre el placer o el funcionamiento sexual de la pareja, que en la mayoría de los casos se relacionaba con la percepción de la pareja de sentir el dispositivo anticonceptivo (el DIU o anillo vaginal) durante la actividad sexual.

Además, en la mayoría de los artículos incluidos, los motivos informados por las personas que discontinuaron o cambiaron su método anticonceptivo se agruparon sin considerar el tipo de método

utilizado, lo que impidió vincular de manera clara los motivos específicos con métodos específicos (véanse las Tablas suplementarias 1 y 2). Por ejemplo, aunque se incluyeron 19 estudios que analizaron el uso del DIU de cobre —el método no hormonal más común identificado—, solo fue posible relacionar motivos específicos con la discontinuidad del DIU de cobre en menos de un tercio de los casos, es decir, en solo seis estudios: incomodidad durante la actividad sexual (k = 2), percepción del DIU por parte de la pareja (k = 2), rumores de que el dispositivo interfiere con la actividad sexual(k = 1) y problemas relacionados con la libido (k = 1). De manera similar, aunque contamos con datos sobre métodos anticonceptivos hormonales provenientes de 56 estudios, únicamente en 30 fue posible vincular motivos específicos con métodos concretos. Por ejemplo, la discontinuidad del implante se examinó en 31 estudios, pero solo en 11 se pudo atribuir la decisión a motivos específicos, siendo el más común la disminución o pérdida de la libido (k = 7). La discontinuidad del anillo vaginal se analizó en 12 artículos, de los cuales 8 permitieron asociar motivos específicos con este método; en la mayoría de los casos, el anillo fue abandonado porque la pareja masculina lo percibía durante la actividad sexual (k = 6).

Discusión

Principales hallazgos

En esta sección se presentan y analizan cuatro hallazgos clave: (1) los motivos relacionados con la vida sexual constituyen causas frecuentes e importantes de discontinuidad del uso de métodos anticonceptivos; (2) su prevalencia resulta comparable a la de otros motivos de discontinuidad, como la accesibilidad o el costo; (3) las razones relacionadas con la vida sexual son objeto de investigación con poca frecuencia; y (4) cuando se abordan, surgen diversas dificultades metodológicas para su medición.

Presentamos evidencia de que los motivos vinculados con la vida sexual constituyen un grupo importante y visible de razones para la discontinuidad del uso de métodos anticonceptivos. Según nuestro metaanálisis, estos motivos tienen una prevalencia promedio global del 5% (IC del 95%: 0.03, 0.08; I^2 = 94%, τ^2 = 1.31; Q = 381, p < 0.001). Una frecuencia de una de cada veinte personas se considera “común”, de acuerdo con distintos organismos médicos, como las directrices de la Comisión Europea[49] o el Servicio Nacional de Salud del Reino Unido (NHS).[50] Aunque probablemente un número mayor de mujeres haya experimentado dificultades relacionadas con su vida sexual que aquellas que efectivamente interrumpieron el uso de métodos anticonceptivos por ese motivo, nuestros hallazgos muestran que el impacto de un método sobre la vida sexual de quien lo usa merece la misma atención rigurosa que otras razones de discontinuidad con prevalencias similares.

Por ejemplo, al comparar las tasas globales de discontinuidad del uso de métodos anticonceptivos en 36 países de ingresos bajos y medianos —según las Encuestas Demográficas y de Salud (DHS)—, una tasa del 5% resulta similar a la discontinuidad atribuida al costo (3,5%) o a la falta de acceso (4,7%).[19] Se han realizado inversiones sustanciales —financieras, de tiempo, logísticas y en investigación— para abordar estos problemas, por ejemplo, la entrega de cupones para obtener anticonceptivos gratuitos o de bajo costo,

que han demostrado aumentar su uso en algunos contextos.[51,52] Asimismo, la provisión de servicios gratuitos puede incrementar la adopción, especialmente en el caso de métodos reversibles de larga duración, que suelen ser más costosos.[53] En cuanto al acceso, las inversiones en servicios móviles de alcance comunitario[54], el fortalecimiento del acceso mediante agentes comunitarios de salud,[55] y la redistribución de tareas,[56] han mostrado mejoras en el uso de métodos anticonceptivos. Dado que nuestros hallazgos muestran que el 5% de las personas abandona su método por motivos relacionados con la vida sexual, deberían impulsarse esfuerzos e inversiones equivalentes para abordar la aceptabilidad sexual de la anticoncepción.

Para abordar la discontinuidad del uso de métodos anticonceptivos por motivos relacionados con la vida sexual, la comunidad de planificación familiar y anticoncepción debe invertir también en una medición más precisa, consistente y directa de las preocupaciones sobre la vida sexualy de la aceptabilidad sexual. Nuestra revisión mostró que los efectos sobre la vida sexual rara vez se miden y evalúan en la literatura sobre discontinuidad: se excluyó el 83% (482 de 581) de los artículos que analizaban la discontinuidad del método *sin* explorar motivos vinculados con la vida sexual. Además, en nuestra muestra solo dos estudios indagaron explícitamente —y como objetivo principal— el impacto de la anticoncepción en la vida sexual, específicamente en la libido[47] y la satisfacción sexual.[37] Incluir las consideraciones sobre la vida sexual en el estudio de la toma de decisiones anticonceptivas es un paso fundamental y alcanzable para la investigación, especialmente considerando que una de las principales motivaciones para usar métodos anticonceptivos es la posibilidad de tener relaciones sexuales seguras.[57] Actualmente, los efectos sobre la vida sexual siguen siendo un aspecto poco explorado en el campo de la anticoncepción.

Es fundamental que la mayor inclusión de los impactos sobre la vida sexual vaya acompañada de estándares más rigurosos y uniformes en la forma de reportarlos. Nuestra revisión enfrentó dificultades metodológicas recurrentes, especialmente con artículos que agruparon múltiples razones bajo categorías amplias o poco transparentes, lo que impidió su análisis diferenciado. Esto coincide con estudios previos en los que los motivos más citados para la discontinuidad del uso de métodos modernos se informaron en categorías generales, como "efectos secundarios y problemas de salud" (40,2 %; 19), sin especificar claramente qué incluían. De hecho, uno de los artículos revisados mencionó "otros motivos" de discontinuidad en más del 30% de los casos.[48] En algunos estudios, es posible que las propias personas prefirieran mencionar los efectos secundarios como motivo principal sin detallar más, mientras que en otros las y los investigadores agruparon distintas razones dentro de categorías genéricas. Nuestros hallazgos coinciden con una revisión crítica anterior que mostró cómo categorías como "otros motivos" pueden reunir causas muy distintas, que incluyen impactos sobre la vida sexual, cambios de preferencia o incluso alergias.[21] De forma similar, se ha observado que, aunque la categoría "efectos secundarios" suele referirse a efectos médicamente indeseables (principalmente el sangrado), la naturaleza y relevancia de las razones individuales no siempre quedan claras. De hecho, diversos efectos como cefaleas, hinchazón, acné, sangrado o sequedad vaginal pueden influir en la vida sexual de las personas , pero ni estos efectos específicos ni sus posibles consecuencias secundarias se miden o describen con suficiente transparencia en la literatura primaria. La información sobre el funcionamiento y la aceptabilidad sexual durante el uso de métodos anticonceptivos sigue siendo muy limitada, y la investigación futura debería ser más proactiva y sistemática

al explorar estas perspectivas de las personas . Recomendamos una medición más focalizada y consistente de los impactos en la vida sexual, así como una presentación más transparente y diferenciada de las razones —distinguiendo claramente entre los casos en que las personas agrupan sus motivos y aquellos en que lo hacen las y los investigadores—, además de una mayor apertura para captar matices. Esto puede lograrse preguntando directamente a las personas si los impactos relacionados con su vida sexual influyeron en su decisión de cambiar o discontinuar el método, permitiéndoles explicar sus razones y seleccionar más de una opción cuando corresponda, en lugar de limitarse a una lista cerrada.

Fortalezas, limitaciones y perspectivas futuras

En cuanto a las fortalezas, hasta donde tenemos conocimiento, esta es la primera revisión sistemática con metaanálisis que busca comprender los tipos y la prevalencia de las preocupaciones vinculadas con la vida sexual como razones para la discontinuidad o el cambio de métodos anticonceptivos. Investigaciones previas han señalado que las intervenciones en salud y derechos sexuales y reproductivos que incorporan la dimensión del placer sexual suelen pasar por alto a las mujeres en edad reproductiva.[26] esperamos que este trabajo contribuya a visibilizar la influencia que los distintos aspectos de la vida sexual ejercen sobre las decisiones relacionadas con el uso métodos de anticonceptivos.

Otra fortaleza de esta revisión es la inclusión de datos diversos, de carácter global, que abarcan una amplia gama de métodos anticonceptivos, así como la posibilidad de analizarlos con detalle —aunque con cautela— en los análisis por subgrupos. No se encontró evidencia de diferencias en la prevalencia de motivos relacionados con la vida sexual entre métodos hormonales y no hormonales, ni entre métodos específicos o categorías distintas de motivos. Aunque las razones individuales pueden variar, es posible que la frecuencia general de las preocupaciones vinculadas con la vida sexual que motivan la discontinuidad del uso de métodos anticonceptivos sea similar entre los distintos métodos. Algunas de nuestras estimaciones —en especial las referidas a métodos concretos— se basan en pocos estudios, por lo que futuras investigaciones, o una presentación más desagregada de los datos disponibles, podrían ayudar a aclarar mejor estas posibles diferencias.

En cuanto a las limitaciones, nuestros datos y conclusiones están condicionados por ciertas tendencias persistentes observadas en la literatura existente. Es posible que hayamos pasado por alto algunos estudios que midieron preocupaciones vinculadas con la vida sexual pero no los reportaron con transparencia; por ejemplo, cuando las razones se agrupan bajo categorías amplias como “efectos secundarios”, “otros motivos” u “oposición de la pareja o del esposo”, como se mencionó antes. Investigaciones previas han señalado que la categoría “otros motivos” puede incluir, en algunos casos, efectos vinculados con la disminución de la libido.[21,58] Asimismo, en nuestro análisis por subgrupos sobre métodos anticonceptivos específicos, solo fue posible asociar las razones de discontinuidad con métodos concretos en pocos casos, ya que muchos estudios presentan cifras globales sin distinguir el tipo de método, lo que limita la precisión de las interpretaciones. Para superar estas limitaciones y obtener conclusiones más sólidas, recomendamos evaluar directamente

las preocupaciones relacionadas con la vida sexual en investigaciones futuras y darles un papel más central dentro del estudio del uso y la elección de anticonceptivos.

Otra limitación de este trabajo es el uso del embarazo no intencional y de la necesidad insatisfecha como indicadores en la revisión. Reconocemos los debates actuales sobre la necesidad de contar con medidas más precisas, que reflejen mejor los valores y preferencias de las personas en torno a la fertilidad y al uso de métodos anticonceptivos.[59,60] Se eligió el embarazo no intencional como un término amplio que abarca tanto los embarazos inoportunos como los no deseados, en coherencia con la literatura analizada. Nuestra definición más restringida de personas "con necesidad" (de anticoncepción) incluyó a quienes no deseaban quedar embarazadas. Un enfoque alternativo sería definir como "con necesidad" a quienes no utilizan anticonceptivos pero quisieran hacerlo. Esta definición resulta importante porque pone el énfasis en la autonomía y la toma de decisiones de las personas . Para los fines de esta revisión sistemática y metaanálisis, adoptamos un enfoque pragmático y definimos "con necesidad" conforme a los marcos comúnmente utilizados, como los de las Encuestas Demográficas y de Salud. Esto permitió incluir estudios con una definición ampliamente compartida, aunque reconocemos que las alternativas, si bien más inclusivas, habrían sido más difíciles de aplicar de manera sistemática. En este sentido, consideramos que las estimaciones presentadas son sólidas, aunque conservadoras. A medida que la comunidad dedicada a la medición en planificación familiar continúe revisando y actualizando su terminología, la investigación futura debería considerar definiciones más recientes y explícitamente basadas en los derechos humanos, especialmente al analizar intervenciones y programas sobre planificación familiar y uso de métodos anticonceptivos.

También es probable que estemos subestimando la prevalencia global de las preocupaciones relacionadas con la vida sexual en el contexto de la discontinuidad del uso de métodos anticonceptivos. En primer lugar, esto puede deberse a que en gran parte de la literatura analizada se solicita a las personas que indiquen un solo motivo para discontinuar su método, cuando en realidad las personas suelen considerar varios factores en su toma de decisiones.[20] Además, es posible que las personas tiendan a ajustarse a normas socioculturales[31,61] y que, al tener que elegir un único motivo, reporten menos las razones vinculadas con la vida sexual y prefieran mencionar motivos más "socialmente aceptables", debido a la dificultad o incluso al tabú que todavía existe en algunos contextos para hablar abiertamente de sexualidad.[62] En este trabajo adoptamos una definición estricta de lo que constituye un motivo relacionado con la vida sexual, con el fin de estimar una prevalencia mínima clara y plausible. Sin embargo, es posible que algunos motivos relevantes no hayan sido recogidos por nuestra revisión. Por ejemplo, excluimos razones como la "oposición de la pareja" cuando no se especificaba su fundamento, aunque en algunos casos podría reflejar situaciones en las que la pareja percibía los hilos del DIU durante las relaciones sexuales. Del mismo modo, categorías más amplias como "inconveniencia del método", "dolor" o "molestia" podrían incluir efectos vinculados con la vida sexual, pero las excluimos a menos que las personas los mencionaran de manera explícita. También excluimos los informes de discontinuidad relacionados con sangrado (o sequedad, dolor pélvico o prurito) salvo que se indicara directamente que afectaban la vida sexual, aunque reconocemos que los cambios

asociados con el sangrado pueden influir en la vida diaria —incluida la sexual— mediante mecanismos a menudo superpuestos.[63,64]

La mayoría de los estudios incluidos analizan la discontinuidad del uso de métodos anticonceptivos, por lo que comparativamente existe una brecha de conocimiento respecto a los cambios de método. No encontramos un número suficiente de trabajos que exploraran las implicaciones sobre la vida sexual en el contexto de estos cambios como para realizar un metaanálisis, por lo que este sigue siendo un tema poco estudiado. Es fundamental comprender por qué las personas deciden cambiar de una opción a otra y ofrecer una consejería adecuada, que contemple diferentes alternativas según sus necesidades. Comprender las razones por las cuales alguien interrumpe el uso de un método resulta clave para brindar una consejería más centrada en la persona usuaria, mejorar el diseño y la adaptación de las opciones existentes y orientar hacia elecciones que no reproduzcan los mismos problemas. En este sentido, cada cambio implica haber abandonado una forma previa de anticoncepción, por lo que sigue siendo importante comprender las razones detrás de esa decisión.

También encontramos más datos sobre métodos hormonales que sobre los no hormonales, aspecto que merece mayor atención. El DIU de cobre fue la opción no hormonal más estudiada, por lo que existen vacíos de información en torno a otras alternativas, como el diafragma o los capuchones cervicales. Además, analizar los cambios de uso y una gama más amplia de métodos hormonales permitiría comprender mejor los distintos factores que influyen en las decisiones de anticoncepción y sus resultados. Es importante reconocer, sin embargo, que las razones para interrumpir el uso pueden variar entre personas y circunstancias. En esta revisión nos centramos únicamente en los motivos reportados y no abordamos otros factores explicativos —como los económicos o sociales—, que siguen siendo una parte esencial para entender los patrones de uso y elección de anticonceptivos.

Por último, la mayoría de los estudios que abordaron razones relacionadas con la vida sexual suelen analizar solo un aspecto, generalmente el deseo o la libido, aunque distintos componentes de la sexualidad pueden tener una relevancia diferente en las decisiones sobre anticoncepción. En este sentido, considerar un espectro más amplio de factores sexuales podría aportar valor a las investigaciones futuras. En cuanto a los resultados por subgrupos según la categoría y el nivel de las razones de discontinuidad, los estudios se agruparon conforme a un modelo existente de aceptabilidad sexual, desarrollado y validado mediante una revisión narrativa.[20] Este enfoque difiere de los métodos cuantitativos, en los que los constructos latentes se generan a partir de técnicas basadas en datos —como el análisis de componentes principales— que buscan reducir la dimensionalidad. Por ello, es posible que algunas categorías (por ejemplo, placer y búsqueda de placer o funcionamiento sexual, especialmente la libido) y niveles (individual y relacional) se superpongan, lo que impidió distinguirlas con suficiente claridad en los análisis.

Implicaciones para la investigación y programas

Los hallazgos de este estudio tienen implicaciones tanto para la investigación como para los programas orientados a fortalecer la salud y los derechos sexuales y reproductivos. Al destacar la importancia de la interacción de cada método con la vida sexual de quienes lo utilizan, este trabajo contribuye a un enfoque más integral y basado en los derechos, en línea con las brechas señaladas por la Comisión *Lancet* de 2018 sobre DSDR.[22] En el Recuadro 1 presentamos un resumen de las principales implicaciones. Creemos que existen oportunidades concretas para mejorar los enfoques de investigación, sobre todo en la medición de las razones globales para interrumpir o cambiar métodos, y en particular las motivaciones vinculadas con la vida sexual. Los estudios deberían evaluar y registrar de manera sistemática estas razones, permitiendo que las personas informen más de una y las expresen con amplitud, matices y sin juicios de valor. Este esfuerzo debe complementarse con mejores prácticas de reporte, que incluyan una presentación clara y eviten agrupar causas muy distintas en categorías amplias o poco informativas (por ejemplo, “otros motivos”). Dada la escasez de estudios que aborden este tema, resulta necesario medir de forma específica las razones relacionadas con la vida sexual, preguntando directamente a las personas sobre los posibles impactos y ofreciéndoles la oportunidad de explicar cómo se entrelazan sus motivos con su vida sexual.

Recuadro 1. Resumen de las implicaciones para la investigación y programáticas

Implicaciones para la investigación	1) Es necesario mejorar la medición general de los cambios en el uso de métodos anticonceptivos, centrando la atención en la autonomía y las perspectivas de las personas , incluidas sus razones para la discontinuidad o el cambio de método. Esto debería incluir:<br>• Incorporar sistemáticamente la evaluación de las razones para la discontinuidad.<br>• Preguntar a las personas por sus motivos de forma integral, reconociendo un amplio y diverso espectro de posibles razones.<br>• Permitir que informen más de una razón, entendiendo que pueden tener diferente importancia relativa (por ejemplo, principal o secundaria).<br>• Solicitar las razones con matices, sensibilidad contextual y sin juicios de valor, reconociendo que algunas pueden ser más o menos socialmente aceptables, y que pueden superponerse.<br>• Elevar los estándares de reporte, asegurando una descripción clara de las razones y evitando agrupar razones sustancialmente diferentes en categorías generales (por ejemplo, “otros motivos” o “efectos secundarios”).<br>2) Se necesita mejorar la mejor medición de las razones vinculadas con la vida sexual, lo que debería incluir:<br>• Incorporar de forma sistemática y como estándar la evaluación de los impactos en la vida sexual y la aceptabilidad sexual de los métodos anticonceptivos, por ejemplo, preguntando directamente a las personas .<br>• Considerar cómo las preocupaciones vinculadas con la vida sexual pueden operar como razones primarias o secundarias, o estar incorporadas o derivarse de otros motivos (por ejemplo, sangrado, sequedad).
Implicaciones Programáticas	1) La aceptabilidad sexual debe incorporarse en la consejería y en la programación de planificación familiar y anticoncepción como parte de un enfoque basado en los derechos humanos. A modo de ejemplo, esto podría incluir:<br>• Mejorar la sensibilización, tanto entre el personal de salud como en el conjunto del sector de DSDR, de que el uso de métodos anticonceptivos puede favorecer una vida sexual satisfactoria y segura.

- Integrar de forma sistemática la información disponible sobre la aceptabilidad sexual de los métodos en la consejería en planificación familiar (por ejemplo, orientando a las personas sobre los posibles impactos en distintos aspectos de su vida sexual) para garantizar una toma de decisiones informada.
- Brindar apoyo y soluciones para abordar los desafíos conocidos en materia de aceptabilidad sexual (por ejemplo, ofrecer lubricantes en los métodos que pueden causar sequedad vaginal).
- Garantizar la formación del personal de salud para normalizar la conversación sobre la vida sexual durante la consejería anticonceptiva y empoderar a las personas para que expresen sus preguntas o preocupaciones.

En cuanto a las implicaciones programáticas, recomendamos fortalecer la consejería anticonceptiva para que incorpore la dimensión de la aceptabilidad sexual de los diferentes métodos anticonceptivos. Reconocemos que algunos y algunas profesionales de la salud ya abordan temas vinculados con la vida sexual, como los efectos sobre la libido (por ejemplo, la edición 2022 del manual *Family Planning: A Global Handbook for Providers (Planificación Familiar: Manual General para Proveedores)*[65] ofrece cierta orientación sobre cómo algunos métodos pueden o no afectar el deseo sexual) o la posibilidad de que la pareja perciba el anillo. Sin embargo, según la experiencia programática y nuestros resultados, este tipo de consejería no se aplica de manera sistemática ni proactiva, y aún puede mejorar. La capacitación del personal y las herramientas de apoyo deberían normalizar la conversación sobre las relaciones sexuales en el contexto de la anticoncepción, creando espacios seguros en los entornos clínicos y comunitarios donde las personas se sientan con confianza para preguntar sobre todo el rango de efectos secundarios y elegir el método que mejor se ajuste a sus preferencias. Promover estas conversaciones desde el inicio, con sensibilidad hacia las circunstancias personales y contextuales de cada persona, es esencial para integrar la sexualidad como un componente central de la promoción de la salud y los derechos sexuales. Esto cobra especial importancia si se considera que una revisión previa encontró que las mujeres que recibieron información sobre los posibles efectos secundarios tenían menos probabilidades de abandonar el método por esa causa.[66] Asimismo, es fundamental ofrecer apoyo adicional para manejar cualquier dificultad o efecto adverso. Por ejemplo, se ha demostrado que el uso de antiinflamatorios no esteroides (AINE) a las personas usuarias del DIU puede ayudar a reducir el sangrado y el dolor.[67] En este mismo sentido, podrían ofrecerse lubricantes cuando se sepa que un método puede provocar sequedad o malestar vaginal.

Conclusión

Existe una amplia literatura que analiza las razones para la discontinuidad del uso de métodos anticonceptivos; sin embargo, a pesar de su importancia, los impactos sobre la vida sexual han recibido muy poca atención. Hasta donde sabemos, esta es la primera revisión sistemática que examina la prevalencia de los motivos relacionados con la vida sexual como causas de discontinuidad o cambio de método anticonceptivo. Nuestros hallazgos muestran que estos factores son comunes, con una prevalencia global del 5%, comparable con las tasa de discontinuidad atribuida a otros motivos ampliamente estudiados, como el

costo, la falta de acceso o la incomodidad en el uso. A partir de estos resultados, proponemos que se realicen inversiones más sustanciales para comprender, medir y respaldar mejor aquello que las personas consideran importante al elegir y utilizar un método anticonceptivo, incluidas sus experiencias y preocupaciones relacionadas con la vida sexual. Esto requiere continuar alejándose de aquellos enfoques históricos centrados en el control de la fertilidad, para avanzar hacia modelos basados en los derechos, que coloquen en el centro las motivaciones, autonomía y necesidades de las personas. En particular, para comprender mejor las preocupaciones vinculadas a la vida sexual, proponemos trabajar hacia una medición más clara y focalizada de las razones de discontinuidad, de la mano de capacitaciones más sólidas para el personal de salud y un mayor apoyo a las personas .

Agradecimientos

Las autoras desean agradecer al Dr. James Kiarie y al Dr. Moazzam Ali por sus aportes técnicos y comentarios en distintas etapas de la revisión. También extendemos nuestro agradecimiento a Giselle Balaciano por sus comentarios durante el desarrollo de la estrategia de búsqueda.

Declaración de conflictos de interés

Las autoras declaran que no existe ningún conflicto de interés potencial con respecto a la autoría y/o la publicación de este artículo.

Financiación

La financiación para MZ, como autora principal y consultora/investigadora independiente, destinada a realizar una revisión con alto rigor metodológico, provino de Vitol Foundation y The Case for Her, canalizada a través de *The Pleasure Project*. Los fondos para incorporar a dos coautoras, CM y RM, provinieron de la Oficina de Población y Salud Reproductiva de la Agencia de los Estados Unidos para el Desarrollo Internacional (USAID), a través de la Red IBP. Este trabajo también recibió financiación del Programa Especial de Investigación, Desarrollo y Capacitación en Reproducción Humana (HRP) del PNUD/UNFPA/UNICEF/OMS/Banco Mundial, un programa conjunto ejecutado por la Organización Mundial de la Salud (OMS).

Declaración de disponibilidad de datos

Nuestros análisis se basan en datos ya existentes provenientes de otros estudios publicados, a los que se puede acceder directamente. Las Tablas 1 y 2 de este manuscrito presentan resúmenes de las principales variables extraídas de los estudios. Para facilitar su consulta, los datos clave extraídos también están disponibles en un archivo CSV, junto con nuestro código, en OSF: https://osf.io/ku48p/

Material complementario

Los datos complementarios de este artículo pueden consultarse en línea en https://doi.org/10.1080/26410397.2025.2552589.

Descargo de responsabilidad

Las personas autoras nombradas son las únicas responsables de los puntos de vista expresados en esta publicación, los cuales no representan necesariamente las decisiones ni las políticas del Programa Especial de Investigación, Desarrollo y Capacitación en Reproducción Humana (HRP) del PNUD/UNFPA/UNICEF/OMS/Banco Mundial ni de la Organización Mundial de la Salud (OMS).

Procedencia

Este artículo no fue encargado y se sometió a revisión externa por pares.

Referencias

[1] United Nations Population Fund. International conference on population and development. About the ICPD. [Internet]; 1994. Disponible en: https://www.unfpa.org/icpd.

[2] World Health Organization. Health topics. Contraception. [Internet]. Disponible en: https://www.who.int/health-topics/contraception#tab=tab_1.

[3] Starbird E, Norton M, Marcus R. Investing in family planning: key to achieving the sustainable development goals. *Glob Health Sci Pract*. 2016;4(2):191–210.

[4] Kavanaugh ML, Anderson RM. Contraception and beyond: the health benefits of services provided at family planning centers. N Y Guttmacher Inst; 2013.

[5] Burkman R, Schlesselman JJ, Zieman M. Safety concerns and health benefits associated with oral contraception. *Am J Obstet Gynecol*. 2004;190(4):S5–22.

[6] Askew I, Raney L, Kerrigan M, et al. Family planning saves maternal and newborn lives: Why universal access to contraception must be prioritized in national maternal and newborn health policies, financing, and programs. *Int J Gynaecol Obstet Off Organ Int Fed Gynaecol Obstet*. 2024;164(2):536–540.

[7] Sully EA, Biddlecom A, Darroch JE, et al. Adding it up: investing in sexual and reproductive health 2019; 2020.

[8] IPPF Europe Network. Family planning saves lives, Factsheet 2012. Countdown to 2015; 2012.

[9] FIGO. Contraception and its benefits [Internet]. Disponible en: https://www.figo.org/contraception-and-its-benefits.

[10] FP2030. Contraceptive discontinuation: reasons, challenges, and solutions [Internet]. Disponible en: https://www.fp2030.org/resources/resources-contraceptive-discontinuation-reasons-challenges-and-solutions/.

[11] Jain AK, Obare F, RamaRao S, et al. Reducing unmet need by supporting women with met need. *Int Perspect Sex Reprod Health*. 2013: 133–141.

[12] Castle S, Askew I. Contraceptive discontinuation: reasons, challenges, and solutions. New York (NY); 2015.

[13] Hardee K, Eggleston E, Wong EL, et al. Unintended pregnancy and women's psychological well-being in Indonesia. *J Biosoc Sci*. 2004;36(5):617–626.

[14] Santelli J, Rochat R, Hatfield-Timajchy K, et al. The measurement and meaning of unintended pregnancy. *Perspect Sex Reprod Health*. 2003: 94–101.

[15] Ali MM, Cleland JG, Shah IH. World Health Organization. Causes and consequences of contraceptive discontinuation: evidence from 60 demographic and health surveys; 2012.

[16] Colli E, Tong D, Penhallegon R, et al. Reasons for contraceptive discontinuation in women 20–39 years old in New Zealand. *Contraception*. 1999;59(4):227–231.

[17] Huber LRB, Hogue CJ, Stein AD, et al. Contraceptive use and discontinuation: findings from the contraceptive history, initiation, and choice study. *Am J Obstet Gynecol*. 2006;194(5):1290–1295.

[18] Stuart JE, Secura GM, Zhao Q, et al. Factors associated with 12-month discontinuation among contraceptive pill, patch, and ring users. *Obstet Gynecol*. 2013;121(2 PART 1):330–336.

[19] Bellizzi S, Mannava P, Nagai M, et al. Reasons for discontinuation of contraception among women with a current unintended pregnancy in 36 low and middle-income countries. *Contraception*. 2020;101(1):26–33.

[20] Higgins JA, Smith NK. The sexual acceptability of contraception: reviewing the literature and building a new concept. *J Sex Res*. 2016;53(4–5):417–456.

[21] Inoue K, Barratt A, Richters J. Does research into contraceptive method discontinuation address women's own reasons? A critical review. *J Fam Plann Reprod Health Care*. 2015;41(4):292–299.

[22] Starrs AM, Ezeh AC, Barker G, et al. Accelerate progress—sexual and reproductive health and rights for all: report of the Guttmacher–Lancet Commission. *Lancet*. 2018;391(10140):2642–2692.

[23] Zaneva M, Guzman-Holst C, Reeves A, et al. The impact of monetary poverty alleviation programs on children's and adolescents' mental health: a systematic review and meta-analysis across low-, middle-, and high-income countries. *J Adolesc Health*. 2022.

[24] Shamseer L, Moher D, Clarke M, et al. Preferred reporting items for systematic review and meta-analysis protocols (PRISMA-P) 2015: elaboración y explicación. *BMJ*. 2015;349.

[25] Staaks J. Systematic review research support [Internet]. OSF; 2020. Disponible en: https://osf.io/tw8cb.

[26] Zaneva M, Philpott A, Singh A, et al. What is the added value of incorporating pleasure in sexual health interventions? A systematic review and meta-analysis. *PLoS One*. 2022;17(2):e0261034.

[27] Lockwood C, Munn Z, Porritt K. Qualitative research synthesis: methodological guidance for systematic reviewers utilizing meta-aggregation. *JBI Evid Implement*. 2015;13(3):179–187.

[28] Balduzzi S, Rücker G, Schwarzer G. How to perform a meta-analysis with R: a practical tutorial. *BMJ Ment Health*. 2019;22(4):153–160.

[29] Borenstein M, Hedges LV, Higgins JP, et al. *Introduction to meta-analysis*. John Wiley & Sons; 2021.

[30] Khader YS, El-Qaderi S, Khader AM. Intrauterine contraceptive device discontinuation among Jordanian women: rate, causes and determinants. *BMJ Sex Reprod Health*. 2006;32(3):161–164.

[31] Nega G, Abera M, Tadele A. Discontinuation rate and associated factors among contraceptive implant users in Kersa district, southwestern Ethiopia. *Arch Public Health*. 2021;79(1):75.

[32] Alvergne A, Stevens R, Gurmu E. Side effects and the need for secrecy: characterising discontinuation of modern contraception and its causes in Ethiopia using mixed methods. *Contracept Reprod Med*. 2017;2:1–16.

[33] Bryant AG, Gottert A, Stuart GS, et al. Reasons for intrauterine device use, discontinuation and non-use in Malawi: a qualitative study of women and their partners. *Afr J Reprod Health*. 2015;19(4):50–57.

[34] Ontiri S, Mutea L, Naanyu V, et al. A qualitative exploration of contraceptive use and discontinuation among women with an unmet need for modern contraception in Kenya. *Reprod Health*. 2021;18:1–10.

[35] Bameka A, Kakaire O, Kaye DK, et al. Early discontinuation of long-acting reversible contraceptives and associated factors among women discontinuing long-acting reversible contraceptives at national referral hospital, Kampala-Uganda; a cross-sectional study. *Contracept Reprod Med*. 2023;8(1):27.

[36] Hofmeyr GJ, Singata-Madliki M, Lawrie TA, et al. Effects of the copper intrauterine device versus injectable progestin contraception on pregnancy rates and method discontinuation among women attending termination of pregnancy services in South Africa: a pragmatic randomized controlled trial. *Reprod Health*. 2016;13:1–8.

[37] Sabatini R, Cagiano R. Comparison profiles of cycle control, side effects and sexual satisfaction of three hormonal contraceptives. *Contraception*. 2006;74(3):220–223.

[38] Landolt NK, Phanuphak N, Teeratakulpisarn N, et al. Uptake and continuous use of copper intrauterine device in a cohort of HIV-positive women. *AIDS Care*. 2013;25(6):710–714.

[39] Armitage CM, Mitchell C, Wigan C, et al. Uptake and continuation rates of the intrauterine system in a university student general practice population in the UK. *J Fam Plann Reprod Health Care*. 2013;39(3):186–189.

[40] Brockmeyer A, Kishen M, Webb A. Experience of IUD/IUS insertions and clinical performance in nulliparous women–a pilot study. *Eur J Contracept Reprod Health Care*. 2008;13(3):248–254.

[41] Chaovisitsaree S, Piyamongkol W, Pongsatha S, et al. One year study of Implanon on the adverse events and discontinuation. *J Med Assoc Thai*. 2005;88(3):314–317.

[42] Wigginton B, Harris ML, Loxton D, et al. The feminisation of contraceptive use: Australian women's accounts of accessing contraception. *Fem Psychol*. 2015;25(2):178–198.

[43] Undie CC, RamaRao S, Mbow FB. Choosing and using the progesterone vaginal ring: women's lived experiences in three African cities. *Patient Prefer Adherence*. 2020: 1761–1770.

[44] Al-Jefout M, Nawaiseh N, Tashman S, et al. Jordanian women's experience with etonogestrel subdermal contraceptive implant in two family planning clinics. *Jordan Med J*. 2015;49(1):27–35.

[45] Bruni V, Pontello V, Luisi S, et al. An open-label, multicentre trial to evaluate the vaginal bleeding pattern of the combined contraceptive vaginal ring NuvaRing®. *Eur J Obstet Gynecol Reprod Biol*. 2008;139(1):65–71.

[46] Regidor PA, Colli E, Palacios S. Overall and bleeding-related discontinuation rates of a new oral contraceptive containing 4 mg drospirenone only in a 24/4 regimen and comparison to 0.075 mg desogestrel. *Gynecol Endocrinol*. 2021;37(12):1121–1127.

[47] Gubrium A. "I've lost My mojo, baby" a narrative perspective on the effect of Depo-Provera on Libido. *Sex Res Soc Policy*. 2011;8:321–334.

[48] Lete I, Pérez-Campos E, Correa M, et al. Continuation rate of combined hormonal contraception: a prospective multicenter study. *J Womens Health*. 2012;21(5):490–495.

[49] Berry DC, Raynor D, Knapp P, et al. Patients' understanding of risk associated with medication use: impact of European Commission guidelines and other risk scales. *Drug Saf*. 2003;26:1–11.

[50] NHS. Education for Scotland. Adverse drug reactions. Module 1 basic principles of adverse drug reactions.

[51] Bajracharya A, Veasnakiry L, Rathavy T, et al. Increasing uptake of long-acting reversible contraceptives in Cambodia through a voucher program: evidence from a difference-in-differences analysis. *Glob Health Sci Pract*. 2016;4(Suplemento 2):S109–S121.

[52] Ali M, Azmat SK, Hamza HB, et al. Are family planning vouchers effective in increasing use, improving equity and reaching the underserved? An evaluation of a voucher program in Pakistan. *BMC Health Serv Res*. 2019;19:1–12.

[53] Ngo TD, Nuccio O, Pereira SK, et al. Evaluating a LARC expansion program in 14 sub-Saharan African countries: a service delivery model for meeting FP2020 goals. *Matern Child Health J*. 2017;21:1734–1743.

[54] Coeytaux F, Donaldson D, Aloui T, et al. An evaluation of the cost-effectiveness of mobile family planning services in Tunisia. *Stud Fam Plann*. 1989;20(3):158–169.

[55] Charyeva Z, Oguntunde O, Orobaton N, et al. Task shifting provision of contraceptive implants to community health extension workers: results of operations research in northern Nigeria. *Glob Health Sci Pract*. 2015;3(3):382–394.

[56] Ouedraogo L, Habonimana D, Nkurunziza T, et al. Towards achieving the family planning targets in the African region: a rapid review of task sharing policies. *Reprod Health*. 2021;18:1–12.

[57] Engelbert Bain L, Amu H, Enowbeyang Tarkang E. Barriers and motivators of contraceptive use among young people in Sub-Saharan Africa: A systematic review of qualitative studies. *PLoS One*. 2021;16(6):e0252745.

[58] Harvey C, Seib C, Lucke J. Continuation rates and reasons for removal among implanon® users accessing two family planning clinics in Queensland, Australia. *Contraception*. 2009;80(6):527–532.

[59] Speizer IS, Bremner J, Farid S. Language and measurement of contraceptive need and making these indicators more meaningful for measuring fertility intentions of women and girls. *Glob Health Sci Pract*. 2022;10(1).

[60] Fabic MS. What do we demand? Responding to the call for precision and definitional agreement in family planning's "demand" and "need" jargon. *Glob Health Sci Pract*. 2022;10(1).

[61] Peyman N, Oakley D. Effective contraceptive use: an exploration of theory-based influences. *Health Educ Res*. 2009;24(4):575–585.

[62] Dimitrov R, Jelen A, L'Etang J. *Taboos in health communication: stigma, silence and voice. Vol. 11, Public Relations Inquiry*. Londres, Reino Unido: SAGE Publications Sage UK; 2022.

[63] Hoggart L, Newton VL. Young women's experiences of side-effects from contraceptive implants: a challenge to bodily control. *Reprod Health Matters*. 2013;21(41):196–204.

[64] Olaifa BT, Okonta HI, Mpinda JB, et al. Reasons given by women for discontinuing the use of progestogen implants at Koster Hospital, North West province. *South Afr Fam Pract*. 2022;64(4).

[65] Family Planning: A Global Handbook for Providers. Editors World Health Organization and Johns Hopkins Bloomberg School of Public Health/Center for Communication Programs; 2022.

[66] Danna K, Angel A, Kuznicki J, et al. Leveraging the client-provider interaction to address contraceptive discontinuation: a scoping review of the evidence that links them. *Glob Health Sci Pract*. 2021;9(4):948–963.

[67] Grimes DA, Lopez LM, Manion C, et al. Cochrane systematic reviews of IUD trials: lessons learned. *Contraception*. 2007;75(6):S55–S59.
