# Supplementary material for: The sex effect: the prevalence of sex life reasons for contraceptive discontinuation. A systematic review and meta-analysis
Source: Sex Reprod Health Matters. 2025 Sep 25;33(1):2552589. doi: 10.1080/26410397.2025.2552589 (PMC12557827; doi:10.1080/26410397.2025.2552589)
Supplement: Spanish translation of Supplemental Material [file ZRHM_A_2552589_SM0092.pdf]

Contenido del material suplementario

1. Estrategia de búsqueda

2. Resultados de las meta-regresiones

3. Resumen de la evaluación del riesgo de sesgo

3.1 Estudios cuantitativos

3.2 Estudios cualitativos

4. Figuras y tablas suplementarias

5. Referencias completas de los estudios incluidos

1. Estrategia de búsqueda

En términos generales, nuestra estrategia de búsqueda combina términos MeSH y palabras clave con la siguiente lógica: (Contraception) AND ((Discontinuation) OR (Switching)).

Para especificar aún más la búsqueda, seleccionamos manualmente filtros que nos permitieran capturar el periodo temporal de interés, incluir únicamente estudios realizados en humanos (es decir, excluir estudios en animales) y en el rango etario apropiado (por ejemplo, excluir bebés, infantes y niños pequeños), así como conservar los tipos de artículos relevantes (por ejemplo, excluir preprints).

Sintaxis completa de búsqueda en PubMed:
Search: (Contraception[Mesh] OR "Contraception, Postcoital"[Mesh] OR "Contraception, Barrier"[Mesh] OR "Contraception, Immunologic"[Mesh] OR "Contraception Behavior"[Mesh] OR "Hormonal Contraception"[Mesh] OR "Long-Acting Reversible Contraception"[Mesh] OR "Contraceptive Effectiveness"[Mesh] OR "Reproductive Control Agents"[Mesh] OR "Medroxyprogesterone Acetate"[Mesh] OR Desogestrel[Mesh] OR "Contraceptives, Oral"[Mesh] OR "cervical cap"[Title/Abstract] OR "cervical caps"[Title/Abstract] OR "coiled spring*"[Title/Abstract] OR "vaginal ring*"[Title/Abstract] OR "vaginal diaphragm*"[Title/Abstract] OR "vaginal shield*"[Title/Abstract] OR "contraceptive device*"[Title/Abstract] OR "Intrauterine Device*"[Title/Abstract] OR IUD[Title/Abstract] OR IUS[Title/Abstract] OR “family plan*”[Title/Abstract] OR "reproductive control agent*"[Title/Abstract] OR abortifacient*[Title/Abstract] OR "Depo-Provera"[Title/Abstract] OR DMPA[Title/Abstract] OR "Oral Contraceptives Low-Dose"[Title/Abstract] OR "Oral contraceptive hormonal"[Title/Abstract] OR "Postcoital Contraceptive*"[Title/Abstract] OR "Emergency Contraceptive*"[Title/Abstract] OR "Emergency Contraceptive*"[Title/Abstract] OR "Morning After Pill"[Title/Abstract] OR "Morning-After Pill"[Title/Abstract] OR "Voluntary Female Sterilization*"[Title/Abstract] OR "Female Sterilization*"[Title/Abstract] OR "Male Sterilization*"[Title/Abstract] OR vasectomy[Title/Abstract]) AND (continu* OR discontinu* OR "discontinuation rate*" OR switch* OR reverse)
Filtros: Artículo clásico, Estudio clínico, Ensayo clínico, Ensayo clínico controlado, Artículo corregido y republicado, Metaanálisis, Estudio observacional, Ensayo controlado aleatorizado, Revisión, Revisión sistemática, Humanos, Inglés, Adolescente: 13–18 años, Adulto: 19+ años,

Adulto joven: 19–24 años, Adulto: 19–44 años, Mediana edad: 45–64 años, Exclusión de preprints, de 2004 a 2023.

Seleccionamos manualmente los términos apropiados y ajustamos la sintaxis para cada base de datos. Luego utilizamos la herramienta Polyglot de https://www.sr-accelerator.com/#/polyglot para verificar nuestra búsqueda y asegurar que no hubiera errores de sintaxis entre las distintas bases de datos.

Clasificación de la information: General

2. Resultados de las meta-regresiones

Modelo		Estimación (IC)	EE	-95% IC	+95% IC	Valor z
1. Meta-regresión univariada para el contenido hormonal del método examinado, Nivel de referencia = Métodos no hormonales						
	Intercepto	-3.37***	0.75	-4.84	-1.91	-4.52
	Ambos	-0.39	1.21	-2.75	1.98	-0.32
	Hormonales	-0.59	0.80	-0.98	2.17	0.74
	No claro	-0.83	1.37	-3.53	1.86	-0.60
2. Meta-regresión univariada para el método anticonceptivo específico, Nivel de referencia = DIU de cobre						
	Intercepto	-3.34***	0.88	-5.05	-1.62	-3.81
	Implante	0.12	1.11	-2.06	2.30	0.11
	Inyección	0.61	1.44	-2.21	3.43	0.42
	LNG-SIU	0.96	1.24	-1.47	3.40	0.78
	Píldora	-0.33	1.37	-3.01	2.35	-0.24
	No claro	-0.87	1.70	-4.19	2.46	-0.51
	Anillo vaginal	1.63	1.47	-1.25	4.52	1.11
	Varios	0.38	1.06	-1.69	2.46	0.36
3. Meta-regresión univariada por tipo de motivo según el marco de Higgins y Smith Nivel de referencia = Funcionamiento sexual, especialmente libido						
	Intercepto	-3.16***	0.44	-4.02	-2.30	-7.18
	Preocupación por el placer o funcionamiento de la pareja	0.51	0.78	-1.02	2.04	0.66
	Placer y búsqueda de placer	-2.59	2.05	-6.61	1.44	-1.26
	Preferencias/Estética sexual	0.41	0.10	-1.55	2.37	0.41
	No claro	0.29	0.95	-1.56	2.15	0.31
4. Meta-regresión univariada por nivel del motivo según el marco de Higgins y Smith Nivel de referencia = Individual						
	Intercepto	-3.17***	0.38	-3.92	-2.43	-8.32
	Relación	0.52	0.74	-0.93	1.98	0.71
	No claro	0.31	0.91	-1.47	2.09	0.34
5. Meta-regresión univariada por riesgo de sesgo / evaluación de calidad Nivel de referencia = Puntuación global deficiente						
	Intercepto	-2.90***	0.57	-5.04	-4.03	-1.78
	Aceptable	0.04	0.74	0.05	-1.41	1.49
	Buena	-0.30	0.73	0.73	-1.73	1.14
6. Meta-regresión multivariada considerando el contenido hormonal del método examinado y el método anticonceptivo específico Nivel de referencia = No hormonal						
	Intercepto	-3.34***	0.86	-5.03	1.65	-3.88

	Ambos	-0.37	1.41	-3.13	2.39	-0.26
	Hormonales	0.60	1.05	-1.46	2.66	0.57
	No claro (hormonal)	-0.86	1.66	-4.11	2.38	-0.52
	Implante	-0.39	0.97	-2.29	1.50	-0.41
	Inyección	0.04	1.27	-2.45	2.53	0.03
	LNG-SIU	0.62	1.25	-1.84	3.07	0.49
	Píldora	-0.92	1.19	-3.25	1.41	-0.77
	Anillo vaginal	1.35	1.68	-1.94	4.65	0.81

Para los valores p: *** indica $p < .0001$. Todos los demás valores p son superiores a .05.

Aunque todos los modelos de meta-regresión son estimables, advertimos que en algunos casos se basan en muestras muy pequeñas y pocos datos (estudios), por lo que deben interpretarse con mucha precaución.

En el Modelo 1, 'Ambos' se refiere a estudios que examinan tanto métodos hormonales como no hormonales. 'No claro' indica estudios en los que no está claro si el método anticonceptivo es hormonal o no hormonal.

En el Modelo 2, 'No claro' indica estudios en los que no se especifica qué métodos específicos se usaron; 'Varios' se refiere a múltiples métodos examinados en un mismo estudio, donde no fue posible desagregar por método específico.

En el Modelo 3, 'No claro' se refiere a estudios donde no se especifica qué categoría de motivo relevante corresponde según el marco de Higgins y Smith (2016). De manera análoga, en el Modelo 4 'No claro' se refiere a estudios donde no se especifica qué nivel de motivo relevante corresponde según el mismo marco.

3. Resumen de la evaluación del riesgo de sesgo

3.1 Estudios cuantitativos

Al evaluar los estudios de cohorte, utilizamos la Herramienta de Evaluación de Calidad del Instituto Nacional del Corazón, Pulmones y Sangre (NHLBI, por sus siglas en inglés) para estudios observacionales de cohorte y transversales. En el caso de los estudios de intervención no aleatorizados, recurrimos a la Herramienta de Evaluación de Calidad del NHLBI para estudios de intervención controlados. Las herramientas del NHLBI producen una calificación global de calidad: Buena – Aceptable – Deficiente.

Para los ensayos controlados aleatorizados, utilizamos la herramienta de riesgo de sesgo de Cochrane para ensayos aleatorizados (RoB 2). La puntuación general de esta herramienta varía desde 'Bajo riesgo de sesgo', 'Algunas preocupaciones', hasta 'Alto riesgo de sesgo'. Para mantener la uniformidad, renombramos estas puntuaciones como Deficiente – Aceptable – Buena, respectivamente (por ejemplo, 'Alto riesgo de sesgo' se designa como 'Deficiente' a continuación).

El número que sigue a cada estudio (por ejemplo, (1), (2), etc.) permite confirmar la referencia completa del estudio en la sección 5 de este material suplementario.

Estudio	Herramienta de cribado	Evaluación global
Al-Jefout et al. (2015) (1)	NHLBI	Deficiente
Armitage, Mitchell, Wigan, & Smith (2012) (2)	NHLBI	Deficiente
Awoyesuku, Altraide, & Amadi, (2021) (3)	NHLBI	Aceptable
Bachofner et al. (2018) (4)	NHLBI	Aceptable
Bameka, Kakaire, Kaye, & Namusoke (2023) (5)	NHLBI	Buena
Barreiros et al. (2007) (6)	NHLBI	Buena
Beyene et al. (2022) (7)	NHLBI	Buena
Blumenthal, Gemzell-Danielsson, & Marintcheva-Petrova (2008) (8)	NHLBI	Deficiente
Brockmeyer et al. (2008) (9)	NHLBI	Buena
Bruni et al. (2008) (10)	NHLBI	Aceptable
Chaovisitsaree et al. (2005) (11)	NHLBI	Deficiente
Daud & Ewies (2008) (12)	NHLBI	Aceptable
Ekabua & Itam (2007) (13)	NHLBI	Aceptable
Frederico, Silva dos Santos, Ferreira, Bahamondes, & Fernandes (2022) (14)	NHLBI	Buena
Friedman (2015) (15)	NHLBI	Aceptable
Fruzzetti et al. (2016) (16)	NHLBI	Aceptable
Hajikazemi, Nikpour, Haghani (2004) (17)	NHLBI	Deficiente
Hines et al. (2022) (18)	NHLBI	Buena
Hofmeyr et al. (2016) (19)	RoB - 2	Deficiente

Iftikhar, Shaheen, Arora (2019) (20)	NHLBI	Deficiente
Keogh et al. (2021) (21)	NHLBI	Buena
Khader, El-Qaderi, Khader (2006) (22)	NHLBI	Aceptable
Kriplani et al. (2019) (23)	RoB - 2	Aceptable
Landolt et al. (2013) (24)	NHLBI	Buena
Lathrop et al. (2020) (25)	NHLBI	Buena
Lete et al. (2012) (26)	NHLBI	Aceptable
Littlejohn (2012) (27)	NHLBI	Buena
Madden et al. (2012) (28)	NHLBI	Buena
Merki-Feld & Hund (2010) (29)	NHLBI	Deficiente
Mrwebi et al. (2018) (30)	NHLBI	Buena
Park, Nguyen & Ngo (2011) (31)	NHLBI	Buena
Parkpinyo, Panichyawat, Sirimai (2021) (32)	NHLBI	Buena
Regidor, Colli, Palacios (2021) (33)	RoB – 2	Deficiente
Rothschild et al. (2022) (34)	NHLBI	Aceptable
Sabatini and Cagiano (2006) (35)	RoB - 2	Aceptable
Saloranta et al. (2020) (36)	NHLBI	Buena
Sarnak et al. (2023) (37)	NHLBI	Buena
Schafer, Osborne, Davis & Westhoff, (2006) (38)	RoB - 2	Deficiente
Simmons et al. (2019) (39)	NHLBI	Buena
Ssebatta, Kaye, Mbalinda (2021) (40)	NHLBI	Aceptable
Warner et al. (2010) (41)	RoB - 2	Buena
Wojcik et al. (2022) (42)	NHLBI	Buena

Clasificación de la information: General

3.2 Estudios cualitativos

Algunos de los estudios siguientes son de métodos mixtos e incluyen componentes cuantitativos y cualitativos. Estos estudios se evaluaron con la Herramienta de Evaluación Crítica del Instituto Joanna Briggs para estudios cualitativos, ya que en todos ellos las motivaciones sexuales para la discontinuidad se reportan en la metodología cualitativa. Esta herramienta produce una evaluación global con tres posibles resultados: 'Incluir', 'Excluir' o 'Solicitar más información'. Para mantener la uniformidad con las otras herramientas, renombramos estas categorías como 'Buena', 'Deficiente' y 'Aceptable', respectivamente.

El número que sigue a cada estudio (por ejemplo, (43), (44), etc.) permite confirmar la referencia completa del estudio en la sección 5 de este material suplementario.

**Estudio**	**Evaluación global**
Alvergne, Stevens & Gurmu (2017) (43)	Aceptable
Berglas et al. (2021) (44)	Buena
Brunie et al. (2022) (45)	Buena
Bryant et al. (2015) (46)	Buena
Chin-Quee et al. (2022) (47)	Buena
Coombe, Harris, Loxton (2019) (48)	Buena
Dalessandro, Thorpe, Sanders (2022) (49)	Buena
Epstein (2008) (50)	Buena
Gubrium (2011) (51)	Buena
Hoggart & Newton (2013) (52)	Buena
Imbuki et al. (2010) (53)	Aceptable
Khalaf (2004) (54)	Buena
Kibira, Muhumuza, Bukenya, Atuyambe (2015) (55)	Buena
Mills & Barclay (2006) (56)	Aceptable
Mihretie et al. (2023) (57)	Buena
Mwizerwa & Rozzano (2011) (58)	Deficiente
Nega, Abera, Tadele (2021) (59)	Deficiente
Obare, Odwe, & Cleland (2021) (60)	Aceptable
Olaifa et al. (2022) (61)	Buena
Ontiri et al. (2021) (62)	Buena
Undie, RamaRao, Mbow (2020) (63)	Buena
Wigginton et al. (2015) (64)	Buena

4. Tablas y figuras suplementarias

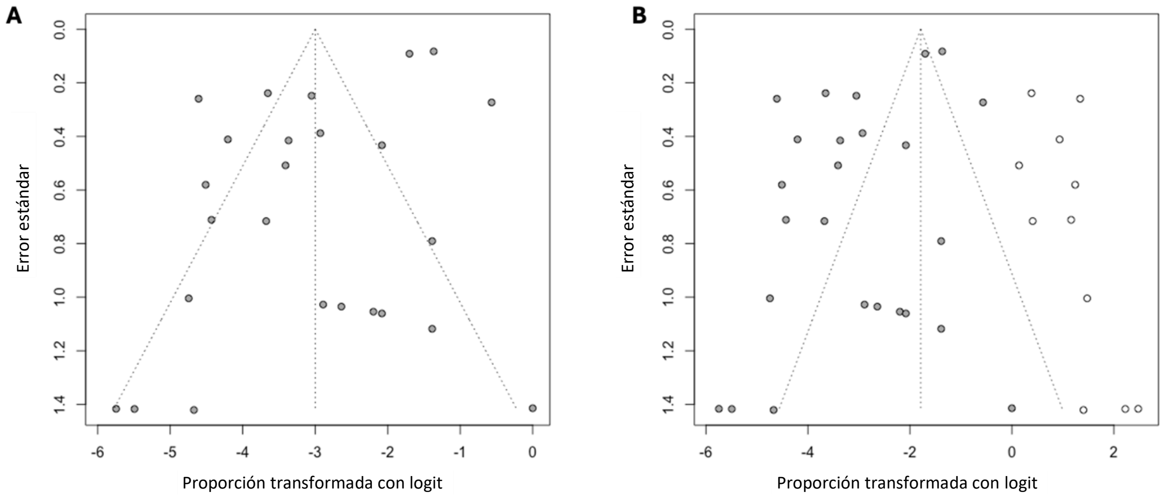

Figura 1. Panel A. Gráfico en embudo (funnel plot). Panel B. Gráfico en embudo posterior al procedimiento de recorte y relleno (trim and fill).

Estudio	PLOGIT	EE(PLOGIT)	Proporción	IC 95%	Peso
Armitage, Mitchell, Wigan, & Smith (2012)	-2.1972	1.0541	0.10	[0.01; 0.47]	2.3%
Awoyesuku, Altraide, & Amadi, (2021)	-4.5109	0.5805	0.01	[0.00; 0.03]	3.1%
Barreiros et al. (2006)	-1.3863	0.7906	0.20	[0.05; 0.54]	2.7%
Brockmeyer et al. (2008)	-2.6391	1.0351	0.07	[0.01; 0.35]	2.4%
Chaovisitsaree et al. (2005)	-1.3863	1.1180	0.20	[0.03; 0.69]	2.2%
Friedman (2015)	-2.0794	1.0607	0.11	[0.02; 0.50]	2.3%
Fruzzetti et al. (2016)	-3.6535	0.2387	0.03	[0.02; 0.04]	3.4%
Hajikazemi, Nikpour, Haghani (2004)	-1.3656	0.0828	0.20	[0.18; 0.23]	3.5%
Hofmeyr et al. (2019)	-4.7449	1.0043	0.01	[0.00; 0.06]	2.4%
Hofmeyr et al. (2019)	-5.4931	1.4171	0.00	[0.00; 0.06]	1.8%
Keogh et al. (2021)	-5.7462	1.4165	0.00	[0.00; 0.05]	1.8%
Kriplani et al. (2019)	-3.4095	0.5082	0.03	[0.01; 0.08]	3.2%
Landolt et al. (2013)	0.0000	1.4142	0.50	[0.06; 0.94]	1.8%
Lete et al. (2012)	-1.7002	0.0916	0.15	[0.13; 0.18]	3.5%
Littlejohn (2012)	-4.6085	0.2595	0.01	[0.01; 0.02]	3.4%
Madden et al. (2012)	-3.3673	0.4152	0.03	[0.02; 0.07]	3.3%
Mrwebi et al. (2018)	-4.4308	0.7113	0.01	[0.00; 0.05]	2.9%
Park, Nguyen & Ngo (2011)	-4.2047	0.4113	0.01	[0.01; 0.03]	3.3%
Regidor, Colli, Palacios (2021)	-3.6763	0.7160	0.02	[0.01; 0.09]	2.9%
Sabatini and Cagiano (2006)	-0.5664	0.2732	0.36	[0.25; 0.49]	3.4%
Saloranta et al. (2020)	-3.0501	0.2482	0.05	[0.03; 0.07]	3.4%
Saloranta et al. (2020)	-4.6728	1.4208	0.01	[0.00; 0.13]	1.8%
Schafer, Osborne, Davis & Westhoff, (2006)	-2.8904	1.0274	0.05	[0.01; 0.29]	2.4%
Ssebatta, Kaye, Mbalinda (2021)	-2.9293	0.3879	0.05	[0.02; 0.10]	3.3%
Wjocik et al. (2022)	-2.0794	0.4330	0.11	[0.05; 0.23]	3.3%
Imputado: Kriplani et al. (2019)	0.1409	0.5082	0.54	[0.30; 0.76]	3.2%
Imputado: Fruzzetti et al. (2016)	0.3849	0.2387	0.60	[0.48; 0.70]	3.4%
Imputado: Regidor, Colli, Palacios (2021)	0.4077	0.7160	0.60	[0.27; 0.86]	2.9%
Imputado: Park, Nguyen & Ngo (2011)	0.9360	0.4113	0.72	[0.53; 0.85]	3.3%
Imputado: Mrwebi et al. (2018)	1.1622	0.7113	0.76	[0.44; 0.93]	2.9%
Imputado: Awoyesuku, Altraide, & Amadi, (2021)	1.2422	0.5805	0.78	[0.53; 0.92]	3.1%
Imputado: Littlejohn (2012)	1.3399	0.2595	0.79	[0.70; 0.86]	3.4%
Imputado: Saloranta et al. (2020)	1.4042	1.4208	0.80	[0.20; 0.99]	1.8%
Imputado: Hofmeyr et al. (2019)	1.4763	1.0043	0.81	[0.38; 0.97]	2.4%
Imputado: Hofmeyr et al. (2019)	2.2244	1.4171	0.90	[0.37; 0.99]	1.8%
Imputado: Keogh et al. (2021)	2.4776	1.4165	0.92	[0.43; 0.99]	1.8%
**Modelo de efectos aleatorios**			**0.14**	**[0.09; 0.22]**	**100%**

Heterogeneidad: $I^2 = 95\%$, $\tau^2 = 2.1636$, $p < 0.01$

Figura 2. Gráfico de bosque (forest plot) posterior al procedimiento de recorte y relleno.

Estudio	Eventos	Total	Proporción	IC 95%	Peso
**Tipo = Funcionamiento sexual, especialmente libido**					
Chaovisitsaree et al. (2005)	1	5	0.20	[0.01; 0.72]	2.6%
Friedman (2015)	1	9	0.11	[0.00; 0.48]	2.7%
Fruzzetti et al. (2016)	18	713	0.03	[0.02; 0.04]	5.0%
Hajikazemi, Nikpour, Haghani (2004)	183	900	0.20	[0.18; 0.23]	5.2%
Hofmeyr et al. (2019)	1	237	0.00	[0.00; 0.02]	2.9%
Keogh et al. (2021)	0	156	0.00	[0.00; 0.02]	2.0%
Madden et al. (2012)	6	180	0.03	[0.01; 0.07]	4.6%
Mrwebi et al. (2018)	2	170	0.01	[0.00; 0.04]	3.7%
Regidor, Colli, Palacios (2021)	2	81	0.02	[0.00; 0.09]	3.7%
Sabatini y Cagiano (2006)	12	58	0.21	[0.11; 0.33]	4.8%
Saloranta et al. (2020)	17	430	0.04	[0.02; 0.06]	5.0%
Ssebatta, Kaye, Mbalinda (2021)	7	138	0.05	[0.02; 0.10]	4.7%
Wjocik et al. (2022)	1	54	0.02	[0.00; 0.10]	2.9%
**Modelo de efectos aleatorios**		3131	0.04	[0.02; 0.09]	49.8%
Heterogeneidad: $I^2 = 94\%$, $\tau^2 = 1.6848$, $p < 0.01$					
**Tipo = Preocupación por el placer o el funcionamiento de la pareja**					
Armitage, Mitchell, Wigan & Smith (2012)	1	10	0.10	[0.00; 0.45]	2.8%
Awoyesuku, Altraide & Amadi (2021)	3	276	0.01	[0.00; 0.03]	4.1%
Barreiros et al. (2006)	1	10	0.10	[0.00; 0.45]	2.8%
Brockmeyer et al. (2008)	1	15	0.07	[0.00; 0.32]	2.8%
Kriplani et al. (2019)	4	125	0.03	[0.01; 0.08]	4.3%
Landolt et al. (2013)	1	2	0.50	[0.01; 0.99]	2.0%
Schafer, Osborne, Davis & Westhoff (2006)	1	9	0.11	[0.00; 0.48]	2.7%
**Modelo de efectos aleatorios**		447	0.06	[0.02; 0.14]	21.5%
Heterogeneidad: $I^2 = 56\%$, $\tau^2 = 0.8979$, $p = 0.03$					
**Tipo = Placer y búsqueda de placer**					
Keogh et al. (2021)	0	156	0.00	[0.00; 0.02]	2.0%
**Tipo = Preferencias sexuales / Estética sexual**					
Barreiros et al. (2006)	1	10	0.10	[0.00; 0.45]	2.8%
Park, Nguyen & Ngo (2011)	6	408	0.01	[0.01; 0.03]	4.6%
Sabatini y Cagiano (2006)	9	58	0.16	[0.07; 0.27]	4.7%
**Modelo de efectos aleatorios**		**476**	**0.06**	**[0.01; 0.30]**	**12.1%**
Heterogeneidad: $I^2 = 91\%$, $\tau^2 = 2.4288$, $p < 0.01$					
**Tipo = No claro**					
Lete et al. (2012)	141	913	0.15	[0.13; 0.18]	5.2%
Littlejohn (2012)	15	1520	0.01	[0.01; 0.02]	5.0%
Wjocik et al. (2022)	5	54	0.09	[0.03; 0.20]	4.4%
**Modelo de efectos aleatorios**		**2487**	**0.05**	**[0.01; 0.31]**	**14.6%**
Heterogeneidad: $I^2 = 98\%$, $\tau^2 = 3.2046$, $p < 0.01$					
**Modelo de efectos aleatorios**		**6697**	**0.05**	**[0.03; 0.08]**	**100%**
Heterogeneidad: $I^2 = 93\%$, $\tau^2 = 1.2370$, $p < 0.01$					
Prueba de diferencias entre subgrupos: $\chi^2_4 = 4.10$, gl = 4, $p = 0.39$					

Figura 3. Gráfico de bosque que presenta los resultados de los análisis por subgrupos basados en la categoría específica de motivos, de acuerdo con el marco de Higgins y Smith (2016).

Clasificación de la information: General

Estudio	Eventos	Total	Proporción	IC 95%	Peso
**Tipo = Relación**					
Armitage, Mitchell, Wigan & Smith (2012)	1	10	0.10	[0.00; 0.45]	2.8%
Awoyesuku, Altraide & Amadi (2021)	3	276	0.01	[0.00; 0.03]	4.1%
Barreiros et al. (2006)	1	10	0.10	[0.00; 0.45]	2.8%
Brockmeyer et al. (2008)	1	15	0.07	[0.00; 0.32]	2.8%
Kriplani et al. (2019)	4	125	0.03	[0.01; 0.08]	4.3%
Landolt et al. (2013)	1	2	0.50	[0.01; 0.99]	2.0%
Schafer, Osborne, Davis & Westhoff (2006)	1	9	0.11	[0.00; 0.48]	2.7%
**Modelo de efectos aleatorios**		**447**	**0.06**	**[0.02; 0.14]**	**21.5%**
Heterogeneidad: I^2 = 56%, τ^2 = 0.8979, p = 0.03					
**Tipo = Individual**					
Barreiros et al. (2006)	1	10	0.10	[0.00; 0.45]	2.8%
Chaovisitsaree et al. (2005)	1	5	0.20	[0.01; 0.72]	2.6%
Friedman (2015)	1	9	0.11	[0.00; 0.48]	2.7%
Fruzzetti et al. (2016)	18	713	0.03	[0.02; 0.04]	5.0%
Hajikazemi, Nikpour, Haghani (2004)	183	900	0.20	[0.18; 0.23]	5.2%
Hofmeyr et al. (2019)	1	237	0.00	[0.00; 0.02]	2.9%
Keogh et al. (2021)	0	156	0.00	[0.00; 0.02]	2.0%
Keogh et al. (2021)	0	156	0.00	[0.00; 0.02]	2.0%
Madden et al. (2012)	6	180	0.03	[0.01; 0.07]	4.6%
Mrwebi et al. (2018)	2	170	0.01	[0.00; 0.04]	3.7%
Park, Nguyen & Ngo (2011)	6	408	0.01	[0.01; 0.03]	4.6%
Regidor, Colli, Palacios (2021)	2	81	0.02	[0.00; 0.09]	3.7%
Sabatini y Cagiano (2006)	12	58	0.21	[0.11; 0.33]	4.8%
Sabatini y Cagiano (2006)	9	58	0.16	[0.07; 0.27]	4.7%
Saloranta et al. (2020)	17	430	0.04	[0.02; 0.06]	5.0%
Ssebatta, Kaye, Mbalinda (2021)	7	138	0.05	[0.02; 0.10]	4.7%
Wjocik et al. (2022)	1	54	0.02	[0.00; 0.10]	2.9%
**Modelo de efectos aleatorios**		**3763**	**0.04**	**[0.02; 0.08]**	**63.9%**
Heterogeneidad: I^2 = 93%, τ^2 = 1.6701, p < 0.01					
**Tipo = No claro**					
Lete et al. (2012)	141	913	0.15	[0.13; 0.18]	5.2%
Littlejohn (2012)	15	1520	0.01	[0.01; 0.02]	5.0%
Wjocik et al. (2022)	5	54	0.09	[0.03; 0.20]	4.4%
**Modelo de efectos aleatorios**		**2487**	**0.05**	**[0.01; 0.31]**	**14.6%**
Heterogeneidad: I^2 = 98%, τ^2 = 3.2046, p < 0.01					
**Modelo de efectos aleatorios (global)**		**6697**	**0.05**	**[0.03; 0.08]**	**100%**
Heterogeneidad: I^2 = 93%, τ^2 = 1.2370, p < 0.01					
Prueba de diferencias entre subgrupos: χ^2_2 = 0.41, gl = 2, p = 0.81					

Figura 4. Gráfico de bosque que presenta los resultados de los análisis por subgrupos basados en el nivel específico de motivos, de acuerdo con el marco de Higgins y Smith (2016).

Clasificación de la information: General

Estudio	Eventos	Total	Proporción	IC 95%	Peso
**Riesgo de sesgo = Deficiente**					
Armitage, Mitchell, Wigan, & Smith (2012)	1	10	0.10	[0.00; 0.45]	3.0%
Chaovisitsaree et al. (2005)	1	5	0.20	[0.01; 0.72]	2.9%
Hajikazemi, Nikpour, Haghani (2004)	183	900	0.20	[0.18; 0.23]	5.5%
Hofmeyr et al. (2019)	1	116	0.01	[0.00; 0.05]	3.2%
Hofmeyr et al. (2019)	0	121	0.00	[0.00; 0.03]	2.2%
Regidor, Colli, Palacios (2021)	2	81	0.02	[0.00; 0.09]	4.0%
Schafer, Osborne, Davis & Westhoff (2006)	1	19	0.05	[0.00; 0.26]	3.1%
**Modelo de efectos aleatorios**		**1252**	**0.05**	**[0.01; 0.16]**	**23.9%**
Heterogeneidad: $I^2 = 81\%$, $\tau^2 = 2.1947$, $p < 0.01$					
**Riesgo de sesgo = Aceptable**					
Awoyesuku, Altraide & Amadi (2021)	3	276	0.01	[0.00; 0.03]	4.4%
Friedman (2015)	1	9	0.11	[0.00; 0.48]	3.0%
Fruzzetti et al. (2016)	18	713	0.03	[0.02; 0.04]	5.3%
Kriplani et al. (2019)	4	125	0.03	[0.01; 0.08]	4.7%
Lete et al. (2012)	141	913	0.15	[0.13; 0.18]	5.5%
Mrwebi et al. (2018)	2	170	0.01	[0.00; 0.04]	4.0%
Sabatini y Cagiano (2006)	21	58	0.36	[0.24; 0.50]	5.3%
Ssebatta, Kaye, Mbalinda (2021)	7	138	0.05	[0.02; 0.10]	5.0%
**Modelo de efectos aleatorios**		2402	0.05	[0.02; 0.13]	37.3%
Heterogeneidad: $I^2 = 95\%$, $\tau^2 = 1.5814$, $p < 0.01$					
**Riesgo de sesgo = Bueno**					
Barreiros et al. (2006)	2	10	0.20	[0.03; 0.56]	3.8%
Brockmeyer et al. (2008)	1	15	0.07	[0.00; 0.32]	3.1%
Keogh et al. (2021)	0	156	0.00	[0.00; 0.02]	2.2%
Landolt et al. (2013)	1	2	0.50	[0.01; 0.99]	2.2%
Littlejohn (2012)	15	1520	0.01	[0.01; 0.02]	5.3%
Madden et al. (2012)	6	180	0.03	[0.01; 0.07]	4.9%
Park, Nguyen & Ngo (2011)	6	408	0.01	[0.01; 0.03]	4.9%
Saloranta et al. (2020)	17	376	0.05	[0.03; 0.07]	5.3%
Saloranta et al. (2020)	0	53	0.00	[0.00; 0.07]	2.2%
Wjocik et al. (2022)	6	54	0.11	[0.04; 0.23]	4.9%
**Modelo de efectos aleatorios**		2774	0.04	[0.02; 0.08]	38.8%
Heterogeneidad: $I^2 = 83\%$, $\tau^2 = 1.0623$, $p < 0.01$					
**Modelo de efectos aleatorios (global)**		**6428**	**0.05**	**[0.03; 0.08]**	**100.0%**
Heterogeneidad: $I^2 = 94\%$, $\tau^2 = 1.3130$, $p < 0.01$					
Prueba de diferencias entre subgrupos: $\chi^2_2 = 0.33$, df = 2, $p = 0.85$					

Figura 5. Gráfico de bosque que presenta los resultados de los análisis por subgrupos basados en la puntuación global del riesgo de sesgo de los estudios.

5. Referencias completas de los estudios incluidos

1. Al-Jefout M, Nawaiseh N, Tashman S, Ryalat R, Zaitoun S, Al-Alawi L, et al. Jordanian Women's Experience with Etonogestrel Subdermal Contraceptive Implant in Two Family Planning Clinics. Jordan Med J. 2015;49(1):27–35.

2. Armitage CM, Mitchell C, Wigan C, Smith DA. Uptake and continuation rates of the intrauterine system in a university student general practice population in the UK. J Fam Plann Reprod Health Care. 2013;39(3):186–9.

3. Awoyesuku PA, Altraide BO, Amadi SC. Modern contraceptives discontinuation, method switching and associated factors among clients at the family planning clinic of a tertiary hospital in Port-Harcourt, Nigeria. Int J Reprod Contracept Obstet Gynecol. 2021;10(1):6.

4. Bachofner M, Blickenstorfer K, Hutmacher J, Wehrle L, Leeners B, Merki-Feld G. Intrauterine device continuation rates and reasons for discontinuation in a Central European clinic with a high standard of care and ultrasound follow-up: a retrospective cohort study. Eur J Contracept Reprod Health Care. 2018;23(6):407–14.

5. Bameka A, Kakaire O, Kaye DK, Namusoke F. Early discontinuation of long-acting reversible contraceptives and associated factors among women discontinuing long-acting reversible contraceptives at national referral hospital, Kampala-Uganda; a cross-sectional study. Contracept Reprod Med. 2023;8(1):27.

6. Barreiros FA, Guazzelli CAF, de Araújo FF, Barbosa R. Bleeding patterns of women using extended regimens of the contraceptive vaginal ring. Contraception. 2007;75(3):204–8.

7. Beyene GN, Assefa N, Mokonnon TM, Ejigu HB, Yadeta TA. Early Implanon discontinuation and associated factors among Implanon women users visiting public health facilities, in Kembata zone of Southern Ethiopia: An institution based cross-sectional study. Front Glob Womens Health. 2022;3:909411.

8. Blumenthal PD, Gemzell-Danielsson K, Marintcheva-Petrova M. Tolerability and clinical safety of Implanon®. Eur J Contracept Reprod Health Care. 2008;13(sup1):29–36.

9. Brockmeyer A, Kishen M, Webb A. Experience of IUD/IUS insertions and clinical performance in nulliparous women–a pilot study. Eur J Contracept Reprod Health Care. 2008;13(3):248–54.

10. Bruni V, Pontello V, Luisi S, Petraglia F. An open-label, multicentre trial to evaluate the vaginal bleeding pattern of the combined contraceptive vaginal ring NuvaRing®. Eur J Obstet Gynecol Reprod Biol. 2008;139(1):65–71.

11. Chaovisitsaree S, Piyamongkol W, Pongsatha S, Morakote N, Noium S, Soonthornlimsiri N, et al. One year study of Implanon on the adverse events and discontinuation. J Med Assoc Thai. 2005;88(3):314–7.

12. Daud S, Ewies AA. Levonorgestrel-releasing intrauterine system: why do some women dislike it? Gynecol Endocrinol. 2008;24(12):686–90.

Clasificación de la information: General

13. Ekabua J, Itam I. The safety and complications of Norplant use in Calabar. Trop Doct. 2007;37(1):37–9.

14. Frederico G, Silva dos Santos PN, Ferreira JM, Bahamondes L, Fernandes A. Female body mass index and the selection of a long-acting reversible contraception for the first time. Int J Gynecol Obstet. 2022;158(3):748–53.

15. Friedman JO. Factors associated with contraceptive satisfaction in adolescent women using the IUD. J Pediatr Adolesc Gynecol. 2015;28(1):38–42.

16. Fruzzetti F, Perini D, Fornaciari L, Russo M, Bucci F, Gadducci A. Discontinuation of modern hormonal contraceptives: an Italian survey. Eur J Contracept Reprod Health Care. 2016;21(6):449–54.

17. Hajikazemi E, Nikpour S, Haghani H. Reasons for discontinuation of depot medroxyprogesterone acetate. In: International Congress Series. Elsevier; 2004. p. 315–8.

18. Hines GV, Quinones JN, Walker TN, Waxman A. Continuation Rates of the Etonogestrel Implant and Factors Associated With Early Discontinuation [A32]. Obstet Gynecol. 2022;139:10S.

19. Hofmeyr GJ, Singata-Madliki M, Lawrie TA, Bergel E, Temmerman M. Effects of the copper intrauterine device versus injectable progestin contraception on pregnancy rates and method discontinuation among women attending termination of pregnancy services in South Africa: a pragmatic randomized controlled trial. Reprod Health. 2016;13:1–8.

20. Iftikhar PM, Shaheen N, Arora E, Iftikhar P. Efficacy and satisfaction rate in postpartum intrauterine contraceptive device insertion: a prospective study. Cureus. 2019;11(9).

21. Keogh SC, Otupiri E, Castillo PW, Chiu DW, Polis CB, Nakua EK, et al. Hormonal contraceptive use in Ghana: the role of method attributes and side effects in method choice and continuation. Contraception. 2021;104(3):235–45.

22. Khader YS, El-Qaderi S, Khader AM. Intrauterine contraceptive device discontinuation among Jordanian women: rate, causes and determinants. BMJ Sex Reprod Health. 2006;32(3):161–4.

23. Kriplani A, Sehgal R, Konar H, Vivekanand A, Vanamail P, Purandare CN. A 1-year comparison of TC u380Ag versus TC u380A intrauterine contraceptive devices in India. Int J Gynecol Obstet. 2019;145(3):268–77.

24. Landolt NK, Phanuphak N, Teeratakulpisarn N, Kriengsinyot R, Ahluwalia J, Pinyakorn S, et al. Uptake and continuous use of copper intrauterine device in a cohort of HIV-positive women. AIDS Care. 2013;25(6):710–4.

25. Lathrop E, Hurst S, Mendoza Z, Zapata LB, Cordero P, Powell R, et al. Final program data and factors associated with long-acting reversible contraception removal: the Zika contraception access network. Obstet Gynecol. 2020;135(5):1095–103.

26. Lete I, Pérez-Campos E, Correa M, Robledo J, de la Viuda E, Martínez T, et al. Continuation rate of combined hormonal contraception: a prospective multicenter study. J Womens Health. 2012;21(5):490–5.

Clasificación de la information: General

27. Littlejohn KE. Hormonal contraceptive use and discontinuation because of dissatisfaction: differences by race and education. Demography. 2012;49(4):1433–52.

28. Madden T, Eisenberg DL, Zhao Q, Buckel C, Secura GM, Peipert JF. Continuation of the etonogestrel implant in women undergoing immediate postabortion placement. Obstet Gynecol. 2012;120(5):1053–9.

29. Merki-Feld GS, Hund M. Clinical experience with the combined contraceptive vaginal ring in Switzerland, including a subgroup analysis of previous hormonal contraceptive use. Eur J Contracept Reprod Health Care. 2010;15(6):413–22.

30. Mrwebi KP, Ter Goon D, Owolabi EO, Adeniyi OV, Seekoe E, Ajayi AI. Reasons for discontinuation of Implanon among users in Buffalo City Metropolitan Municipality, South Africa: a cross-sectional study. Afr J Reprod Health. 2018;22(1):113–9.

31. Park H. Assessing the relationship between adverse childhood experiences and body mass index trajectory of children and adolescents. Diss Abstr Int Sect Humanit Soc Sci. 2018;78(10-A(E)):No-Specified.

32. Parkpinyo N, Panichyawat N, Sirimai K. Early removal of the etonogestrel contraceptive implant and associated factors among users at the urban family planning clinic in Siriraj Hospital, Bangkok, Thailand. Siriraj Med J. 2021;73(6):399–405.

33. Regidor PA, Colli E, Palacios S. Overall and bleeding-related discontinuation rates of a new oral contraceptive containing 4 mg drospirenone only in a 24/4 regimen and comparison to 0.075 mg desogestrel. Gynecol Endocrinol. 2021;37(12):1121–7.

34. Rothschild CW, Richardson BA, Guthrie BL, Kithao P, Omurwa T, Mukabi J, et al. Contributions of side effects to contraceptive discontinuation and method switch among Kenyan women: a prospective cohort study. BJOG Int J Obstet Gynaecol. 2022;129(6):926–37.

35. Sabatini R, Cagiano R. Comparison profiles of cycle control, side effects and sexual satisfaction of three hormonal contraceptives. Contraception. 2006;74(3):220–3.

36. Saloranta TH, Gyllenberg FK, But A, Gissler M, Laine MK, Heikinheimo O. Free-of-charge long-acting reversible contraception: two-year discontinuation, its risk factors, and reasons. Am J Obstet Gynecol. 2020;223(6):886-e1.

37. Sarnak D, Gemmill A, Bradley SE, Brecker E, Patierno K. Stop or Switch: Correlates of Stopping Use or Switching Contraceptive Methods While Wanting to Avoid Pregnancy in 48 Low-and Middle-Income Countries. Stud Fam Plann. 2023;54(2):403–29.

38. Schafer JE, Osborne LM, Davis AR, Westhoff C. Acceptability and satisfaction using Quick Start with the contraceptive vaginal ring versus an oral contraceptive. Contraception. 2006;73(5):488–92.

39. Simmons RG, Sanders JN, Geist C, Gawron L, Myers K, Turok DK. Predictors of contraceptive switching and discontinuation within the first 6 months of use among Highly Effective Reversible Contraceptive Initiative Salt Lake study participants. Am J Obstet Gynecol. 2019;220(4):376-e1.

Clasificación de la information: General

40. Ssebatta G, Kaye DK, Mbalinda SN. Early contraceptive implants removal and its associated factors among women using implants at a National Referral Hospital, Kampala Uganda. BMC Womens Health. 2021;21:1–9.

41. Warner P, Guttinger A, Glasier A, Lee R, Nickerson S, Brenner R, et al. Randomized placebo-controlled trial of CDB-2914 in new users of a levonorgestrel-releasing intrauterine system shows only short-lived amelioration of unscheduled bleeding. Hum Reprod. 2010;25(2):345–53.

42. Wojcik N, Watkins L, Nugent R. Patient acceptability, continuation and complication rates with immediate postpartum levonorgestrel intrauterine device insertion at caesarean section and vaginal birth. Aust N Z J Obstet Gynaecol. 2022;62(5):773–8.

43. Alvergne A, Stevens R, Gurmu E. Side effects and the need for secrecy: characterising discontinuation of modern contraception and its causes in Ethiopia using mixed methods. Contracept Reprod Med. 2017;2:1–16.

44. Berglas NF, Kimport K, Mays A, Kaller S, Biggs MA. "It's Worked Well for Me": Young Women's Reasons for Choosing Lower-Efficacy Contraceptive Methods. J Pediatr Adolesc Gynecol. 2021;34(3):341–7.

45. Brunie A, Aw FNRS, Ndiaye S, Dioh E, Lebetkin E, Lydon MM, et al. Making removals part of informed choice: a mixed-method study of client experiences with removal of long-acting reversible contraceptives in Senegal. Glob Health Sci Pract. 2022;10(5).

46. Bryant AG, Gottert A, Stuart GS, Hamela G, Kamanga G. Reasons for intrauterine device use, discontinuation and non-use in Malawi: a qualitative study of women and their partners. Afr J Reprod Health. 2015;19(4):50–7.

47. Chin-Quee D, Diadhiou M, Eichleay M, Youssef A, Chen M, Bernholc A, et al. How much do side effects contribute to discontinuation? A longitudinal study of IUD and implant users in Senegal. Front Glob Womens Health. 2022;2:804135.

48. Coombe J, Harris ML, Loxton D. Motivators of contraceptive method change and implications for long-acting reversible contraception (non-) use: a qualitative free-text analysis. Sex Reprod Healthc. 2019;19:71–7.

49. Dalessandro C, Thorpe R, Sanders J. "I Just Don't Think I Can Deal:" Contraceptive Method Acceptability, Dealbreakers, and Women's Embodied Sense of Self. Sex Res Soc Policy. 2022;19(3):1046–57.

50. Epstein LB, Sokal-Gutierrez K, Ivey SL, Raine T, Auerswald C. Adolescent experiences with the vaginal ring. J Adolesc Health. 2008;43(1):64–70.

51. Gubrium A. "I've Lost My Mojo, Baby" A Narrative Perspective on the Effect of Depo-Provera on Libido. Sex Res Soc Policy. 2011;8:321–34.

52. Hoggart L, Newton VL. Young women's experiences of side-effects from contraceptive implants: a challenge to bodily control. Reprod Health Matters. 2013;21(41):196–204.

53. Imbuki K, Shaffer DN, Sinei SK, Todd CS, Stibich MA. Factors influencing contraceptive choice and discontinuation among HIV-positive women in Kericho, Kenya. Afr J Reprod Health. 2010;14(4):103–14.

Clasificación de la information: General

54. Khalaf IA. Exploring the use of modern contraceptive methods among Jordanian women: a qualitative study. Dirasat Med Biol Sci. 2004;31:46–66.

55. Kibira SP, Muhumuza C, Bukenya JN, Atuyambe LM. "I spent a full month bleeding, I thought I was going to die…" a qualitative study of experiences of women using modern contraception in Wakiso District, Uganda. PLoS One. 2015;10(11):e0141998.

56. Mills A, Barclay L. None of them were satisfactory: women's experiences with contraception. Health Care Women Int. 2006;27(5):379–98.

57. Mihretie GS, Abebe SM, Abera M, Assefa DT. An Interpretative Study of LARCs Discontinuation in Ethiopia: The Experiences of Women Accessing Contraceptives in Selected Public Health Facilities. Open Access J Contracept. 2023;41–51.

58. Mwizerwa J, Rozzano LC. The Lived Experience of Discontinuing Hormonal Contraception Among Women in Rural Uganda. Int J Hum Caring. 2011;15(1):56.

59. Nega G, Abera M, Tadele A. Discontinuation rate and associated factors among contraceptive implant users in Kersa district, southwestern Ethiopia. Arch Public Health. 2021;79(1):75.

60. Obare F, Odwe G, Cleland J. Men's needs and women's fears: gender-related power dynamics in contraceptive use and coping with consequences in a rural setting in Kenya. Cult Health Sex. 2021;23(12):1748–62.

61. Olaifa BT, Okonta HI, Mpinda JB, Govender I. Reasons given by women for discontinuing the use of progestogen implants at Koster Hospital, North West province. South Afr Fam Pract. 2022;64(4).

62. Ontiri S, Mutea L, Naanyu V, Kabue M, Biesma R, Stekelenburg J. A qualitative exploration of contraceptive use and discontinuation among women with an unmet need for modern contraception in Kenya. Reprod Health. 2021;18:1–10.

63. Undie CC, RamaRao S, Mbow FB. Choosing and using the progesterone vaginal ring: women's lived experiences in three African cities. Patient Prefer Adherence. 2020;1761–70.

64. Wigginton B, Harris ML, Loxton D, Herbert D, Lucke J. The feminisation of contraceptive use: Australian women's accounts of accessing contraception. Fem Psychol. 2015;25(2):178–98.

Clasificación de la information: General
