# Supplementary material for: The sex effect: the prevalence of sex life reasons for contraceptive discontinuation. A systematic review and meta-analysis
Source: Sex Reprod Health Matters. 2025 Sep 25;33(1):2552589. doi: 10.1080/26410397.2025.2552589 (PMC12557827; doi:10.1080/26410397.2025.2552589)
Supplement: Supplemental Material [file ZRHM_A_2552589_SM8760.docx]

**Supplementary Materials Content**

1. Search Strategy

2. Results from meta-regressions

3. Risk of Bias summary table

3.1 Quantitative studies

3.2 Qualitative studies

4. Supplementary figures and tables

5. Full references for included studies

1. **Search strategy**

At a high level our search strategy combines MeSH terms and keywords with the following logic: (Contraception) AND ((Discontinuation) OR (Switching)).

In order to further specify our search, we have manually selected filters that allows us to capture our time period of interest, retain only studies in humans (i.e. exclude animal studies) and in the appropriate age range (e.g. exclude babies, toddlers, young children), as well as retain relevant article types (e.g. exclude preprints).

The full PubMed search syntax:

Search: (Contraception[Mesh] OR "Contraception, Postcoital"[Mesh] OR "Contraception, Barrier"[Mesh] OR "Contraception, Immunologic"[Mesh] OR "Contraception Behavior"[Mesh] OR "Hormonal Contraception"[Mesh] OR "Long-Acting Reversible Contraception"[Mesh] OR "Contraceptive Effectiveness"[Mesh] OR "Reproductive Control Agents"[Mesh] OR "Medroxyprogesterone Acetate"[Mesh] OR Desogestrel[Mesh] OR "Contraceptives, Oral"[Mesh] OR "cervical cap"[Title/Abstract] OR "cervical caps"[Title/Abstract] OR "coiled spring*"[Title/Abstract] OR "vaginal ring*"[Title/Abstract] OR "vaginal diaphragm*"[Title/Abstract] OR "vaginal shield*"[Title/Abstract] OR "contraceptive device*"[Title/Abstract] OR "Intrauterine Device*"[Title/Abstract] OR IUD[Title/Abstract] OR IUS[Title/Abstract] OR “family plan*”[Title/Abstract] OR "reproductive control agent*"[Title/Abstract] OR abortifacient*[Title/Abstract] OR "Depo-Provera"[Title/Abstract] OR DMPA[Title/Abstract] OR "Oral Contraceptives Low-Dose"[Title/Abstract] OR "Oral contraceptive hormonal"[Title/Abstract] OR "Postcoital Contraceptive*"[Title/Abstract] OR "Emergency Contraceptive*"[Title/Abstract] OR "Emergency Contraceptive*"[Title/Abstract] OR "Morning After Pill"[Title/Abstract] OR "Morning-After Pill"[Title/Abstract] OR "Voluntary Female Sterilization*"[Title/Abstract] OR "Female Sterilization*"[Title/Abstract] OR "Male Sterilization*"[Title/Abstract] OR vasectomy[Title/Abstract]) AND (continu* OR discontinu* OR "discontinuation rate*" OR switch* OR reverse)

Filters: Classical Article, Clinical Study, Clinical Trial, Controlled Clinical Trial, Corrected and Republished Article, Meta-Analysis, Observational Study, Randomized Controlled Trial, Review, Systematic Review, Humans, English, Adolescent: 13-18 years, Adult: 19+ years, Young Adult: 19-24 years, Adult: 19-44 years, Middle Aged: 45-64 years, Exclude preprints, from 2004 – 2023

We have manually selected appropriate terms and adjusted syntax for each database. We have then used the Polyglot tool from <https://www.sr-accelerator.com/#/polyglot> to double-check our search and ensure we do not have syntax errors across the different databases.

1. **Results from meta-regressions**

| Model |  | Estimate (CI) | SE | -95% CI | +95% CI | z-value |
| --- | --- | --- | --- | --- | --- | --- |
| 1.Univariate meta-regression for hormonal content of examined method,  Reference level = Non-hormonal methods | | | | | | |
|  | Intercept | -3.37*** | 0.75 | -4.84, | -1.91 | -4.52 |
|  | Both | -0.39 | 1.21 | -2.75 | 1.98 | -0.32 |
|  | Hormonal | -0.59 | 0.80 | -0.98 | 2.17 | 0.74 |
|  | Unclear | -0.83 | 1.37 | -3.53 | 1.86 | -0.60 |
| 2.Univariate meta-regression for specific contraceptive method,  Reference level = Copper-IUD | | | | | | |
|  | Intercept | -3.34*** | 0.88 | -5.05 | -1.62 | -3.81 |
|  | Implant | 0.12 | 1.11 | -2.06 | 2.30 | 0.11 |
|  | Injection | 0.61 | 1.44 | -2.21 | 3.43 | 0.42 |
|  | LNG-IUS | 0.96 | 1.24 | -1.47 | 3.40 | 0.78 |
|  | Pill | -0.33 | 1.37 | -3.01 | 2.35 | -0.24 |
|  | Unclear | -0.87 | 1.70 | -4.19 | 2.46 | -0.51 |
|  | Vaginal ring | 1.63 | 1.47 | -1.25 | 4.52 | 1.11 |
|  | Various | 0.38 | 1.06 | -1.69 | 2.46 | 0.36 |
| 3. Univariate meta-regression for reason type by Higgins & Smith framework  Reference level = Sexual functioning, especially libido | | | | | | |
|  | Intercept | -3.16*** | 0.44 | -4.02 | -2.30 | -7.18 |
|  | Concern for partner’s pleasure and functioning | 0.51 | 0.78 | -1.02 | 2.04 | 0.66 |
|  | Pleasure and Pleasure seeking | -2.59 | 2.05 | -6.61 | 1.44 | -1.26 |
|  | Sexual preferences/ Sexual aesthetics | 0.41 | 0.10 | -1.55 | 2.37 | 0.41 |
|  | Unclear | 0.29 | 0.95 | -1.56 | 2.15 | 0.31 |
| 4. Univariate meta-regression for level of reason by Higgins & Smith framework  Reference level = Individual | | | | | | |
|  | Intercept | -3.17*** | 0.38 | -3.92 | -2.43 | -8.32 |
|  | Relationship | 0.52 | 0.74 | -0.93 | 1.98 | 0.71 |
|  | Unclear | 0.31 | 0.91 | -1.47 | 2.09 | 0.34 |
| 5. Univariate meta-regression for risk of bias / quality assessment  Reference level = Poor overall score | | | | | | |
|  | Intercept | -2.90*** | 0.57 | -5.04 | -4.03 | -1.78 |
|  | Fair | 0.04 | 0.74 | 0.05 | -1.41 | 1.49 |
|  | Good | -0.30 | 0.73 | 0.73 | -1.73 | 1.14 |
| 6. Multivariate regression, accounting for hormonal content of examined method and specific contraceptive method  Reference level = Non-hormonal | | | | | | |
|  | Intercept | -3.34*** | 0.86 | -5.03 | 1.65 | -3.88 |
|  | Both | -0.37 | 1.41 | -3.13 | 2.39 | -0.26 |
|  | Hormonal | 0.60 | 1.05 | -1.46 | 2.66 | 0.57 |
|  | Unclear (hormonal) | -0.86 | 1.66 | -4.11 | 2.38 | -0.52 |
|  | Implant | -0.39 | 0.97 | -2.29 | 1.50 | -0.41 |
|  | Injection | 0.04 | 1.27 | -2.45 | 2.53 | 0.03 |
|  | LNG-IUS | 0.62 | 1.25 | -1.84 | 3.07 | 0.49 |
|  | Pill | -0.92 | 1.19 | -3.25 | 1.41 | -0.77 |
|  | Vaginal ring | 1.35 | 1.68 | -1.94 | 4.65 | 0.81 |

For p-values: *** denotes p < .0001. All other p-values are above .05.

Although all meta-regression models are estimable, we caution that in some cases they are based on very small samples and very few datapoints (studies), and as such should be treated very tentatively.

For Model 1, Both refers to studies examining both hormonal and non-hormonal methods. Unclear refers to studies where it is unclear if the contraceptive method is hormonal or non-hormonal.

For Model 2, Unclear refers to studies where it is unclear what specific methods were used; Various refers to various methods examined in a single study, where it was not possible to disaggregate by specific method.

For Model 3, Unclear refers to studies where it is unclear what the relevant reason category per the Higgins & Smith (2016) framework was. Analogously, for Model 4 Unclear refers to studies where it is unclear what the level of relevant reason category per the Higgins & Smith (2016) framework was.

1. **Risk of Bias summary table**

3.1 Quantitative studies

When assessing cohort studies, we used the National Heart Lung and Blood Institute’s (NHLBI) Quality Assessment Tool for Observational Cohort and Cross-Sectional Studies. We relied on the NHLBI’s Quality Assessment of Controlled Intervention Studies in the case of non-randomized interventional studies. NHLBI tools produce an overall quality rating scored Good – Fair – Poor.

For randomized controlled studies, we used Cochrane’s Risk-of-bias tool for Randomized Trials (RoB 2). This tool’s overall score ranges from ‘Low risk of bias’ to ‘Some concerns’ to ‘High risk of bias’. To maintain uniformity, we have relabeled these scores to Poor – Fair – Good, respectively (e.g. High risk of bias is denoted as ‘Poor’ below).

The number after each study (e.g. (1), (2), etc.) allows for confirming the full reference of the study, see section 5 of this supplement.

| **Study** | **Screening tool** | **Overall** |
| --- | --- | --- |
| Al-Jefout et al. (2015) (1) | NHLBI | Poor |
| Armitage, Mitchell, Wigan, & Smith (2012) (2) | NHLBI | Poor |
| Awoyesuku, Altraide, & Amadi, (2021) (3) | NHLBI | Fair |
| Bachofner et al. (2018) (4) | NHLBI | Fair |
| Bameka, Kakaire, Kaye, & Namusoke (2023) (5) | NHLBI | Good |
| Barreiros et al. (2007) (6) | NHLBI | Good |
| Beyene et al. (2022) (7) | NHLBI | Good |
| Blumenthal, Gemzell-Danielsson, & Marintcheva-Petrova (2008) (8) | NHLBI | Poor |
| Brockmeyer et al. (2008) (9) | NHLBI | Good |
| Bruni et al. (2008) (10) | NHLBI | Fair |
| Chaovisitsaree et al. (2005) (11) | NHLBI | Poor |
| Daud & Ewies (2008) (12) | NHLBI | Fair |
| Ekabua & Itam (2007) (13) | NHLBI | Fair |
| Frederico, Silva dos Santos, Ferreira, Bahamondes, & Fernandes (2022) (14) | NHLBI | Good |
| Friedman (2015) (15) | NHLBI | Fair |
| Fruzzetti et al. (2016) (16) | NHLBI | Fair |
| Hajikazemi, Nikpour, Haghani (2004) (17) | NHLBI | Poor |
| Hines et al. (2022) (18) | NHLBI | Good |
| Hofmeyr et al. (2016) (19) | RoB - 2 | Poor |
| Iftikhar, Shaheen, Arora (2019) (20) | NHLBI | Poor |
| Keogh et al. (2021) (21) | NHLBI | Good |
| Khader, El-Qaderi, Khader (2006) (22) | NHLBI | Fair |
| Kriplani et al. (2019) (23) | RoB - 2 | Fair |
| Landolt et al. (2013) (24) | NHLBI | Good |
| Lathrop et al. (2020) (25) | NHLBI | Good |
| Lete et al. (2012) (26) | NHLBI | Fair |
| Littlejohn (2012) (27) | NHLBI | Good |
| Madden et al. (2012) (28) | NHLBI | Good |
| Merki-Feld & Hund (2010) (29) | NHLBI | Poor |
| Mrwebi et al. (2018) (30) | NHLBI | Good |
| Park, Nguyen & Ngo (2011) (31) | NHLBI | Good |
| Parkpinyo, Panichyawat, Sirimai (2021) (32) | NHLBI | Good |
| Regidor, Colli, Palacios (2021) (33) | RoB – 2 | Poor |
| Rothschild et al. (2022) (34) | NHLBI | Fair |
| Sabatini and Cagiano (2006) (35) | RoB - 2 | Fair |
| Saloranta et al. (2020) (36) | NHLBI | Good |
| Sarnak et al. (2023) (37) | NHLBI | Good |
| Schafer, Osborne, Davis & Westhoff, (2006) (38) | RoB - 2 | Poor |
| Simmons et al. (2019) (39) | NHLBI | Good |
| Ssebatta, Kaye, Mbalinda (2021) (40) | NHLBI | Fair |
| Warner et al. (2010) (41) | RoB - 2 | Good |
| Wojcik et al. (2022) (42) | NHLBI | Good |

3.2 Qualitative studies

Note some of the below studies are mixed-method and include quantitative and qualitative components. We have screened these studies with a qualitative design assessment tool, the Joanna Briggs Institute Critical Appraisal Tool for Qualitative Studies. This is because for all of the below studies, sex concerns as reasons for discontinuation are reported within the qualitative methodology. The Joanna Briggs Institute Critical Appraisal Tool for Qualitative Studies produces an overall appraisal that is scored as ‘Include’, ‘Exclude’, ‘Seek further info’. To maintain uniformity with the other tools we have used, we have re-labelled these scores as ‘Good’, ‘Poor’, and ‘Fair’ respectively.

The number after each study (e.g. (43), (44), etc.) allows for confirming the full reference of the study, see section 5 of this supplement.

| **Study** | **Overall score** |
| --- | --- |
| Alvergne, Stevens & Gurmu (2017) (43) | Fair |
| Berglas et al. (2021) (44) | Good |
| Brunie et al. (2022) (45) | Good |
| Bryant et al. (2015) (46) | Good |
| Chin-Quee et al. (2022) (47) | Good |
| Coombe, Harris, Loxton (2019) (48) | Good |
| Dalessandro, Thorpe, Sanders (2022) (49) | Good |
| Epstein (2008) (50) | Good |
| Gubrium (2011) (51) | Good |
| Hoggart & Newton (2013) (52) | Good |
| Imbuki et al. (2010) (53) | Fair |
| Khalaf (2004) (54) | Good |
| Kibira, Muhumuza, Bukenya, Atuyambe (2015) (55) | Good |
| Mills & Barclay (2006) (56) | Fair |
| Mihretie et al. (2023) (57) | Good |
| Mwizerwa & Rozzano (2011) (58) | Poor |
| Nega, Abera, Tadele (2021) (59) | Poor |
| Obare, Odwe, & Cleland (2021) (60) | Fair |
| Olaifa et al. (2022) (61) | Good |
| Ontiri et al. (2021) (62) | Good |
| Undie, RamaRao, Mbow (2020) (63) | Good |
| Wigginton et al. (2015) (64) | Good |

1. **Supplementary figures and tables**


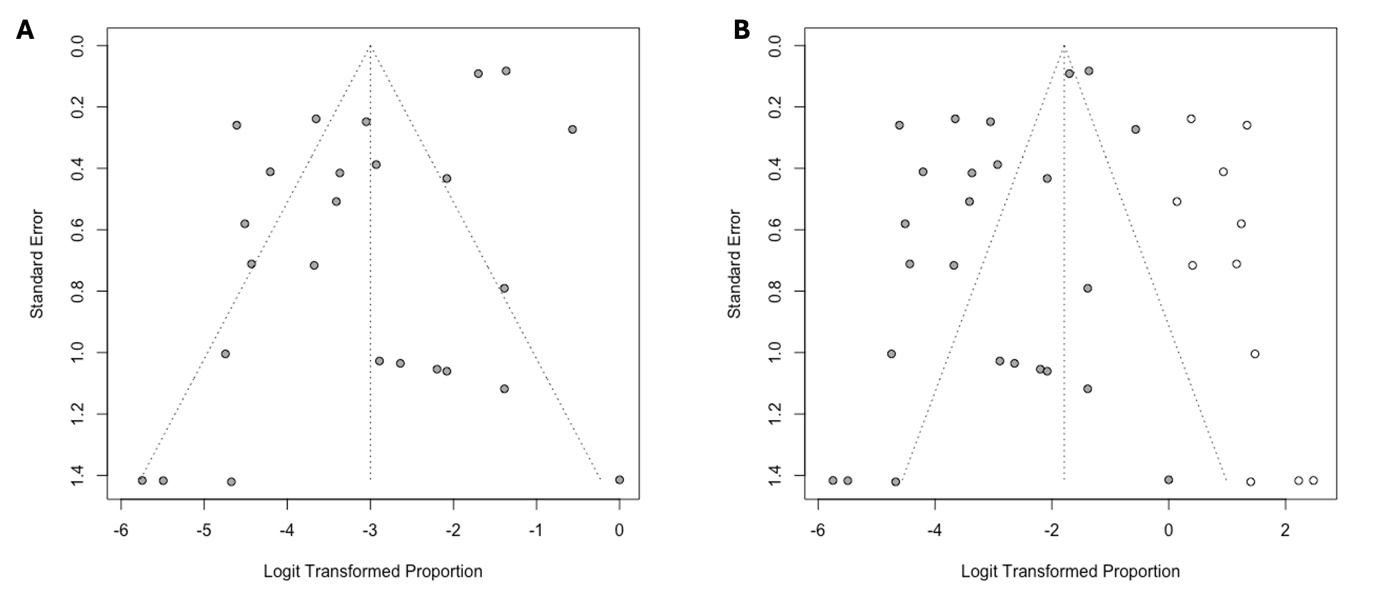
Figure 1. Panel A. Funnel plot. Panel B. Funnel plot following trim and fill procedure.


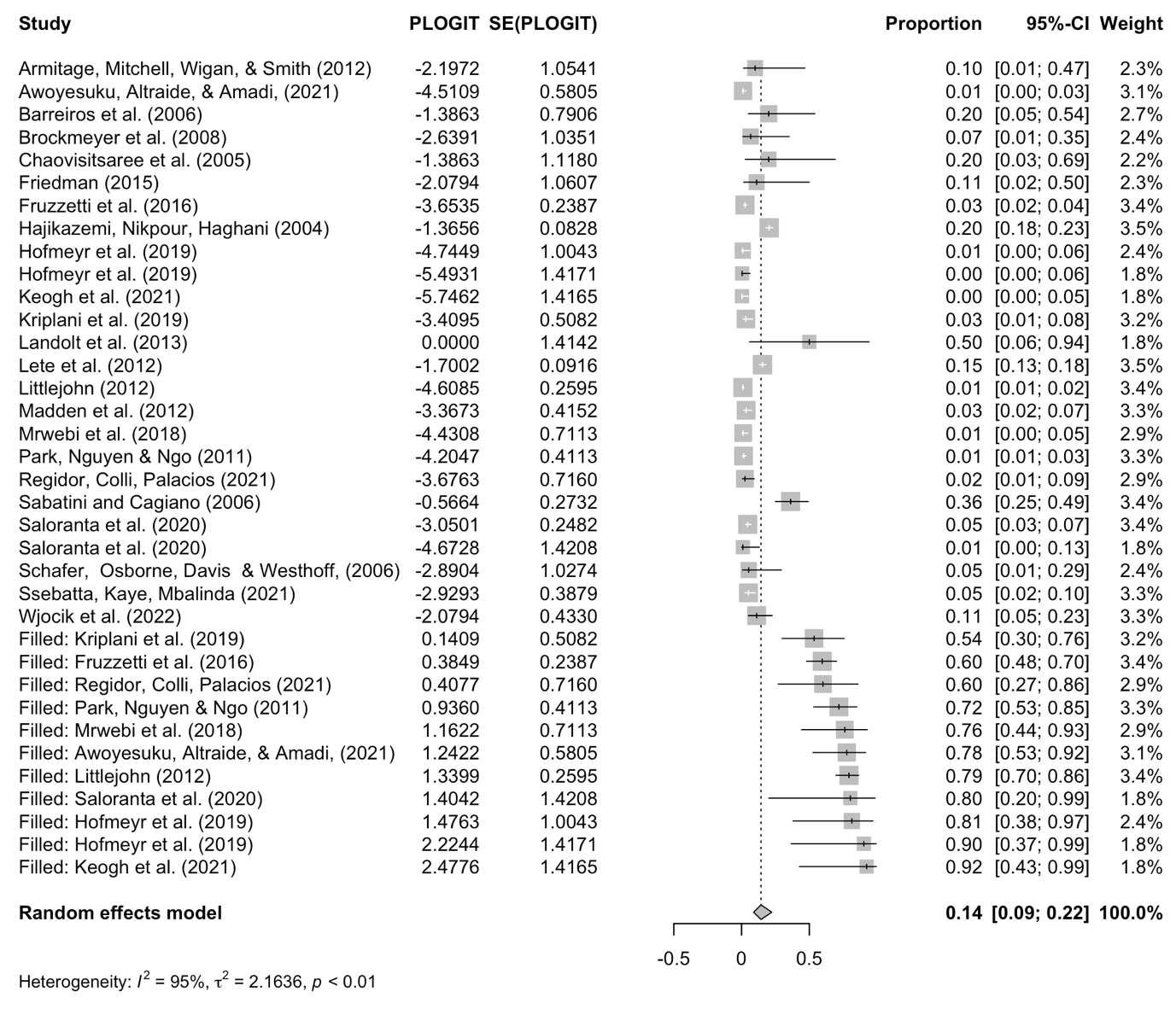
Figure 2. Forest plot following trim and fill procedures.


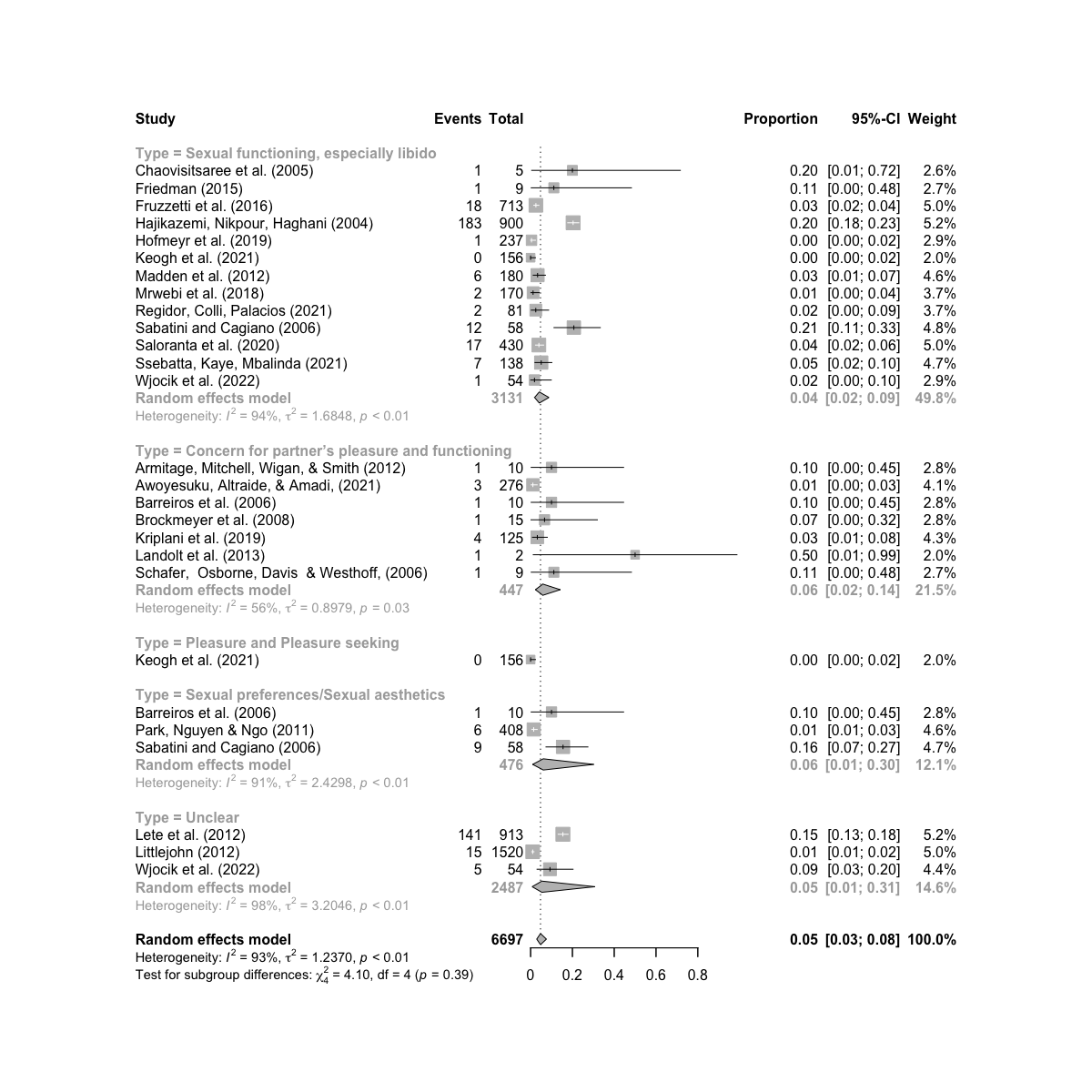
Figure 3. Forest plot presenting results for sub-group analyses based on the specific category of reasons according to the Higgins & Smith (2016) framework.


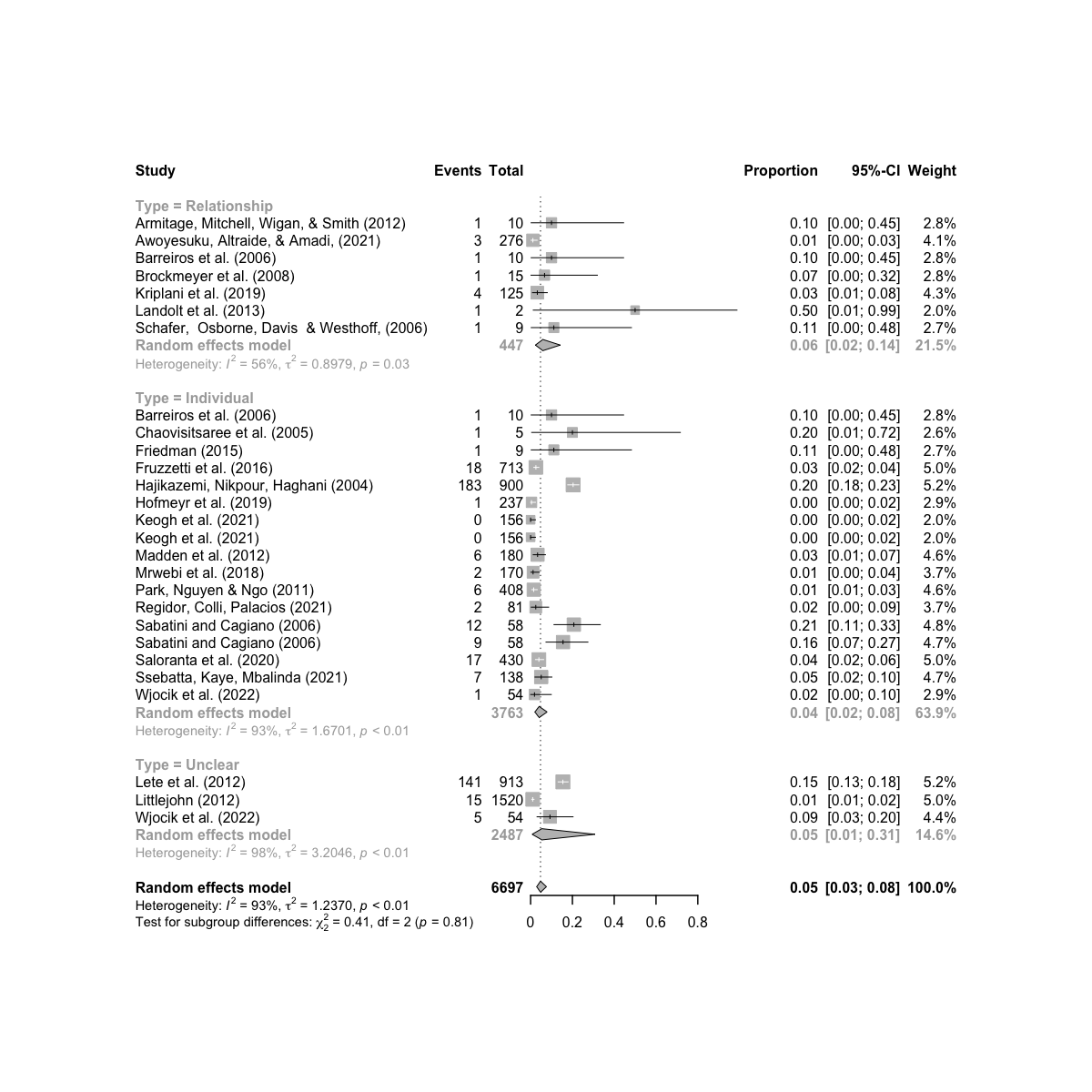
Figure 4. Forest plot presenting results for sub-group analyses based on the specific level of reasons according to the Higgins & Smith (2016) framework.


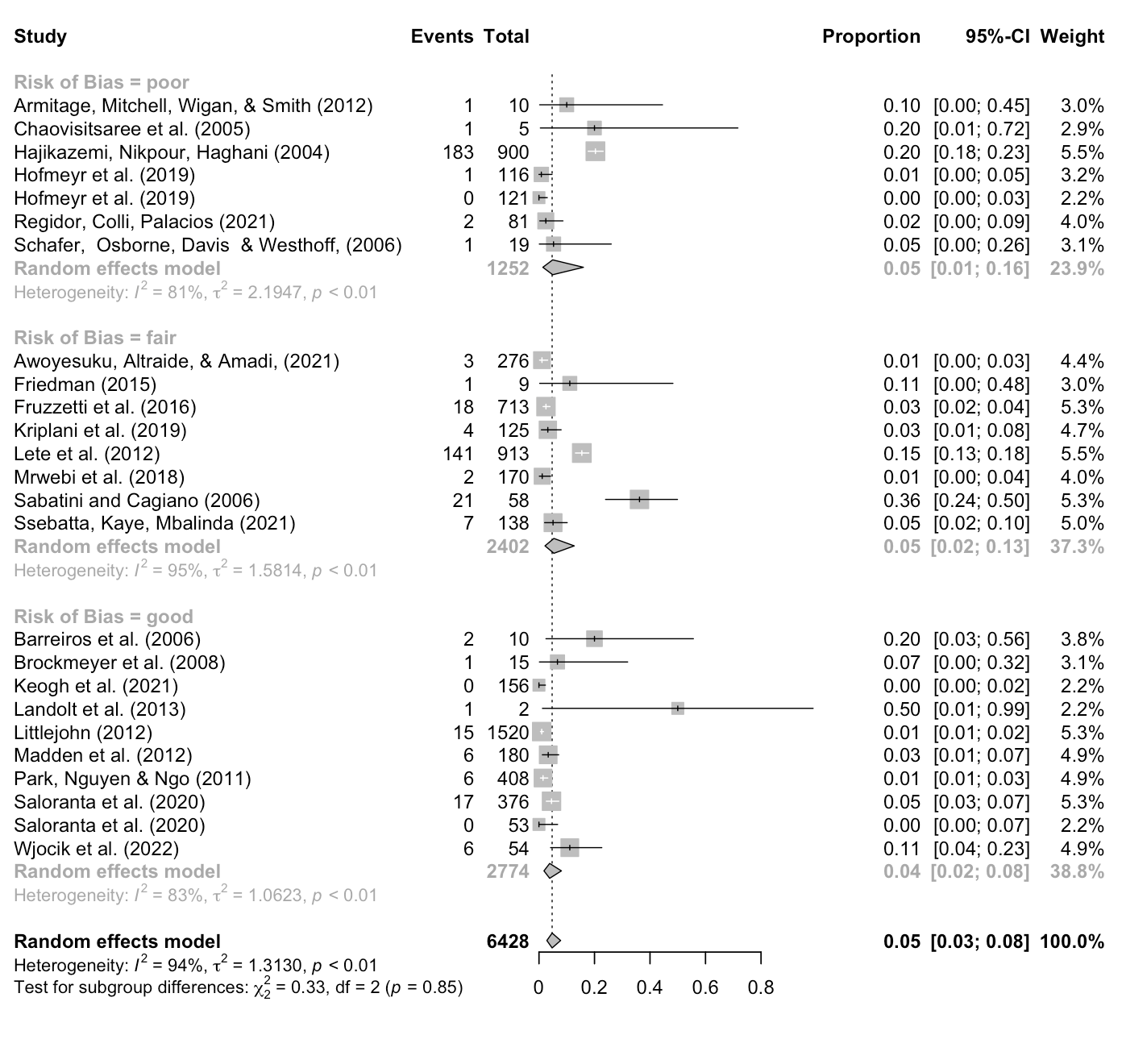
Figure 5. Forest plot presenting results for sub-group analyses based on studies’ overall risk of bias score.

1. **Full references for included studies**

1. Al-Jefout M, Nawaiseh N, Tashman S, Ryalat R, Zaitoun S, Al-Alawi L, et al. Jordanian Women’s Experience with Etonogestrel Subdermal Contraceptive Implant in Two Family Planning Clinics. Jordan Med J. 2015;49(1):27–35.

2. Armitage CM, Mitchell C, Wigan C, Smith DA. Uptake and continuation rates of the intrauterine system in a university student general practice population in the UK. J Fam Plann Reprod Health Care. 2013;39(3):186–9.

3. Awoyesuku PA, Altraide BO, Amadi SC. Modern contraceptives discontinuation, method switching and associated factors among clients at the family planning clinic of a tertiary hospital in Port-Harcourt, Nigeria. Int J Reprod Contracept Obstet Gynecol. 2021;10(1):6.

4. Bachofner M, Blickenstorfer K, Hutmacher J, Wehrle L, Leeners B, Merki-Feld G. Intrauterine device continuation rates and reasons for discontinuation in a Central European clinic with a high standard of care and ultrasound follow-up: a retrospective cohort study. Eur J Contracept Reprod Health Care. 2018;23(6):407–14.

5. Bameka A, Kakaire O, Kaye DK, Namusoke F. Early discontinuation of long-acting reversible contraceptives and associated factors among women discontinuing long-acting reversible contraceptives at national referral hospital, Kampala-Uganda; a cross-sectional study. Contracept Reprod Med. 2023;8(1):27.

6. Barreiros FA, Guazzelli CAF, de Araújo FF, Barbosa R. Bleeding patterns of women using extended regimens of the contraceptive vaginal ring. Contraception. 2007;75(3):204–8.

7. Beyene GN, Assefa N, Mokonnon TM, Ejigu HB, Yadeta TA. Early Implanon discontinuation and associated factors among Implanon women users visiting public health facilities, in Kembata zone of Southern Ethiopia: An institution based cross-sectional study. Front Glob Womens Health. 2022;3:909411.

8. Blumenthal PD, Gemzell-Danielsson K, Marintcheva-Petrova M. Tolerability and clinical safety of Implanon®. Eur J Contracept Reprod Health Care. 2008;13(sup1):29–36.

9. Brockmeyer A, Kishen M, Webb A. Experience of IUD/IUS insertions and clinical performance in nulliparous women–a pilot study. Eur J Contracept Reprod Health Care. 2008;13(3):248–54.

10. Bruni V, Pontello V, Luisi S, Petraglia F. An open-label, multicentre trial to evaluate the vaginal bleeding pattern of the combined contraceptive vaginal ring NuvaRing®. Eur J Obstet Gynecol Reprod Biol. 2008;139(1):65–71.

11. Chaovisitsaree S, Piyamongkol W, Pongsatha S, Morakote N, Noium S, Soonthornlimsiri N, et al. One year study of Implanon on the adverse events and discontinuation. J Med Assoc Thai. 2005;88(3):314–7.

12. Daud S, Ewies AA. Levonorgestrel-releasing intrauterine system: why do some women dislike it? Gynecol Endocrinol. 2008;24(12):686–90.

13. Ekabua J, Itam I. The safety and complications of Norplant use in Calabar. Trop Doct. 2007;37(1):37–9.

14. Frederico G, Silva dos Santos PN, Ferreira JM, Bahamondes L, Fernandes A. Female body mass index and the selection of a long-acting reversible contraception for the first time. Int J Gynecol Obstet. 2022;158(3):748–53.

15. Friedman JO. Factors associated with contraceptive satisfaction in adolescent women using the IUD. J Pediatr Adolesc Gynecol. 2015;28(1):38–42.

16. Fruzzetti F, Perini D, Fornaciari L, Russo M, Bucci F, Gadducci A. Discontinuation of modern hormonal contraceptives: an Italian survey. Eur J Contracept Reprod Health Care. 2016;21(6):449–54.

17. Hajikazemi E, Nikpour S, Haghani H. Reasons for discontinuation of depot medroxyprogesterone acetate. In: International Congress Series. Elsevier; 2004. p. 315–8.

18. Hines GV, Quinones JN, Walker TN, Waxman A. Continuation Rates of the Etonogestrel Implant and Factors Associated With Early Discontinuation [A32]. Obstet Gynecol. 2022;139:10S.

19. Hofmeyr GJ, Singata-Madliki M, Lawrie TA, Bergel E, Temmerman M. Effects of the copper intrauterine device versus injectable progestin contraception on pregnancy rates and method discontinuation among women attending termination of pregnancy services in South Africa: a pragmatic randomized controlled trial. Reprod Health. 2016;13:1–8.

20. Iftikhar PM, Shaheen N, Arora E, Iftikhar P. Efficacy and satisfaction rate in postpartum intrauterine contraceptive device insertion: a prospective study. Cureus. 2019;11(9).

21. Keogh SC, Otupiri E, Castillo PW, Chiu DW, Polis CB, Nakua EK, et al. Hormonal contraceptive use in Ghana: the role of method attributes and side effects in method choice and continuation. Contraception. 2021;104(3):235–45.

22. Khader YS, El-Qaderi S, Khader AM. Intrauterine contraceptive device discontinuation among Jordanian women: rate, causes and determinants. BMJ Sex Reprod Health. 2006;32(3):161–4.

23. Kriplani A, Sehgal R, Konar H, Vivekanand A, Vanamail P, Purandare CN. A 1-year comparison of TC u380Ag versus TC u380A intrauterine contraceptive devices in India. Int J Gynecol Obstet. 2019;145(3):268–77.

24. Landolt NK, Phanuphak N, Teeratakulpisarn N, Kriengsinyot R, Ahluwalia J, Pinyakorn S, et al. Uptake and continuous use of copper intrauterine device in a cohort of HIV-positive women. AIDS Care. 2013;25(6):710–4.

25. Lathrop E, Hurst S, Mendoza Z, Zapata LB, Cordero P, Powell R, et al. Final program data and factors associated with long-acting reversible contraception removal: the Zika contraception access network. Obstet Gynecol. 2020;135(5):1095–103.

26. Lete I, Pérez-Campos E, Correa M, Robledo J, de la Viuda E, Martínez T, et al. Continuation rate of combined hormonal contraception: a prospective multicenter study. J Womens Health. 2012;21(5):490–5.

27. Littlejohn KE. Hormonal contraceptive use and discontinuation because of dissatisfaction: differences by race and education. Demography. 2012;49(4):1433–52.

28. Madden T, Eisenberg DL, Zhao Q, Buckel C, Secura GM, Peipert JF. Continuation of the etonogestrel implant in women undergoing immediate postabortion placement. Obstet Gynecol. 2012;120(5):1053–9.

29. Merki-Feld GS, Hund M. Clinical experience with the combined contraceptive vaginal ring in Switzerland, including a subgroup analysis of previous hormonal contraceptive use. Eur J Contracept Reprod Health Care. 2010;15(6):413–22.

30. Mrwebi KP, Ter Goon D, Owolabi EO, Adeniyi OV, Seekoe E, Ajayi AI. Reasons for discontinuation of Implanon among users in Buffalo City Metropolitan Municipality, South Africa: a cross-sectional study. Afr J Reprod Health. 2018;22(1):113–9.

31. Park H. Assessing the relationship between adverse childhood experiences and body mass index trajectory of children and adolescents. Diss Abstr Int Sect Humanit Soc Sci. 2018;78(10-A(E)):No-Specified.

32. Parkpinyo N, Panichyawat N, Sirimai K. Early removal of the etonogestrel contraceptive implant and associated factors among users at the urban family planning clinic in Siriraj Hospital, Bangkok, Thailand. Siriraj Med J. 2021;73(6):399–405.

33. Regidor PA, Colli E, Palacios S. Overall and bleeding-related discontinuation rates of a new oral contraceptive containing 4 mg drospirenone only in a 24/4 regimen and comparison to 0.075 mg desogestrel. Gynecol Endocrinol. 2021;37(12):1121–7.

34. Rothschild CW, Richardson BA, Guthrie BL, Kithao P, Omurwa T, Mukabi J, et al. Contributions of side effects to contraceptive discontinuation and method switch among Kenyan women: a prospective cohort study. BJOG Int J Obstet Gynaecol. 2022;129(6):926–37.

35. Sabatini R, Cagiano R. Comparison profiles of cycle control, side effects and sexual satisfaction of three hormonal contraceptives. Contraception. 2006;74(3):220–3.

36. Saloranta TH, Gyllenberg FK, But A, Gissler M, Laine MK, Heikinheimo O. Free-of-charge long-acting reversible contraception: two-year discontinuation, its risk factors, and reasons. Am J Obstet Gynecol. 2020;223(6):886-e1.

37. Sarnak D, Gemmill A, Bradley SE, Brecker E, Patierno K. Stop or Switch: Correlates of Stopping Use or Switching Contraceptive Methods While Wanting to Avoid Pregnancy in 48 Low-and Middle-Income Countries. Stud Fam Plann. 2023;54(2):403–29.

38. Schafer JE, Osborne LM, Davis AR, Westhoff C. Acceptability and satisfaction using Quick Start with the contraceptive vaginal ring versus an oral contraceptive. Contraception. 2006;73(5):488–92.

39. Simmons RG, Sanders JN, Geist C, Gawron L, Myers K, Turok DK. Predictors of contraceptive switching and discontinuation within the first 6 months of use among Highly Effective Reversible Contraceptive Initiative Salt Lake study participants. Am J Obstet Gynecol. 2019;220(4):376-e1.

40. Ssebatta G, Kaye DK, Mbalinda SN. Early contraceptive implants removal and its associated factors among women using implants at a National Referral Hospital, Kampala Uganda. BMC Womens Health. 2021;21:1–9.

41. Warner P, Guttinger A, Glasier A, Lee R, Nickerson S, Brenner R, et al. Randomized placebo-controlled trial of CDB-2914 in new users of a levonorgestrel-releasing intrauterine system shows only short-lived amelioration of unscheduled bleeding. Hum Reprod. 2010;25(2):345–53.

42. Wojcik N, Watkins L, Nugent R. Patient acceptability, continuation and complication rates with immediate postpartum levonorgestrel intrauterine device insertion at caesarean section and vaginal birth. Aust N Z J Obstet Gynaecol. 2022;62(5):773–8.

43. Alvergne A, Stevens R, Gurmu E. Side effects and the need for secrecy: characterising discontinuation of modern contraception and its causes in Ethiopia using mixed methods. Contracept Reprod Med. 2017;2:1–16.

44. Berglas NF, Kimport K, Mays A, Kaller S, Biggs MA. “It’s Worked Well for Me”: Young Women’s Reasons for Choosing Lower-Efficacy Contraceptive Methods. J Pediatr Adolesc Gynecol. 2021;34(3):341–7.

45. Brunie A, Aw FNRS, Ndiaye S, Dioh E, Lebetkin E, Lydon MM, et al. Making removals part of informed choice: a mixed-method study of client experiences with removal of long-acting reversible contraceptives in Senegal. Glob Health Sci Pract. 2022;10(5).

46. Bryant AG, Gottert A, Stuart GS, Hamela G, Kamanga G. Reasons for intrauterine device use, discontinuation and non-use in Malawi: a qualitative study of women and their partners. Afr J Reprod Health. 2015;19(4):50–7.

47. Chin-Quee D, Diadhiou M, Eichleay M, Youssef A, Chen M, Bernholc A, et al. How much do side effects contribute to discontinuation? A longitudinal study of IUD and implant users in Senegal. Front Glob Womens Health. 2022;2:804135.

48. Coombe J, Harris ML, Loxton D. Motivators of contraceptive method change and implications for long-acting reversible contraception (non-) use: a qualitative free-text analysis. Sex Reprod Healthc. 2019;19:71–7.

49. Dalessandro C, Thorpe R, Sanders J. “I Just Don’t Think I Can Deal:” Contraceptive Method Acceptability, Dealbreakers, and Women’s Embodied Sense of Self. Sex Res Soc Policy. 2022;19(3):1046–57.

50. Epstein LB, Sokal-Gutierrez K, Ivey SL, Raine T, Auerswald C. Adolescent experiences with the vaginal ring. J Adolesc Health. 2008;43(1):64–70.

51. Gubrium A. “I’ve Lost My Mojo, Baby” A Narrative Perspective on the Effect of Depo-Provera on Libido. Sex Res Soc Policy. 2011;8:321–34.

52. Hoggart L, Newton VL. Young women’s experiences of side-effects from contraceptive implants: a challenge to bodily control. Reprod Health Matters. 2013;21(41):196–204.

53. Imbuki K, Shaffer DN, Sinei SK, Todd CS, Stibich MA. Factors influencing contraceptive choice and discontinuation among HIV-positive women in Kericho, Kenya. Afr J Reprod Health. 2010;14(4):103–14.

54. Khalaf IA. Exploring the use of modern contraceptive methods among Jordanian women: a qualitative study. Dirasat Med Biol Sci. 2004;31:46–66.

55. Kibira SP, Muhumuza C, Bukenya JN, Atuyambe LM. “I spent a full month bleeding, I thought I was going to die…” a qualitative study of experiences of women using modern contraception in Wakiso District, Uganda. PLoS One. 2015;10(11):e0141998.

56. Mills A, Barclay L. None of them were satisfactory: women’s experiences with contraception. Health Care Women Int. 2006;27(5):379–98.

57. Mihretie GS, Abebe SM, Abera M, Assefa DT. An Interpretative Study of LARCs Discontinuation in Ethiopia: The Experiences of Women Accessing Contraceptives in Selected Public Health Facilities. Open Access J Contracept. 2023;41–51.

58. Mwizerwa J, Rozzano LC. The Lived Experience of Discontinuing Hormonal Contraception Among Women in Rural Uganda. Int J Hum Caring. 2011;15(1):56.

59. Nega G, Abera M, Tadele A. Discontinuation rate and associated factors among contraceptive implant users in Kersa district, southwestern Ethiopia. Arch Public Health. 2021;79(1):75.

60. Obare F, Odwe G, Cleland J. Men’s needs and women’s fears: gender-related power dynamics in contraceptive use and coping with consequences in a rural setting in Kenya. Cult Health Sex. 2021;23(12):1748–62.

61. Olaifa BT, Okonta HI, Mpinda JB, Govender I. Reasons given by women for discontinuing the use of progestogen implants at Koster Hospital, North West province. South Afr Fam Pract. 2022;64(4).

62. Ontiri S, Mutea L, Naanyu V, Kabue M, Biesma R, Stekelenburg J. A qualitative exploration of contraceptive use and discontinuation among women with an unmet need for modern contraception in Kenya. Reprod Health. 2021;18:1–10.

63. Undie CC, RamaRao S, Mbow FB. Choosing and using the progesterone vaginal ring: women’s lived experiences in three African cities. Patient Prefer Adherence. 2020;1761–70.

64. Wigginton B, Harris ML, Loxton D, Herbert D, Lucke J. The feminisation of contraceptive use: Australian women’s accounts of accessing contraception. Fem Psychol. 2015;25(2):178–98.
